# Structural Requirements for Dihydrobenzoxazepinone Anthelmintics: Actions against Medically Important and Model Parasites: *Trichuris muris*, *Brugia malayi*, *Heligmosomoides polygyrus*, and *Schistosoma mansoni*

**DOI:** 10.1021/acsinfecdis.1c00025

**Published:** 2021-04-02

**Authors:** Frederick A. Partridge, Carole J.R. Bataille, Ruth Forman, Amy E. Marriott, Josephine Forde-Thomas, Cécile Häberli, Ria L. Dinsdale, James D.B. O’Sullivan, Nicky J. Willis, Graham M. Wynne, Helen Whiteland, John Archer, Andrew Steven, Jennifer Keiser, Joseph D. Turner, Karl F. Hoffmann, Mark J. Taylor, Kathryn J. Else, Angela J. Russell, David B. Sattelle

**Affiliations:** † Centre for Respiratory Biology, UCL Respiratory, Division of Medicine, University College London, London WC1E 6JF, United Kingdom; ‡ Department of Chemistry, Chemistry Research Laboratory, University of Oxford, Oxford OX1 3TA, United Kingdom; § Lydia Becker Institute of Immunology and Inflammation, Faculty of Biology, Medicine and Health, University of Manchester, Manchester M13 9PT, United Kingdom; ∥ Centre for Drugs and Diagnostics, Department of Tropical Disease Biology, Liverpool School of Tropical Medicine, Liverpool L3 5QA, United Kingdom; ⊥ Institute of Biological, Environmental and Rural Sciences (IBERS), Aberystwyth University, Aberystwyth, Wales SY23 3DA, United Kingdom; # Department of Medical Parasitology and Infection Biology, Swiss Tropical and Public Health Institute, Socinstrasse 57, Basel CH-4002, Switzerland; ¶ University of Basel, Petersplatz 1, Basel CH-4001, Switzerland; □ Henry Royce Institute, The University of Manchester, Oxford Road, Manchester M13 9PL, United Kingdom; ○ Alzheimer’s Research UK UCL Drug Discovery Institute, University College London, Gower Street, London WC1E 6BT, United Kingdom; △ Centre for Neglected Tropical Diseases, Liverpool School of Tropical Medicine, Liverpool L3 5QA, United Kingdom; ▽ Department of Pharmacology, University of Oxford, Mansfield Road, Oxford OX1 3QT, United Kingdom

**Keywords:** anthelmintic, *Trichuris*, whipworm, nematode, trematode, drug discovery

## Abstract

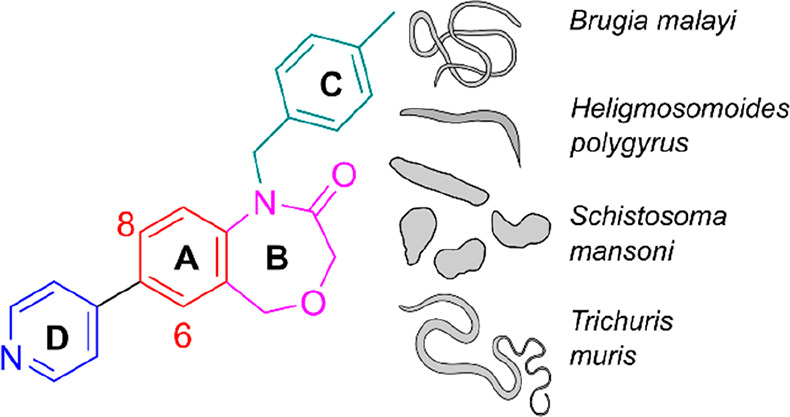

Nine hundred million people are infected with the soil-transmitted helminths *Ascaris lumbricoides* (roundworm), hookworm, and *Trichuris trichiura* (whipworm). However, low single-dose cure rates of the benzimidazole drugs, the mainstay of preventative chemotherapy for whipworm, together with parasite drug resistance, mean that current approaches may not be able to eliminate morbidity from trichuriasis. We are seeking to develop new anthelmintic drugs specifically with activity against whipworm as a priority and previously identified a hit series of dihydrobenzoxazepinone (DHB) compounds that block motility of *ex vivo Trichuris muris.* Here, we report a systematic investigation of the structure–activity relationship of the anthelmintic activity of DHB compounds. We synthesized 47 analogues, which allowed us to define features of the molecules essential for anthelmintic action as well as broadening the chemotype by identification of dihydrobenzoquinolinones (DBQs) with anthelmintic activity. We investigated the activity of these compounds against other parasitic nematodes, identifying DHB compounds with activity against *Brugia malayi* and *Heligmosomoides polygyrus*. We also demonstrated activity of DHB compounds against the trematode *Schistosoma mansoni,* a parasite that causes schistosomiasis. These results demonstrate the potential of DHB and DBQ compounds for further development as broad-spectrum anthelmintics.

Nine hundred million people are infected with soil-transmitted helminths, causing a global burden of around two million disability-adjusted life years.^1,2^ Because of this, the World Health Organization has set a goal to achieve and maintain elimination of soil-transmitted helminth morbidity by 2030.^3^ Huge mass drug administration efforts are underway, distributing hundreds of millions of doses of benzimidazole drugs (albendazole and mebendazole, Figure 1) to school-age children in affected areas annually or biannually as preventative chemotherapy (PCT).

**Figure 1:**
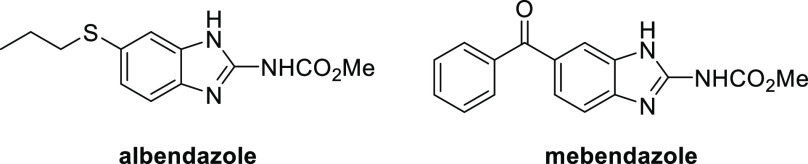
Structures of albendazole and mebendazole.

Benzimidazole drugs are partially effective against whipworm (*Trichuris trichiura*) when administered as a course of treatment, reaching cure rates of around 43%.^4^ However, for mass drug administration, practicalities and scale mean that only one dose is given. In contrast to *Ascaris lumbricoides*, where a single dose of benzimidazole drugs cures around 90–95% of infected individuals, the single dose cure rate for whipworm is low, around 30%.^5,6^ The current mass drug administration protocol may therefore not be able to break transmission and reduce the prevalence of moderate to heavy whipworm infections to below 2% as required to eliminate morbidity.^3^ Due to the poor single dose efficacy of the benzimidazole drugs against whipworm, there have been extensive efforts to identify more efficacious drug combinations.^6^ Of these, the most promising to date is a combination of albendazole plus the N-type nicotinic acetylcholine receptor agonist oxantel pamoate, which has a single dose cure rate reported to be between 31 and 83%.^7−10^ Albendazole plus ivermectin is the only approved drug combination for whipworm with a single dose cure rate of approximately 60%.^6^ A second drug, moxidectin, also shows promise to be added to albendazole for improved control of whipworm.^7^

Of concern, however, is the possibility that drug resistance may become prevalent, derailing the push toward control of whipworm. Currently, there is only indirect evidence of this possibility. In a meta-analysis, it has been shown that egg reduction rates and cure rates after albendazole treatment have been decreasing over time.^11^ Polymorphisms in the beta-tubulin gene that are associated with benzimidazole resistance are found in populations of human whipworm, and the frequency of these polymorphisms increased after albendazole treatment.^12,13^

The problem of resistance to anthelmintics is emphasized by the experience from veterinary medicine. Indeed, all currently approved anthelmintics were discovered primarily for the animal health market, with the key drugs levamisole, mebendazole, albendazole, and ivermectin approved between 1968 and 1981.^14^ Unfortunately, resistance in target species was reported rapidly after they entered use, most notably in the important sheep and goat parasite *Haemonchus contortus*.^15^ More recently, new drug classes such as the amino-acetonitrile derivative monepantel have been introduced, but resistance again emerged swiftly, within six years of farm use.^16^ Although there are some differences, such as treatment frequency, the same selective pressure that drives anthelmintic resistance on farms acts on the populations of human parasitic helminths that are exposed to incompletely effective doses of anthelmintics on a huge scale in mass drug administration (MDA) programs.^17^

Because of these two problems—low efficacy of existing drugs against whipworm and concerns about development of resistance to these drugs—we and others have been pursuing a strategy of identifying new antiwhipworm compounds via a mixture of repurposing and *de novo* small molecule screening.^18−25^ Underscoring the need for this work, the recent WHO roadmap for neglected tropical diseases 2021–2030 assesses that developing more effective medicines and drug combinations against *T. trichiura* and hookworm is a critical action required to meet the target for elimination of soil-transmitted helminths as a public health problem by 2030.^26^

Beyond soil transmitted helminths (STH), lymphatic filariasis and schistosomiasis are two medically important tissue helminthiases prioritized for global or regional elimination as a public health problem via preventative chemotherapy (PCT), as outlined in the WHO Roadmap 2030 implementation targets.^26^ A related filarial nematode, *Onchocerca volvulus*, is also targeted for regional elimination and interruption of transmission.

Lymphatic filariasis is caused by infection with the nematode parasites *Brugia malayi*, *B. timori*, and *Wuchereria bancrofti*. Current PCT uses a combination of albendazole, diethylcarbamazine citrate (DEC), and ivermectin, which has been shown to have a partial macrofilaricidal effect against *W. bancrofti*.^27,28^ Unfortunately, in countries where onchocerciasis is endemic, DEC is contraindicated, and where *Loa loa* is found, ivermectin is contraindicated. This prevents use of the triple therapy as PCT in some regions, and ideally, a new drug that can achieve macrofilaricidal (i.e., curative) efficacy but is safe to administrate in the context of loiasis and onchocerciasis will be developed. One promising therapeutic strategy is antibiotics that target the *Wolbachia* endosymbiont.^29^

Onchocerciasis is caused by another filarial nematode, *O. volvulus.* The current recommendation for PCT is once or twice yearly ivermectin sustained for ten or more years.^26^ Recently, moxidectin has been shown to be superior to ivermectin as microfilaricide.^30^ However, moxidectin, like ivermectin, is a macrocyclic lactone, and as demonstrated in veterinary medicine and model systems, cross-resistance is likely.^31^ There is promise for the future, with emodepside, oxfendazole, and the anti-*Wolbachia* agents ABBV-4083 and AWZ1066 in early clinical development.^32−34^

Moving beyond nematodes, schistosomiasis is caused by infection with *Schistosoma* trematodes such as *S. mansoni.* There are two types of disease: intestinal schistosomiasis and urogenital schistosomiasis, causing a burden of around 2.5 million disability-adjusted life years (DALYs).^26^ Praziquantel is the only drug used for treatment of human schistosomiasis. The mechanism of praziquantel action remained unclear for a long time, but recently, it has shown to be an activator of a transient receptor potential (TRP) channel.^35^

Reliance of current PCT protocols on a few, or in the case of onchocerciasis and schistosomiasis, a single chemotherapeutic agent (ivermectin, potentially replaced by moxidectin, and praziquantel, respectively) is a vulnerability of current elimination strategies, considering the potential for development of drug resistance. As with STH, annual or semiannual mass drug administrations extending upward of 20 years are required to break transmission with current drugs due to incomplete adulticidal/selective larvicidal activity profiles of the implemented antifilarial or schistosomicidal agents. Alternative strategies, for instance, development of a short-course curative treatment for filariasis, would be a step-change to reduce elimination time frames.^36,37^ Schistosome resistance to praziquantel can be induced after sublethal drug treatment of parasites in the laboratory, and reduced susceptibility of *S. mansoni* to the drug has been reported in the field.^38^ Again, the WHO has assessed that developing new, alternative medicines to complement praziquantel in case of resistance is a critical action to achieve the 2030 roadmap goal of schistosomiasis control.^26^

We previously described a hit series of five dihydrobenz[*e*][1,4]oxazepin-2(3*H*)-one (DHB) compounds with anthelmintic activity against *ex vivo T*. *muris*.^21^ Here, we report our progress in expanding this hit series and understanding the relationship between structure and anthelmintic activity. We also extend our investigations of the activity of the DHB compounds against *Brugia malayi*, a causative agent of lymphatic filariasis, *Heligmosomoides polygyrus bakeri*, a mouse gastrointestinal nematode model, and the human blood fluke, *Schistosoma mansoni*.

## Results

### Novel DHB Chemistry

We recently reported the identification of five DHB hit compounds as a new family of molecules active against *T. muris* adult motility.^21^ Further, one of the compounds **1** (OX02983) was also found to be efficacious at reducing the ability of eggs to establish infection *in vivo*. As we identified a limited number of active DHB family members in the first instance via a library screen, we aimed to investigate the DHB chemotype systematically with the goal of understanding their structure–activity relationships (SARs) and improving potency. Compound **1** (OX02983) was used as a starting point of our investigation.

**Figure 2:**
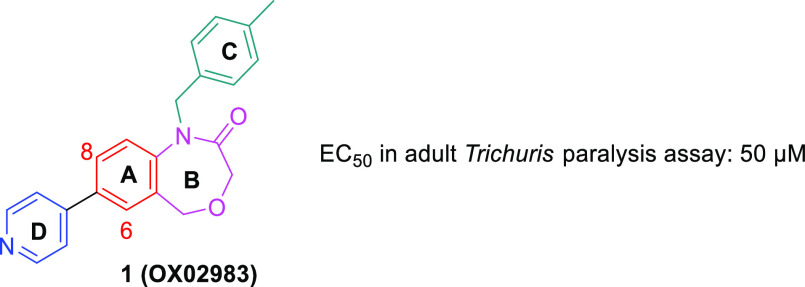
Structure of **1** (OX02983) highlighting the four cycles labeled A–D.

**Scheme 1:**
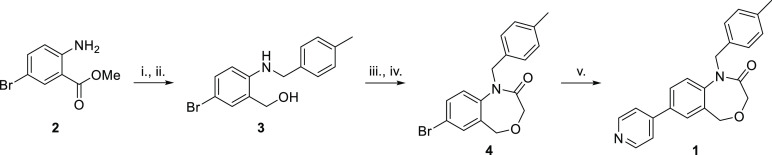
Representative Scheme for Synthesis of DHB Compounds (i) 4-Methylbenzaldehyde (1.5 equiv), AcOH (0.5 equiv), NaBH(OAc)_3_, CH_2_Cl_2_, 0 °C to rt, 48 h; (ii) LiAlH_4_ (1 M in THF, 3.5 equiv); (iii) chloroacetyl chloride (2.0 equiv), NEt_3_ (2.0 equiv), THF, 0 °C to rt, 16 h; (iv) NaOH (10 N, aq.), rt, 2 h; (v) 4-pyridyl-B(OH)_2_ (1.1 equiv), Pd(dppf)Cl_2_ (5 mol %), K_2_CO_3_ (3.0 equiv), 1,4-dioxane/H_2_O (4:1), 90 °C, 18 h.

The synthesis used to prepare DHB **1** was adapted to systematically alter all of the different cycles A–D, as shown in Figure 2. The first step was a reductive amination of the requisite aminobromobenzoate with the desired aldehyde to install cycles A and C. This was followed by a ring-closure step to generate cycle B and finally a cross-coupling reaction to add cycle D (Scheme 1).

It was decided to conduct a systematic SAR investigation and alter the four different cycles within the structure of **1** to understand their importance in the activity against *T. muris* with a view to improving efficacy. As the synthesis is linear, it was logical to investigate from A to D. We therefore started with core B to ascertain the importance of regiochemistry and relative orientation of the substituents (Table 1). All of the prepared compounds were screened using an automated adult *T. muris* motility assay^39^ at 100 μM. Active compounds were also tested at lower concentrations and/or an EC_50_ value determined to assess their relative activity.

**Table 1:**
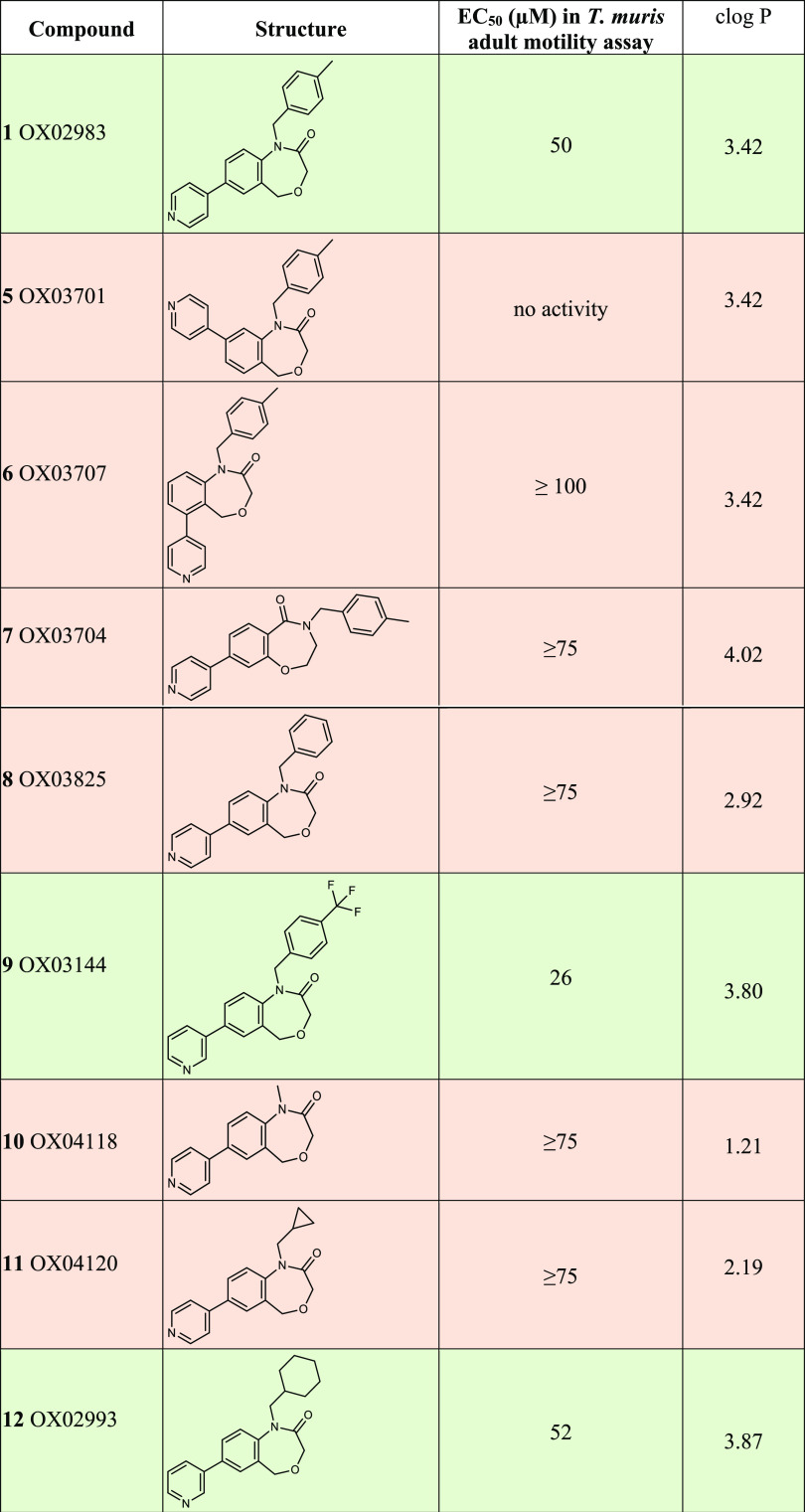

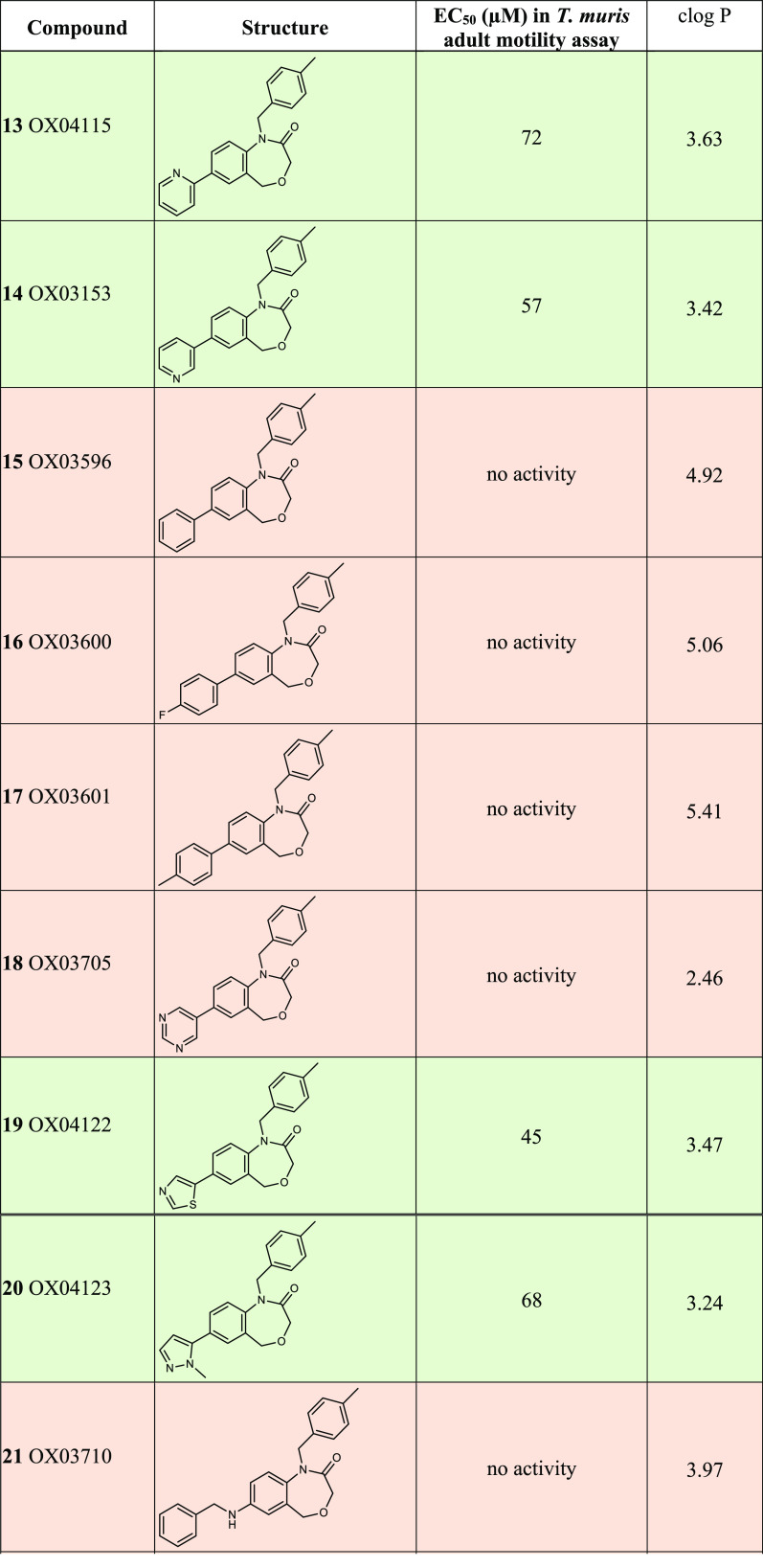

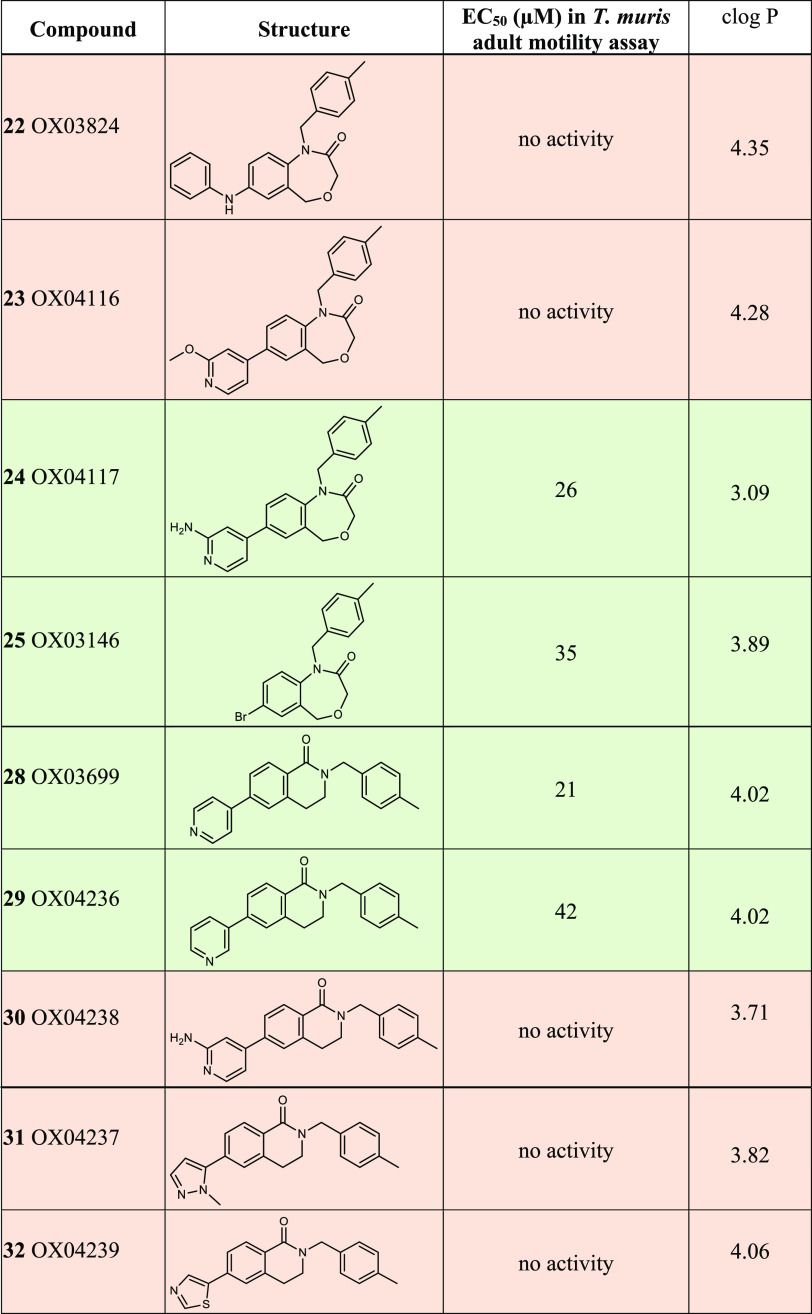
Structures and EC_50_ Values of Representative DHB Compounds in the *T. muris* Adult Motility Assay^a^

a All compounds investigated in this study are described in the Supporting Information File S2. No activity means no clear reduction in motility when tested at 100 μM. Where an EC_50_ estimate is shown, it was calculated using a log-logistic model using the R package drc;^45^ clogP values were calculated using ChemDraw Professional (16.0.0.82 (68)).

Using the appropriate starting materials (see the Supporting Information File 1 for details of the syntheses), the different structural analogues **5**, **6** (where the 4-pyridyl ring is in position 8 and 6 of the bicyclic core respectively, see Figure 2), and **7** (the reverse amide equivalent of **1**) were prepared using a synthesis similar to that of **1** (Table 1). Interestingly, none of the structural analogues exhibited any activity in our *ex vivo* adult *T. muris* motility assay, revealing that the regiochemistry within **1** is important for its activity. The next step was to investigate cycle C; a small set of amines was used in the reductive amination step to prepare analogues **10**, **11**, **12**, **8**, and **9** bearing methyl, cyclopropyl, cyclohexyl, benzyl, and *p*-trifluoromethylbenzyl groups, respectively. From those, only the cyclohexyl substituted derivative **12** and the *p*-trifluoromethylbenzyl substituted derivative **9** showed activity in the motility assay with EC_50_ values of 52 and 26 μM respectively. The next step was to vary cycle D while keeping cycles A–C constant to allow a comparison with **1**. Suzuki reactions were therefore carried out on the 7-bromo precursor with an array of boronic acids and esters. The regioisomers of the pyridyl ring (D) were tolerated with *meta* and *para* giving the best activity. Analogues where the pyridyl ring was replaced with an aryl substituent were all inactive, be they unsubstituted (**15**), substituted with an electron withdrawing group (4-F, **16**), or an electron donating group (4-Me, **17**) (Table 1). Different heterocycles were also trialled in place of the pyridine; a similar level of activity was obtained with the isosteric thiazole (**19**, EC_50_ of 45 μM) and the methylimidazole (**20**, EC_50_ of 68 μM) analogues. Substituting with a pyrimidine (**18**) led to a loss of activity, leading us to hypothesize that the basicity of the substituent may be of importance to the activity. Following this, we prepared phenylamine and benzyl amine-substituted analogues **22** and **21**, but neither exhibited activity against *T. muris*, suggesting that incorporating a linker between cycles A and D was not tolerated. We then turned our interest to substituted pyridyl, and although the methoxy substituted pyridyl (**23**) was not active, the amino pyridyl **24** displayed modestly improved activity compared to **1** (EC_50_ 26 μM), which may be related to its moderately higher basicity.

**Figure 3:**
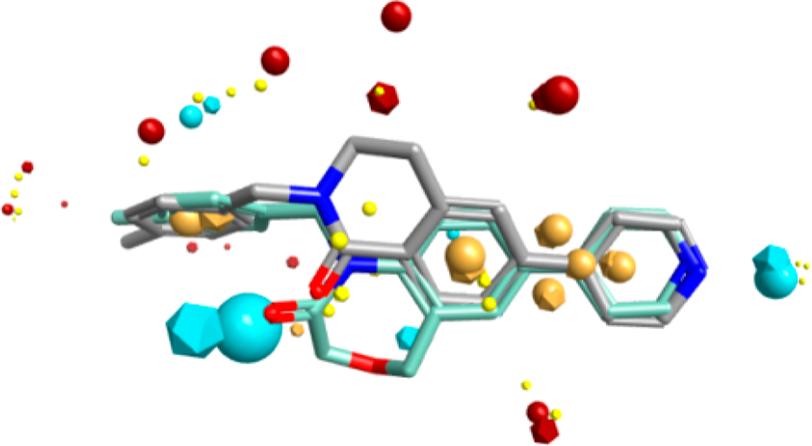
Overlay of **1** (OX02983) (light blue) and **28** (OX3699) (gray); blue spheres represent negative electrostatic field, red spheres represent positive electrostatic field, brown spheres represent hydrophobicity, and small yellow sphere represent the van der Waals force.

In an effort to improve the efficacy further, we looked at more drastic modification to core B by contracting the ring by removing the oxygen atom. Forge (Cresset) was used to overlay **1** and its six-membered ring analogue **28**; a good fit was obtained (∼79% similarity), suggesting dihydrobenzoquinolinones (DBQ) as possible candidates for further improvement (Figure 3).

DBQs have been investigated quite extensively in medicinal chemistry; examples have been reported as antiviral agents through inhibition of HIV replication.^40,41^ Other analogues were found to inhibit WDR5 protein–protein interactions, leading to inhibition of cancer cell proliferation.^42−44^

It was decided to prepare a small number of compounds only using those substituents and comparable regiochemistry that gave the most potent analogues so far.

**Scheme 2:**
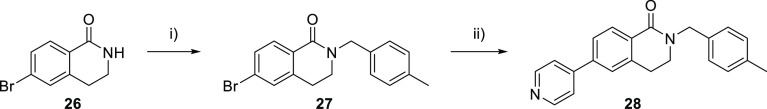
Synthesis of **28** (OX3699) Reagents and conditions: (i) 4-methylbenzyl bromide (2.0 equiv), NaH (1.5 equiv), DMF, rt, 16 h (96%); (ii) 4-pyridyl-B(OH)_2_ (1.1 equiv), Pd(dppf)Cl_2_ (5 mol %), K_2_CO_3_ (3.0 equiv), 1,4-dioxane/H_2_O (4:1), 90 °C 18 h.

The synthesis started with substitution at *N*2 of 6-bromo-3,4-dihydroisoquinolinone **26** with 4-methylbenzyl bromide, followed by a Suzuki coupling reaction with the requisite boronic acid to afford the desired ring contracted **1** mimic, **28** (Scheme 2).

The DBQs bearing the 3-and 4-pyridyl substituents (**28** and **29**) were active in the motility assay and led to similar EC_50_ values to the best results from the DHB series (with EC_50_ values of 21 and 46 μM, respectively). Unfortunately, as soon as we moved away from the simple pyridyl substituent, all activity in the motility assay was lost again. The 2-amino pyrid-5-yl, the best example of ring D in the DHB series, was surprisingly inactive (**30** EC_50_ > 100 μM vs **24** EC_50_ 26 μM). Similarly, the methyl imidazole and the thiazole-substituted analogues (**31** and **32**, respectively) also exhibited no activity in the motility assay, in contrast to their DHB counterparts, suggesting that SARs did not correlate between the DHB and DBQ series. As the best results from the DBQ and the DHB series were largely similar, we felt that this alternative core was not going to substantially enhance the potency of the compounds.

Collectively, these data have improved our understanding or provided insights into the SARs of the DHB/DBQ family of compounds. The structure of cycles A and B in **1** were found to be critical to activity; variations of the toluyl group for ring C generally also led to inactive compounds. Some variations of cycle D were tolerated, and there appeared to be a preference for a basic site within the substituent. However, although we were able to alter the structure resulting in loss of activity, we were unable to improve, only retain, activity.

Apart from the representative compounds presented in Table 1, further similar analogues and all synthetic precursors were prepared and tested (Supporting Information File 2). Together, the results gave us a library of 47 compounds that could then be used against different parasite species to understand whether these compound series showed broad-spectrum anthelmintic activity.

### DHB Compounds Are Active in Models of a Range of Helminth Infections

Development of a new anthelmintic is a long and expensive process, and funding for neglected tropical diseases is limited. Furthermore, multiple parasitic helminths, for example the soil transmitted nematodes *Ascaris*, *Trichuris*, and the human hookworms, the vector transmitted filarial nematodes, and the *Schistosoma* trematodes, are often endemic in the same regions. It would, therefore, be helpful if a new drug would have activity against several target species and worked across the Nematoda and Platyhelminthes phyla. We therefore wanted to investigate whether the DHB series of compounds had a range of activities beyond *Trichuris*.

#### Activity against *B. malayi*

**Figure 4:**
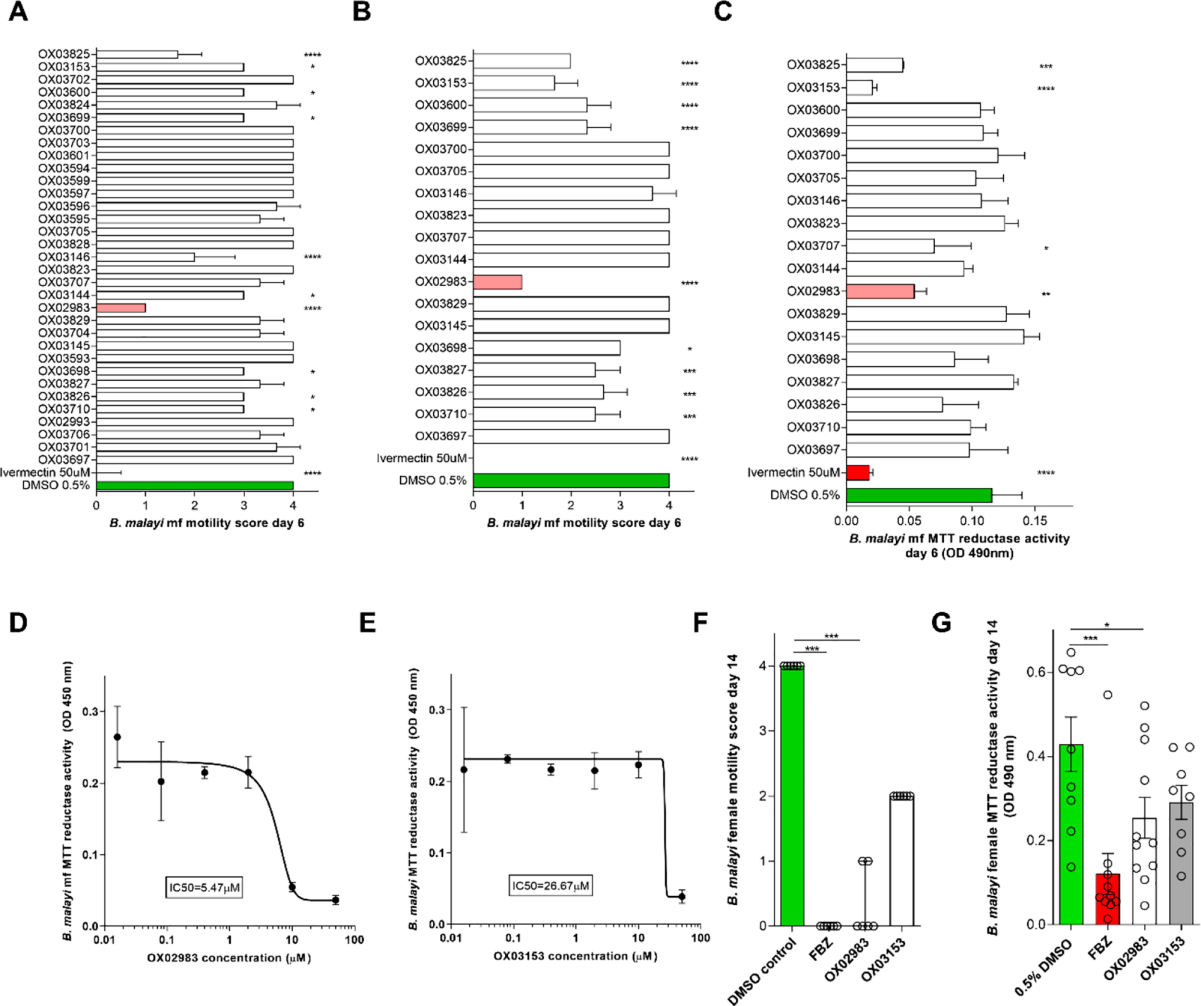
Activity of **33** DHB compounds against *B. malayi* microfilariae and adults. (A) Primary screen: assessment of *B. malayi* mf motility (5 point scoring system) after 6 days of continuous exposure to 35 test compounds screened at 10 μM in triplicate. Ivermectin (50 μM) was the positive control. (B) Confirmatory mf motility and (C) metabolic activity screening of 15 active compounds identified in (A) and 5 inactive compounds (10 μM in triplicate). (D, E) EC_50_ assays of active compounds **1** and **14** on *B. malayi* microfilarial metabolic activity after 6-day continuous exposure. Metabolic activity (C–E) was assessed by colorimetric MTT assay; data are optical density of mf extracts measured at 490 nm. (F) Effects on adult female *B. malayi* motility and (G) metabolic activity following 14 days of continuous exposure to **1** or **14** (10 μM). Flubendazole (10 μM) was used as a positive control in the assay. Data plotted is mean ± SD of 3 replicates (A–E) median and range of 6 replicates (F) and mean ± SEM of 9–11 replicates (G). Significant differences were determined by one-way ANOVA with Holm–Sidak multiple comparisons test (A–C and G) or Kruskal–Wallis with Dunn’s multiple comparisons test (F). Significance is indicated ****P < 0.0001, ***P < 0.001, **P < 0.01, and *P < 0.05.

We first examined single dose efficacy of 33 DHB compounds at 10 μM against the *B. malayi* mf larval stage with motility scored every 24 h. The results after 6 days are shown in Figure 4A. Ivermectin was used as a positive control in the mf assay. **1** showed the most promise in this assay, reducing average motility to a score of 1. From this primary screen, 15 compounds that were determined to significantly impact *B. malayi* mf motility, plus an additional 5 with no discernible effect, were retested in a secondary screen (Figure 4B). These results confirmed the significant reduction in motility caused by 11 compounds and confirmed the paralytic effect of **1**. After 6 days of drug exposure, mf were also tested for metabolic activity, a measure of parasite viability, using the MTT assay (Figure 4C). The MTT assay is part of the WHO-approved *in vitro* assay for antifilarial activity using *O. gutturosa*(46) and has been previously used for assessment of *B. malayi* metabolic status.^47−50^**1** and **14** in particular showed activity in this assay, significantly reducing *B. malayi* mf MTT reductase activity on average by 53 and 82%, respectively (one-way ANOVA with Holm–Sidak’s multiple comparison tests, *P* < 0.01 and *P* < 0.0001). The time course of the effect of **1** against mf motility at different concentrations is shown in Supplementary Figure 3. To determine the dose-dependent efficacy of **1** and **14**, they were tested in a concentration response 6-day experiment (dose range 0.016–50 μM) using MTT reductase activity as a quantitative viability readout (Figure 4D, E). From this, an EC_50_ concentration of 5.5 μM was determined for **1** and 26.7 μM for **14**.

Due to their efficacy against *B. malayi* mf, **1** and **14** were advanced for *in vitro* activity against adult *B. malayi* utilizing a novel, long-term adult worm lymphatic endothelial cell bilayer coculture system. Adult female *B. malayi* exposed to vehicle control retained full survival and motility in culture over 14 days, whereas the positive control, flubendazole (10 μM), mediated complete paralytic activity by day 14 (Kruskal–Wallis with Dunn’s multiple comparisons tests, *P* < 0.001) and significantly reduced metabolic activity by an average of 72% (one-way ANOVA with Holm–Sidak’s multiple comparison tests, *P* < 0.001) (Figure 4F, G). **1** (10 μM) also mediated significant antifilarial activities against adult *B. malayi* by day 14. Motility was completely hindered in 4/6 adult parasites by **1** (Kruskal–Wallis with Dunn’s multiple comparisons tests, *P* < 0.001), while **14** mediated a 50% partial reduction in adult motility. **1** also significantly impacted adult female *B. malayi* metabolic activity, on average by 41% (one-way ANOVA with Holm–Sidak’s multiple comparison tests, *P* < 0.05). Taken together, these results are encouraging because they show that compounds that are active against *T. muris* (a clade I nematode according to the phylogeny of Blaxter) are also active against evolutionarily distant nematodes, as *B. malayi* is a clade III nematode.^51^

#### Activity against *H. polygyrus*

**Figure 5:**
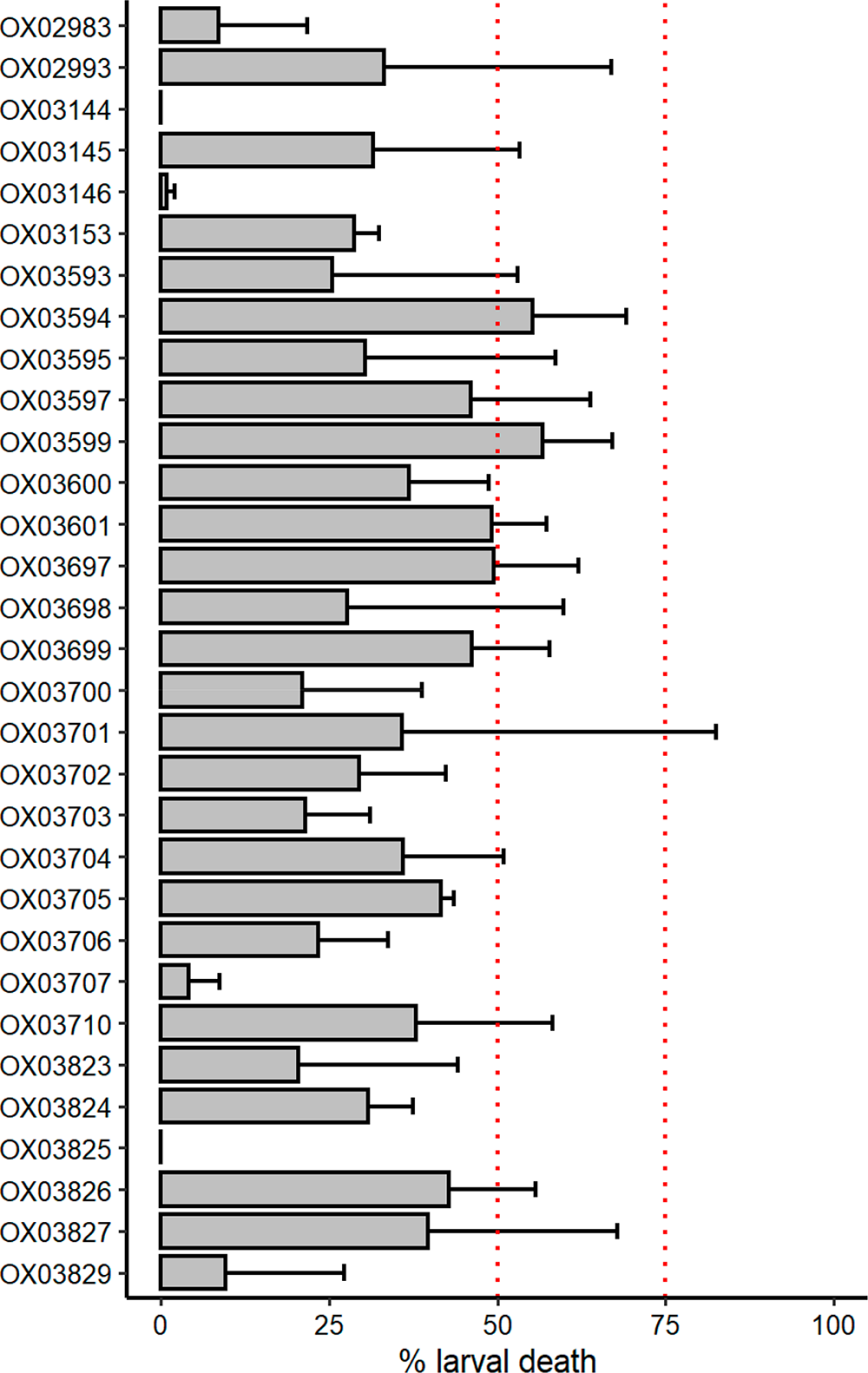
Measurement of the activity of **31** DHB compounds against *H. polygyrus* L3 stage worms. Larval death is measured as the proportion of worms that respond to stimulus. Compounds were tested in duplicate at 100 μM. Dashed lines indicates the cutoff (50%) used to determine hits in this assay and the cutoff for good activity (75%):^20^ <50%, not active; 50–75%, moderate activity; >75%, good; and >90%, excellent activity.

*H. polygyrus bakeri* is an intestinal nematode parasite of laboratory mice.^52^ It is a strongylid nematode related to human hookworm species. Thirty-one DHB compounds were tested at 100 μM against *ex vivo H. polygyrus* L3 stage worms (*n* = 2). The results are shown in Figure 5. The cutoff used to determine hits in this assay is 50% larval death.^20^ Two compounds, **33** and **34**, exceeded this level of larval death and were therefore considered active. They did not, however, reach the threshold for good activity (75%). Given the modest activity of these compounds against *H. polygyrus*, we have not further pursued this direction at this point. Activity of DHB compounds against nematodes in three of the five clades of the phylum Nematoda, according to the phylogeny of Blaxter, supports the potential for development of a pan-nematode control agent from this compound series.^51^

#### Activity against *S. mansoni* Schistosomula

**Figure 6:**
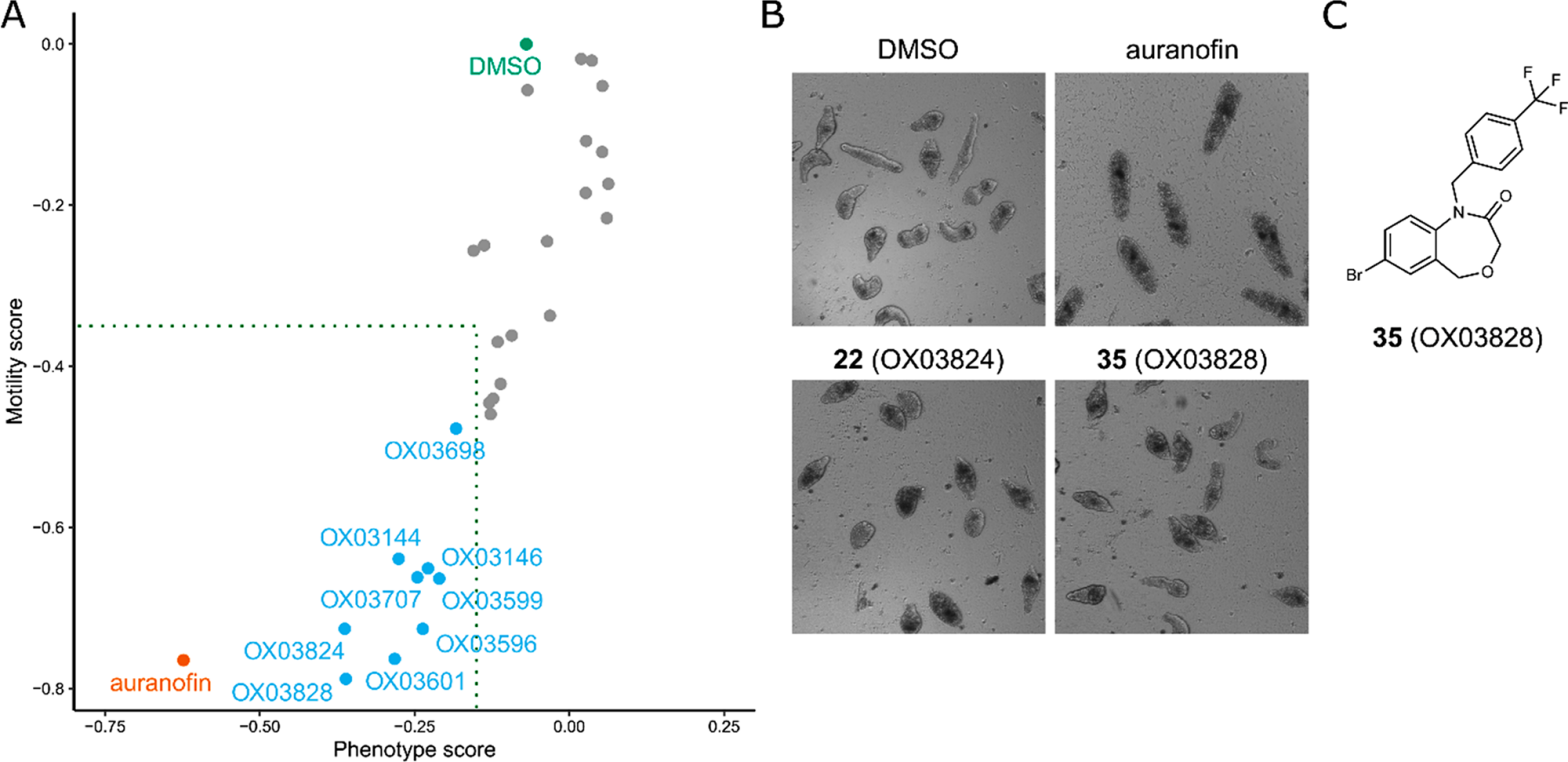
Measurement of the activity of 30 DHB compounds against *S. mansoni* schistosomula using the Roboworm platform. (A) Each point is the measured effect of one compound on the two parameters, motility and phenotype. The phenotype score is calculated by a computational model that assesses morphological and texture properties of the schistosomula.^54^ Compounds were screened at 50 μM. Auranofin was the positive control (screened at 10 μM). Dotted box indicates the threshold for activity in this assay: −0.15 for phenotype and −0.35 for motility; compounds must be below this threshold for both parameters to be considered a hit.^54,56^ Compounds were screened in duplicate on two or three separate occasions, and the data represent the average scores of these experiments. (B) Representative images of schistosomula treated with controls, **22**, and **35**. (C) Structure of **35** (**OX03828**). The structure of **22** (**OX03824**) is shown in Table 1.

Compared to *T. muris*, *H. polygyrus*, and *B. malayi*, which are all parasitic worms within the phylum Nematoda, *S. mansoni* is a more evolutionarily distinct helminth, a trematode within the Platyhelminthes phylum. We screened 30 DHB compounds against *S. mansoni* schistosomula at 50 μM using the Roboworm system. This is an imaging-based screen that measures two parameters, motility and “phenotype”, an assessment of morphological and other features.^54^ Auranofin, an inhibitor of *S. mansoni* thioredoxin glutathione reductase (TGR) activity^55^ was the positive control in this experiment. The results are shown in Figure 6. The cut-offs for defining hit compounds in this assay have been previously defined.^54,56^ Nine compounds were hits for both motility and phenotype measurements. Concentration–response curves were measured for these compounds (Table 2) with EC_50_ values in the range 14–41 μM. It is encouraging that DHB compound series members show activity against such evolutionarily distant pathogens to whipworm, particularly as DHB compounds show little or no cytotoxicity in mammalian cell culture, so these compounds are not broadly toxic.^21^

**Table 2:** EC_50_ Values for Compounds Active in the *S. mansoni* Schistosomula Roboworm Assay^a^

	EC_50_ in *S. mansoni* schistosomula Roboworm assay (μM)	
compound	phenotype	motility
**9** OX03144	28.4	28.0
**25** OX03146	25.5	25.6
**15** OX03596	29.1	26.8
**34** OX03599	14.2	18.3
**17** OX03601	24.4	20.6
**36** OX03698	28.5	26.6
**6** OX03707	32.7	29.3
**22** OX03824	40.9	36.8
**35** OX03828	25.7	20.7

a Compounds were screened at 10, 20, 30, 40, and 50 μM, and EC_50_ values were calculated for each of the screening parameters, phenotype, and motility.

## Discussion

### Investigation of the DHB Structure–Activity Relationship

We previously identified a small hit series of five DHB compounds with activity against *T. muris* adult motility.^21^ In medicinal chemistry, it is important to understand how variations in the structure of the compound affect activity, as this allows us to discover the critical aspects of the compound for target binding with the overall aim of increasing potency as well as improving physicochemical properties. We therefore embarked upon a systematic structure–activity relationship investigation, taking advantage of the convenient synthesis of the DHBs, which allowed us to systematically alter the four cyclic components of this class of compounds. A total of 47 variant compounds were synthesized in this work.

This work has enabled us to define certain essential features of the antiwhipworm DHB compounds. The 4-pyridyl ring (cycle D in Figure 2) must be in the 7 position, unlike the analogues **5** and **6**. The amide moiety of the oxazepinone ring must be as in **1**, and not as in **7**. The oxazepinone nitrogen can be substituted with methylbenzyl, cyclohexyl, and *p-*trifluoromethylbenzyl (**1**, **12**, and **9**), but not methyl, cyclopropyl, or benzyl groups. We also investigated in detail the replacement of cycle B. We found that removal of the oxygen from the DHB core was also consistent with similar activity to **1**: the dihydrobenzoquinolinone compounds **28** and **29** had EC_50_ values of 21 and 42 μM, respectively.

### DHB Compounds as anti-*Trichuris* Agents

We identified the first DHB compounds in a screen of a subset of the library of Chemistry Research Laboratory, University of Oxford small molecule library.^21^ We as yet do not know the target of the DHB/DBQ compounds, and clearly identifying it will be immensely helpful to advance the potency of these compounds. Unfortunately, anthelmintic-resistant mutants or isolates are, as yet, not available in the *T. muris* system. No previous activity that could provide insights into the mechanism of action has to our knowledge been reported for the DHB/DBQ compounds described here.

A detailed pharmacokinetic investigation will also be essential for the future steps of this work. We have reported calculated cLogP values in Table 1, and these molecules conform to the “rule of five”. Interestingly, those compounds retaining anthelmintic activity showed clogP values in the range of approximately 3–4. However, as McKerrow and Lipinski have noted, the rule of 5 is less important for antiparasitic drug development.^57^ This is because antiparasitics, and anthelmintics in particular, have special requirements in terms of permeability and other properties to enable them to reach the target organism and then enter the parasite and reach the target.^58^ An ideal therapeutic against the intestinal nematode parasite *Trichuris trichiura* (found in the cecum) would show minimal gastrointestinal absorption (and rapid hepatic clearance if needed) to maximize exposure in the cecum. Optimization of these parameters, either via the properties of the compound itself or by control of formulation, will be critical to enable the compound to reach the parasite at high concentration *in vivo*.^59^ There is then a further hurdle to optimize: entry into the nematode itself. Structural features that enable compounds to cross the nematode cuticle, which is a barrier to small molecule permeability, are complex.^60,61^ This assumes that oral drugs do reach *Trichuris* by passage to the cecal lumen then crossing the cuticle, which seems to be the case for oxantel and mebendazole but is less clear for albendazole.^62^ Delivery to parasites in the cecum does present particular challenges compared to parasites in the stomach or in the blood, and we hypothesize that lack of optimization of this may be one reason for the lower efficacy of benzimidazoles against *Trichuris.*

### Targeting Multiple Helminth Species with DHB Family Members

Despite being unable to improve efficacy against *T. muris* substantially through structural modifications, we were able to demonstrate activity of our compounds against other helminth parasites. In drug discovery for NTDs, pan-anthelmintic activity is desirable given that polyparasitism in the target population is the norm. Thus, being able to target multiple species of helminths with a single drug administered via mass drug administration programs is of significant benefit. Of particular note was the commonality in DHB compounds active against *T. muris* that were also active against the tissue dwelling nematode parasite, *B. malayi*. The ability of the DHB compounds to act against different clades within the nematode phylum is not unprecedented; indeed, the coadministration of albendazole with ivermectin is currently advocated for control of *Trichuris*, and the same drug combination (in some situations supplemented with diethylcarbamazine) is widely used against lymphatic filariasis.^28^ Indeed, the large-scale efforts to treat lymphatic filariasis have indirectly enhanced the number of people being treated for soil transmitted helminths.^63^ Similarly, the alternative drug combination of albendazole and moxidectin is also being explored for the treatment of trichuriasis given that moxidectin is an approved treatment for onchocerciasis.^64^

In contrast, there are currently no drugs used in MDA that have demonstrated cross-phyla efficacy against both schistosomes and nematodes. Currently, only praziquantel is used for preventative chemotherapy against schistosomes, although coadministration with albendazole is recommended where STHs are coendemic.^65^ Therefore, it was notable that DHB family members could work across phyla, showing some activity against both schistosomes and nematodes. We note that it was unexpected that we found some compounds were active against some nematodes and a trematode, but not against all nematodes. Perhaps this could be due to loss or change in a target during nematode evolution. Differences in drug access between species due to difference in the cuticle or other permeability barriers (i.e., the schistosome’s tegument) may also play a role.

## Conclusions

In this study, we investigated the structure–activity relationship of the DHB compounds, defined essential features for anthelmintic action, and broadened the active series by the discovery of dihydrobenzoquinolinone compounds with activity against *T. muris* adult motility. We have also demonstrated that DHB and related compounds have activity against multiple helminths across different phyla: against the nematodes *B. malayi* and *H. polygyrus* as well as *T. muris* and against the trematode *S. mansoni.* What we have not achieved, however, is the substantive improvement in potency from the 20–50 μM range that would be desirable to progress this series with confidence to *in vivo* testing. Open science, where information is disclosed more freely than in traditional models, is proposed to accelerate drug discovery and make it more cost efficient, especially in the context of neglected diseases.^66,67^ We have therefore decided to report our progress at this point. We note that we do not yet know the target of the DHB/DBQ compounds in helminths. Identifying this target may facilitate the boost in activity for which we are striving.

## Methods

### Ethics Statement

All experimental procedures involving *T. muris* were approved by the University of Manchester Animal Welfare and Ethical Review Board and performed within the guidelines of the Animals (Scientific Procedures) Act, 1986.

All experiments involving *B. malayi* were approved by the ethical committees of the University of Liverpool and Liverpool School of Tropical Medicine (LSTM) and conducted under Home Office Animals (Scientific Procedures) Act 1986 (UK) requirements and the ARRIVE guidelines.

The work on *H. polygyrus* was approved by the local veterinary agency based on Swiss cantonal and national regulations (permission no. 2070).

For experiments involving *S. mansoni*, all procedures performed on mice adhered to the United Kingdom Home Office Animals (Scientific Procedures) Act of 1986 (project licenses PPL 40/3700 and P3B8C46FD) as well as the European Union Animals Directive 2010/63/EU and were approved by Aberystwyth University’s (AU) Animal Welfare and Ethical Review Body (AWERB).

### Chemical Synthesis

Compounds were synthesized from commercially available starting materials and fully characterized by nuclear magnetic resonance spectroscopy and mass spectrometry. Full experimental details and analytical data are provided in the Supporting Information.

### Isolation of *T. muris* Adults

Male and female severe combined immunodeficient (SCID) mice were bred in house at the University of Manchester and used at age 8–12 weeks. Mice were maintained at a temperature of 20–22 °C in a 12 h light, 12h dark lighting schedule in sterile, individually ventilated cages with food and water *ad lib.*

The parasite was maintained and the infectivity of the administered *T. muris* eggs was assessed as previously described.^68,69^ For generation of adult *T. muris* worms, 150 infective eggs were given per oral gavage in water to each SCID mouse. Thirty-five days post infection, mice were sacrificed via schedule one methods. At necropsy, the cecae and colons were removed, opened longitudinally, and washed with prewarmed RPMI-1640 media supplemented with penicillin (500 U/mL) and streptomycin (500 μg/mL). Adult *T. muris* worms were gently removed using fine forceps under a dissecting microscope and maintained at 37 °C in RPMI-1640 media supplemented with penicillin (500 U/mL) and streptomycin (500 μg/mL).

### *T. muris* Adult Motility Assay

Single adult worms were placed in microplate wells containing 100 μL of RPMI-1640 medium, penicillin (500 U/mL), streptomycin (500 μg/mL), and 1 μL (1% v/v) dimethyl sulfoxide (DMSO) or compound dissolved in DMSO. Assay plates were incubated at 37 °C with 5% CO_2_. The INVAPP system was used to quantify worm motility.^39,70^ Movies of the whole plate were recorded (20 frames, 100 ms interval) and motility determined by thresholding the variance of each pixel in the image over time.^71^ Compounds were initially tested at 100 μM. Those showing activity were also tested at lower concentrations, typically 50 and 75 μM, and EC_50_ estimates were measured for compounds of interest using the a log–logistic model and the R package *drc*.^45^

### *B. malayi* Parasite Production

The life cycle of *B. malayi* was maintained in *Aedes aegypti* mosquitoes (Liverpool strain) and inbred Mongolian gerbils housed at the Biomedical Services Unit, University of Liverpool under specific pathogen-free conditions. Microfilariae were harvested from experimentally infected Mongolian gerbils via catheterization under anesthesia and fed to mosquitoes in human blood at 20 000 mf/ml using artificial membranes heated to 37 °C. Mosquitoes were reared for 14 days with daily sugar-water feeding to allow development to larval stage (*Bm*L3). At day 14, *Bm*L3 were collected from infected mosquitoes by stunning at 4 °C, crushing and concentrating using a Baermann’s apparatus and RPMI-1640 media. Male IL-4Rα^–/–^IL-5^–/–^ BALB/c mice (gifted by Prof. Achim Hoerauf, University of Bonn, Germany) aged 6–8 weeks, weighing 18–24 g were infected intraperitoneally with 150 *Bm*L3 and left for 12 weeks to develop to patent adult stage as previously detailed.^72^

### *B. malayi* Microfilaria Assay

*Brugia malayi* microfilariae (mf) were harvested from Mongolian gerbils via intraperitoneal lavage and purified using PD-10 columns (Amersham)_._ Mf densities were then adjusted to 8000/well in complete medium consisting of RPMI-1640 supplemented with 1% penicillin–streptomycin, 1% amphotericin B, and 10% FBS within 96 well plates.

Thirty-three test compounds (10 mM stock in 100% DMSO) were initially tested against mf. Compounds were diluted to 10 μM in complete medium and added to the plated mf. Three replicates were used for each compound, and each plate included ivermectin (50 μM) as a positive control and DMSO (0.5% v/v) as a negative control. Assay plates were incubated for 6 days at 37 °C, 5% CO_2_. Mf were scored daily for motility as a proxy of nematode health using a 5 point scoring system (4 = fully motile, 0 = no motility) as described previously.^73^ Compounds found to reduce motility were progressed to a secondary screen, whereby the MTT assay was employed at day 6 to assess parasite viability quantitatively. For this, excess media was removed from wells, and mf were incubated with 0.5 mg/mL MTT (3-(4,5-dimethylthiazol-2-yl)-2,5-diphenyltetrazolium bromide (Merck) in PBS at 37 °C for 90 min. After washing in PBS and centrifugation, mf pellets were incubated in 100% DMSO for 1 h at 37 °C to solubilize the blue formazan product. Samples were read at OD 490 nm on a 96-well plate reader (Varioskan, Bio-Rad). Compounds exhibiting the greatest activity on parasite viability were progressed further for drug dose titration assays.

### *B. malayi* Adult Assay

Adult female *B. malayi* 12–24 weeks of age were isolated from susceptible IL-4Rα^–/–^IL-5^–/–^ immunodeficient mice, washed in PBS, and added to lymphatic endothelial cell cocultures (HMVECdly; LEC; Lonza) at a density of two parasites per well. Successful test compounds from the mf assay were diluted to 10 μM and added to the trans-wells in 6 mL endothelial basal media with supplements (EGM-2 MV; Lonza). Twelve replicates (*n* = 6 wells) were set up per group with flubendazole (10 μM; Sigma) and DMSO (0.5% v/v) added as controls. Plates were incubated for 14 days at 37 °C, 5% CO_2_ with daily motility scoring, as above. Individual parasites were taken for MTT analysis at day 14.

### *Heligmosomoides polygyrus* Assay

*H. polygyrus* larvae (L3) were obtained by filtering the feces of infected mice and cultivating the eggs on an agar plate for 8–10 days in the dark at 24 °C. Thirty to forty L3 were placed in each well of a 96-well plate for each compound in the presence of 100 μL RPMI 1640 (Gibco, Waltham MA, United States) culture medium supplemented with 5% amphotericin B (250 μg/mL, Sigma-Aldrich, Buchs, Switzerland) and 1% penicillin 10 000 U/mL, and streptomycin 10 mg/mL solution (Sigma-Aldrich, Buchs, Switzerland) with the test drugs (100 μM concentration). Worms were kept at room temperature for 72 h; for evaluation, 50–80 μL of hot water (≈80 °C) was added to each well, and the larvae that responded to this stimulus (the moving worms) were counted. The proportion of larval death was determined. Compounds were tested in duplicate at 100 μM. Control wells were included in each experiment, which included the highest amount of solvent (1% DMSO).

### *S. mansoni* Roboworm Assay

*Biomphalaria glabrata* (NMRI and the previously described pigmented strains^74^) infected previously with *S. mansoni* (Puerto Rican strain) miracidia were exposed for 1.5 h under light at 26 °C. Cercariae were collected and mechanically transformed into schistosomula as previously described.^75^ Mechanically transformed schistosomula were subsequently prepared for high throughput screening (HTS) on the Roboworm platform according to Crusco et al.^76^ All compounds were tested in duplicate during dose response titrations (50, 40, 30, 20, and 10 μM in 0.625% DMSO). Assay controls included 10 μM (in 0.625% DMSO) auranofin (positive control; Sigma-Aldrich, UK) and 0.625% DMSO (negative control). Schistosomula phenotype and motility were quantified after 72 h coculture with compounds as previously described.^54^ Compounds passing both phenotype (−0.15) and motility (−0.35) thresholds were classified as hits. *Z*′ scores for all assays were above 0.35.^77^

## Supporting Information Available

Additional synthetic chemistry information and time course of *Brugia malayi* microfilariae motility scores (PDF)
Summary table of compound structures and assay results (XLSX)
