# Supplementary material for: Structural Requirements for Dihydrobenzoxazepinone Anthelmintics: Actions against Medically Important and Model Parasites: Trichuris muris, Brugia malayi, Heligmosomoides polygyrus, and Schistosoma mansoni
Source: ACS Infect Dis. 2021 Apr 2;7(5):1260–74. doi: 10.1021/acsinfecdis.1c00025 (PMC8154432; doi:10.1021/acsinfecdis.1c00025)
Supplement: Supplementary file 1 — id1c00025_si_001.pdf [file id1c00025_si_001.pdf]

Supporting Information

Structural requirements for dihydrobenzoxazepinone anthelmintics: actions against medically important and model parasites - *Trichuris muris*, *Brugia malayi*, *Heligmosomoides polygyrus* and *Schistosoma mansoni*

Short title: (70 characters) Dihydrobenzoxazepinone efficacy on human and model helminth parasites

Authors:

Frederick A Partridge[1¶], Carole JR Bataille[2¶], Ruth Forman[3¶], Amy E Marriott[4¶], Josephine Forde-Thomas[5¶], Cécile Häberli[6,7¶], Ria L Dinsdale[2], James DB O'Sullivan[3,8], Nicky J Willis[2,9], Graham M Wynne[2], Helen Whiteland[5], John Archer[4], Andrew Steven[4], Jennifer Keiser[6,7], Joseph D Turner[4,10], Karl F Hoffmann[5], Mark J Taylor[4,10], Kathryn J Else[3]*, Angela J Russell[2,11]* and David B Sattelle[1]*

[1]Centre for Respiratory Biology, UCL Respiratory, Division of Medicine, University College London, London, WC1E 6BT, United Kingdom

[2]Department of Chemistry, Chemistry Research Laboratory, University of Oxford, Oxford, OX1 3TA, United Kingdom

[3]Lydia Becker Institute of Immunology and Inflammation, Faculty of Biology, Medicine and Health, University of Manchester, Manchester, M13 9PT, United Kingdom

[4]Centre for Drugs and Diagnostics, Department of Tropical Disease Biology, Liverpool School of Tropical Medicine, Liverpool, L3 5QA, United Kingdom

[5]Institute of Biological, Environmental and Rural Sciences (IBERS), Aberystwyth University, Aberystwyth, Wales, SY23 3DA, United Kingdom.

[6]Department of Medical Parasitology and Infection Biology, Swiss Tropical and Public Health Institute, Socinstrasse 57, Basel, CH-4002, Switzerland.

[7]University of Basel, Petersplatz 1, Basel, CH-4001, Switzerland.

[8]Henry Royce Institute, The University of Manchester, Oxford Road, Manchester, M13 9PL, United Kingdom

[9]Alzheimer's Research UK UCL Drug Discovery Institute, University College London, Gower Street, London, WC1E 6BT, United Kingdom

[10]Centre for Neglected Tropical Diseases, Liverpool School of Tropical Medicine, Liverpool, L3 5QA, United Kingdom.

[11]Department of Pharmacology, University of Oxford, Mansfield Road, Oxford, OX1 3QT, United Kingdom

* Corresponding authors: Email d.sattelle@ucl.ac.uk (DBS), kathryn.else@manchester.ac.uk (KJE), angela.russell@chem.ox.ac.uk (AJR)

¶ These authors contributed equally.

S1 File. Supporting information for synthetic chemistry

General Procedures 3
Experimental data 5
NMR Spectra 24
References 61
Supplementary Figure 3 62

General Procedures

General Procedure 1

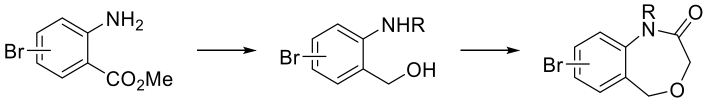

To a solution of the requisite aniline (1.0 eq.) in CH_2Cl_2 (6 mL/mmol) at room temperature was added the desired aldehyde (1.5 eq.) and AcOH (0.5 eq.). The resulting solution was then cooled to 0 °C before addition of $NaBH(OAc)_3$ (2.0 eq.). The mixture was warmed slowly to room temperature and stirred for 16 h before addition of CH_2Cl_2 (5 mL/mmol) and $NaHCO_3$ (sat. aq., 10 mL/mmol), and the aqueous layer extracted with CH_2Cl_2 (5x10 mL/mmol). The combined organic layers were dried (Na_2SO_4) and concentrated *in vacuo* and the crude residue was purified by column chromatography on silica gel to afford the desired methyl ester. A solution of $LiAlH_4$ (1.0 M in THF, 3.5 eq.) was added dropwise over a period of 5-10 min to an ice-cold solution of the requisite methyl ester (1.0 eq.) in THF (7 mL/mmol), and the reaction mixture was stirred for 1 h. NH_4Cl (sat. aq. sol, 3 mL/mmol) was then added and the aqueous layer extracted with EtOAc (3 x 35 mL/mmol), the combined organic layers were dried (Na_2SO_4) and concentrated *in vacuo*. The resulting alcohol was used in the next step without further purification. To an ice-cold solution of desired alcohol in THF (2 mL/mmol) was added Et_3N (2.0 eq.), followed by chloroacetyl chloride (4.4 eq.). The reaction was warmed to room temperature and stirred for 16 h, at which time the crude reaction mixture was passed through a short pad of silica gel, eluted with EtOAc and the solution concentrated *in vacuo*. The crude residue was then dissolved in ^{i}PrOH (2 mL/mmol) before addition of NaOH (10 N, aq., 10 eq.). The reaction was stirred at room temperature for 2 h, diluted with CH_2Cl_2 (5 mL/mmol), washed with brine (ca 5 mL/mmol) and further extracted with CH_2Cl_2 (5 mL/mmol). The combined organic layers were dried (Na_2SO_4), concentrated *in vacuo* and the resulting residue was purified by column chromatography on silica gel.

General Procedure 2

$NaHCO_3$ (1.5 M, aq., 3 eq.) was added to a solution of the bromide (1.0 eq.) and the boronic acid (1.3 eq.) in DMF (3 mL/mmol) in a microwave vial and the vessel was degassed with argon. The requisite Pd catalyst (5 mol%) was then added, the microwave vial was sealed and irradiated at 150 °C for 30 min. The reaction mixture was allowed to cool, diluted with EtOAc (5 mL/mmol), washed with 0.5 M aq. LiCl (3 x 3 mL/mmol). The combined organic layers were dried (Na_2SO_4), then concentrated *in vacuo*. Purification by column chromatography on silica gel (solvents as stated) afforded the desired product.

General Procedure 3

The requisite bromide (600 mg, 2.79 mmol, 1.0 eq.), K_2CO_3 (1.16 g, 8.37 mmol, 3.0 eq.), the requisite boronic acid (572 mg, 3.07 mmol, 1.1 eq.), and Pd(dppf)Cl_2 (100 mg, 0.140 mmol, 5 mol%) were added sequentially to a microwave vial equipped with a magnetic stirrer bar. The reaction vessel was fitted with a rubber septum and purged with N_2 for 5 min, before addition of a degassed solution of 1,4-dioxane/water (5:1, 8 mL) *via* syringe. The vial was then sealed and the reaction heated to 100 °C for 18 h. The mixture was cooled down, diluted with EtOAc (30 mL), and washed with a 50/50 solution of water and brine (2 x 30 mL). The organic phase was dried (Na_2SO_4) and concentrated *in vacuo*. Purification by column chromatography on silica gel (solvents as stated) afforded the desired product.

General Procedure 4 (Coppola et al., 2004)

To a solution of the requisite amide (1 eq.) in DMF (3 mL/mmol) was added the desired aryl bromide (1.3 eq.) followed by NaH (60% in oil, 1.3 eq.) portion-wise, and the resulting solution stirred for 18 h. The reaction was quenched with NH_4Cl (sat. aq. sol.), extracted with EtOAc (3 x 1 mL/mmol), dried (Na_2SO_4), then concentrated *in vacuo*. The compound was purified by flash column chromatography (silica gel).

Experimental data

3700 8-bromo-1-(4-methylbenzyl)-1,5-dihydrobenzo[e][1,4]oxazepin-2(3H)-one

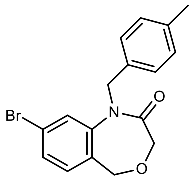

Following **general procedure 1**, (4-bromo-2-((4-methylbenzyl)amino)phenyl)methanol (704 mg, 2.29 mmol) (obtained from the corresponding methyl-2-amino-4-bromobenzoate and benzaldehyde) afforded the title compound **3700** as a white solid (482 mg, 61%), after purification on silica gel (EtOAc:pentane, 1:9); mp = 155-157 ºC (EtOAc); ^{1}H NMR (500 MHz, $CDCl_3$) δ 7.45 (d, *J* = 1.9 Hz, 1H), 7.36 (dd, *J* = 8.1, 1.8 Hz, 1H), 7.16 (d, *J* = 8.1 Hz, 1H), 7.13 (d, *J* = 8.2 Hz, 2H), 7.07 (d, *J* = 8.0 Hz, 2H), 5.04 (s, 2H), 4.45 (s, 2H), 4.04 (s, 2H), 2.29 (s, 3H); ^{13}C NMR (126 MHz, $CDCl_3$) δ 168.3, 143.9, 137.6, 133.7, 131.9, 129.8, 129.5, 128.5, 127.9, 124.8, 123.6, 77.4, 77.4, 77.2, 76.9, 67.6, 67.5, 50.6, 21.2; *m/z* LRMS (ESI$^+$): 370 [M+Na]$^+$; HRMS (ESI$^+$): calc. for $C_{17}H_{17}O_2N^{81}Br$ [M+H]$^+$ 348.0417, found 348.0418.

3702 8-bromo-3,4-dihydrobenzo[f][1,4]oxazepin-5(2H)-one 68 (Zablocki et al., 2016)

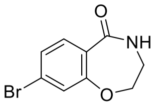

Sodium azide (86 mg, 1.32 mmol, 1.5 eq.) was added over 45 min to an ice-cold solution of 7-bromochroman-4-one (200 mg, 0.881 mmol) in methanesulfonic acid (3 mL) and the resulting solution stirred at RT for 16 h. Conc. HCl (1 mL) was added, and the resulting solid filtered and washed with water, to afford the title compound **3702** as a beige solid (140 mg, 66%); ^{1}H NMR (500 MHz, $CDCl_3$) δ 7.89 (d, *J* = 8.5 Hz, 1H), 7.25 (dd, *J* = 8.5, 1.9 Hz, 2H), 7.21 (d, *J* = 1.9 Hz, 1H), 6.56 (s, 1H), 4.43 – 4.38 (m, 2H), 3.52 (dd, *J* = 9.6, 5.0 Hz, 2H); ^{13}C NMR (126 MHz, $CDCl_3$) δ 169.0, 156.2, 133.8, 127.4, 126.1, 124.3, 121.8, 72.9, 41.9; ; *m/z* LRMS (ESI$^+$): 243 [M+H]$^+$; HRMS (EI): calc for $C_9H_8{}^{81}BrNO_2$ (M^+) 242.9718 found 242.9707.

3703 8-bromo-4-(4-methylbenzyl)-3,4-dihydrobenzo[f][1,4]oxazepin-5(2H)-one

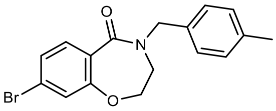

Following **general procedure 4**, amide **3702** (130 mg, 0.537 mmol) and 4-methylbenzyl bromide (199 mg, 1.07 mmol) afforded the title compound as a white solid (137 mg, 74%) after purification on silica gel (EtOAc:pentane, 1:9); mp = 64-66 °C (EtOAc); ^{1}H NMR (500 MHz, $CDCl_3$) δ 7.78 (d, *J* = 8.4 Hz, 1H), 7.30 (dd, *J* = 8.4, 1.9 Hz, 1H), 7.26 – 7.20 (m, 3H), 7.17 (s, 1H), 7.16 (d, *J* = 10.4 Hz, 2H), 4.77 (s, 2H), 4.16 (dd, *J* = 5.5, 4.6 Hz, 2H), 3.47 – 3.42 (m, 2H), 2.34 (s, 3H); ^{13}C NMR (126 MHz, $CDCl_3$) δ 167.8, 154.6, 137.8, 133.8, 132.9, 129.7, 128.5, 126.8, 126.4, 125.9, 124.6, 50.9, 45.9, 21.3; *m/z* LRMS (ESI$^+$): 370 [M+Na]$^+$; HRMS (ESI$^+$): calc. for $C_{17}H_{17}O_2N^{81}Br$ [M+H]$^+$ 348.0417, found 348.0417.

3706 6-bromo-1-(4-methylbenzyl)-1,5-dihydrobenzo[e][1,4]oxazepin-2(3H)-one

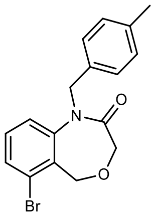

Following **general procedure 1**, (2-bromo-6-((4-methylbenzyl)amino)phenyl)methanol (510 mg, 1.67 mmol) (obtained from the corresponding methyl 2-amino-6-bromobenzoate and 4-methylbenzaldehyde) afforded the title compound **3706** as a white solid (393 mg, 68%) after purification on silica gel (EtOAc:pentane, 1:9); mp = 121 °C (EtOAc); ^{1}H NMR (500 MHz, $CDCl_3$) δ 7.50 (dd, *J* = 6.5, 2.6 Hz, 1H), 7.24 (s, 1H), 7.26 – 7.21 (m, 1H), 7.12 (d, *J* = 8.1 Hz, 2H), 7.07 (d, *J* = 8.0 Hz, 2H), 5.06 (s, 2H), 4.74 (s, 2H), 4.04 (s, 2H), 2.30 (s, 3H); ^{13}C NMR (126 MHz, $CDCl_3$) δ 168.1, 144.1, 137.6, 133.7, 131.2, 130.7, 129.5, 129.4, 128.0, 125.3, 121.1, 77.4, 77.2, 76.9, 50.8, 21.2; *m/z* LRMS (ESI$^+$): 370 [M(^{81}Br)+Na]$^+$; HRMS (ESI$^+$): calc. for $C_{17}H_{17}O_2N^{81}Br$ [M+H]$^+$ 348.0417 found 348.0417.

3827 8-bromo-1-(cyclohexylmethyl)-1,5-dihydrobenzo[e][1,4]oxazepin-2(3H)-one

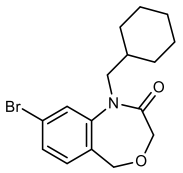

Following **general procedure 1**, ((4-bromo-2-((cyclohexylmethyl)amino)phenyl)methanol (310 mg, 0.863 mmol) (obtained from the corresponding methyl 2-amino-4-bromobenzoate and cyclohexane-carbaldehyde) afforded the title compound **3827** as an off white solid (262 mg, 90%) after purification on silica gel (EtOAc:pentane, 1:4); ^{1}H NMR (400 MHz, CDCl$_3$) δ 7.41 (d, *J* = 1.9 Hz, 1H), 7.39 (dd, *J* = 8.0, 1.8 Hz, 1H), 7.22 (d, *J* = 8.0 Hz, 1H), 4.62 (s, 2H), 3.95 (s, 2H), 3.77 (d, *J* = 6.8 Hz, 2H), 1.73 – 1.54 (m, 8H), 1.14 (td, *J* = 10.7, 9.0, 2.4 Hz, 3H), 1.01 – 0.87 (m, 2H); ^{13}C NMR (101 MHz, CDCl$_3$) δ 168.3, 144.3, 132.2, 129.5, 128.2, 124.4, 123.7, , 67.8, 67.7, 52.9, 36.8, 31.3, 26.3, 25.8; *m/z* LRMS (ESI$^+$): 362 [M+Na]$^+$; HRMS (ESI$^+$): calc. for $C_{16}H_{21}NO_2Br$ [M+H]$^+$ 338.07502, found 338.07519.

3829 1-benzyl-7-bromo-1,5-dihydrobenzo[*e*][1,4]oxazepin-2(3*H*)-one

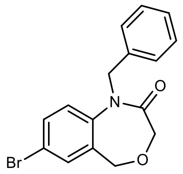

Following **general procedure 1**, (2-(benzylamino)-5-bromophenyl)methanol (940 mg, 3.22 mmol) (obtained from the corresponding methyl 2-amino-5-bromobenzoate and benzaldehyde) afforded the title compound **3829** as a white solid (707 mg, 66%), after purification on silica gel (EtOAc:pentane, 1:9); mp = 115-116 ºC (EtOAc); ^{1}H NMR (500 MHz, MeOD) δ 7.61 (dd, *J* = 8.6, 2.3 Hz, 1H), 7.39 (d, *J* = 8.6 Hz, 1H), 7.30 – 7.18 (m, 5H), 5.14 (s, 3H), 4.44 (s, 2H), 3.99 (s, 2H); ^{13}C NMR (126 MHz, MeOD) δ 170.3, 142.6, 138.2, 134.2, 134.2, 133.1, 129.7, 129.1, 128.8, 124.9, 120.8, 68.3, 68.1, 51.2, 49.5, 49.3, 49.2, 49.0, 48.8, 48.7, 48.5; *m/z* LRMS (ESI$^+$): 356 [M(^{81}Br)+Na]$^+$354 ; HRMS (ESI$^+$): calc. for $C_{16}H_{15}O_2N^{81}Br$ [M+H]$^+$ 334.0260, found 334.0261.

4119 7-bromo-1-(cyclopropylmethyl)-1,5-dihydrobenzo[e][1,4]oxazepin-2(3H)-one

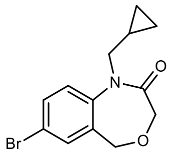

Following **general procedure 1**, (5-bromo-2-((cyclopropylmethyl)amino)phenyl)methanol (280 mg, 0.833 mmol) (obtained from the corresponding methyl 2-amino-5-bromobenzoate and cyclopropane-carbaldehyde) afforded the title compound **4119** as a white solid (262 mg, 90%), after purification on silica gel ((EtOAc:pentane, 1:4); ^{1}H NMR (400 MHz, $CDCl_3$) δ 7.57 (dd, *J* = 8.6, 2.4 Hz, 1H), 7.51 (d, *J* = 2.3 Hz, 1H), 7.18 (d, *J* = 8.6 Hz, 1H), 4.65 (s, 2H), 3.97 (s, 2H), 3.79 (d, *J* = 7.2 Hz, 2H), 1.10 – 0.95 (m, 1H), 0.49 – 0.37 (m, 2H), 0.28 – 0.19 (m, 2H); ^{13}C NMR (101 MHz, $CDCl_3$) δ 167.9, 141.9, 133.4, 133.1, 132.0, 123.5, 119.6, 67.6, 67.4, 51.6, 10.2, 4.0; *m/z* LRMS (ESI$^+$): 296 [M+Na]$^+$; HRMS (ESI$^+$): calc. for $C_{13}H_{15}NO_2Br$ [M+H]$^+$ 296.0286, found 296.0284.

4118a 7-bromo-1-methyl-1,5-dihydrobenzo[e][1,4]oxazepin-2(3H)-one

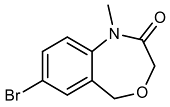

Following **general procedure 4**, bromide **3593** (75 mg, 0.310 mmol) afforded the title compound **4118a** as a white solid (262 mg, 90%), after purification on silica gel (EtOAc:pentane, 1:4); ^{1}H NMR (400 MHz, $CDCl_3$) δ 7.58 (dd, *J* = 8.6, 2.3 Hz, 1H), 7.50 (d, *J* = 2.3 Hz, 1H), 7.12 (d, *J* = 8.6 Hz, 1H), 4.60 (s, 2H), 4.02 (s, 2H), 3.41 (s, 3H); ^{13}C NMR (126 MHz, $CDCl_3$) δ 168.3, 142.9, 133.3, 133.2, 131.0, 122.4, 119.3, 67.8, 67.5, 34.8; ; *m/z* LRMS (ESI$^+$): 255 [M+H]$^+$.

3698 6-bromo-2-(4-methylbenzyl)-3,4-dihydroisoquinolin-1(2H)-one

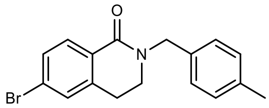

Following **general procedure 4**, 6-bromo-3,4-dihydro-isoquinoline (570 mg, 2.52 mmol) and 4-methylbenzyl bromide (873 mg, 3.28 mmol) afforded the title compound **3698** as a white solid (130 mg, 98%) after purification on silica gel (EtOAc:pentane, 1:4); mp = 97-99 °C (EtOAc); ^{1}H NMR (400

MHz, $CDCl_3$) δ 8.00 (d, *J* = 8.3 Hz, 1H), 7.33 (dd, *J* = 2.0, 1.0 Hz, 1H), 7.21 (d, *J* = 8.0 Hz, 2H), 7.14 (d, *J* = 7.9 Hz, 2H), 4.73 (s, 2H), 3.46 (dd, *J* = 7.1, 6.2 Hz, 2H), 2.90 (t, *J* = 6.6 Hz, 2H), 2.33 (s, 3H); ^{13}C NMR (101 MHz, $CDCl_3$) δ 163.9, 140.0, 137.3, 134.2, 130.4, 130.3, 129.9, 129.4, 128.5, 128.2, 128.2, 126.4, 50.2, 45.1, 27.9, 21.2; *m/z* LRMS (ESI$^+$): 330 [M+H]$^+$; HRMS (EI$^+$): calc. for $C_{17}H_{16}{}^{81}BrNO$ (M^+) requires 331.0395 found 331.0385.

3145 1-(cyclohexylmethyl)-8-(pyridin-3-yl)-1,5-dihydrobenzo[e][1,4]oxazepin-2(3H)-one

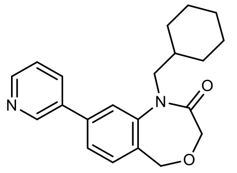

Following **general procedure 3**, bromide **3827** ((400 mg, 1.19 mmol) and 13-pyridyl boronic acid (190 mg, 1.54 mmol) afforded the title product **3145** (398 mg, 99%) as a yellow oil that solidified on standing after purification on silica gel (MeOH:CH_2Cl_2, 5%);^{1}H NMR (400 MHz, $CDCl_3$) δ 8.87 (s, 1H), 8.67 (s, 1H), 7.91 (dt, *J* = 7.9, 1.9 Hz, 1H), 7.52 – 7.41 (m, 3H), 4.73 (s, 2H), 4.02 (s, 2H), 3.88 (d, *J* = 6.9 Hz, 2H), 1.76 – 1.58 (m, 6H), 1.22 – 1.08 (m, 3H), 1.04 – 0.90 (m, 2H); ^{13}C NMR (101 MHz, $CDCl_3$) δ 168.5, 149.3, 148.3, 143.8, 140.0, 135.6, 134.5, 131.6, 129.1, 125.1, 123.8, 119.7, 67.9, 67.9, 53.0, 36.8, 31.3, 26.3, 25.7; *m/z* LRMS (ESI$^+$): 337 [M+H]$^+$; HRMS (ESI$^+$): calc. for $C_{21}H_{25}N_2O_2$ [M+H]$^+$ 337.19105, found 337.19085.

3593 1,5-dihydrobenzo[e][1,4]oxazepin-2(3H)-one

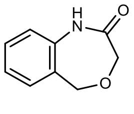

Following **general procedure 1**, title compound **3593** was obtained from *o*-aminobenzyl alcohol (1.00 g, 8.12 mmol) as an orange solid (621 mg, 38%), after purification on silica gel (EtOAc:pentane, 2:3); mp = 98-99 °C (EtOAc); ^{1}H NMR (500 MHz, $CDCl_3$) δ 9.65 (s, 1H), 8.09 (dd, *J* = 8.1, 1.2 Hz, 1H), 7.23 (dd, *J* = 7.6, 1.6 Hz, 1H), 7.15 (td, *J* = 7.5, 1.2 Hz, 1H), 4.76 (s, 2H); ^{13}C NMR (126 MHz, $CDCl_3$) δ 164.9, 136.7, 130.0, 129.4, 129.0, 125.2, 122.5, 64.4, 43.1; *m/z* LRMS (ESI$^+$): 164 [M+H]$^+$; HRMS (ESI$^+$): calc. for $C_9H_{10}NO_2$ [M+H]$^+$ 164.0701, found 164.0706.

3594 1-benzyl-1,5-dihydrobenzo[e][1,4]oxazepin-2(3H)-one

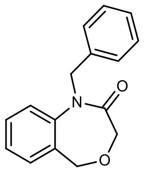

Following **general procedure 4**, amide **3593** (100 mg, 0.612 mmol) and benzyl bromide (145 µL, 1.23 mmol) afforded the title compound as a white solid (43 mg, 28%) after purification on silica gel (EtOAc:pentane, 1:4); mp = 90-92 °C (EtOAc); ^{1}H NMR (500 MHz, $CDCl_3$) δ 7.40 (ddd, *J* = 8.1, 7.3, 1.7 Hz, 1H), 7.26 (s, 0H), 5.12 (s, 2H), 4.52 (s, 2H), 4.06 (s, 2H); ^{13}C NMR (126 MHz, $CDCl_3$) δ 168.6, 142.7, 137.2, 130.7, 130.2, 129.6, 128.8, 127.9, 127.7, 126.8, 121.4, 68.1, 67.7, 50.9; *m/z* LRMS (ESI$^+$): 276 $[M+Na]^+$; HRMS (ESI$^+$): calc. for $C_{16}H_{16}NO_2$ $[M+H]^+$ 254.1175, found 254.1176.

3596 1-(4-methylbenzyl)-7-phenyl-1,5-dihydrobenzo[e][1,4]oxazepin-2(3H)-one

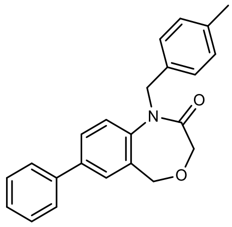

Following **general procedure 2** using $Pd(PPh_3)_4$, bromide **3146** (Partridge et al., 2017) (50 mg, 0.144 mmol), phenylboronic acid (23 mg, 0.187 mmol) afforded the title compound **3596** as yellow solid (14 mg, 28%) after purification on silica gel (EtOAc:pentane, 1:9); mp = 102-105 °C (EtOAc); ^{1}H NMR (500 MHz, $CDCl_3$) δ 7.61 (dd, *J* = 8.4, 2.2 Hz, 1H), 7.59 – 7.53 (m, 2H), 7.51 (d, *J* = 2.2 Hz, 1H), 7.44 (dd, *J* = 8.4, 6.9 Hz, 2H), 7.37 (d, *J* = 7.4 Hz, 1H), 7.34 (d, *J* = 8.3 Hz, 1H), 7.08 (d, *J* = 7.9 Hz, 2H), 5.11 (s, 2H), 4.58 (s, 2H), 4.11 (s, 2H), 2.30 (s, 3H); ^{13}C NMR (126 MHz, $CDCl_3$) δ 168.6, 141.8, 139.7, 139.7, 137.4, 134.2, 129.9, 129.5, 129.2, 129.1, 128.7, 127.9, 127.9, 127.0, 121.8, 77.4, 77.2, 76.9, 68.3, 67.8, 50.6, 21.2; *m/z* LRMS (ESI$^+$): 344 $[M+H]^+$; HRMS (ESI$^+$): calc. for $C_{23}H_{22}NO_2$ $[M+H]^+$ 344.1645, found 344.1643.

3599 7-(4-methoxyphenyl)-1-(4-methylbenzyl)-1,5-dihydrobenzo[e][1,4]oxazepin-2(3H)-one

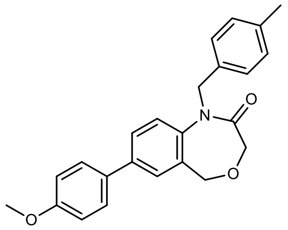

Following **general procedure 2** using $Pd(PPh_3)_4$, bromide **3146** (50 mg, 0.144 mmol) and 4-methoxyphenylboronic acid (29 mg, 0.188 mmol) afforded the title compound **3599** as a yellow solid (11 mg, 21%) after purification on silica gel ((EtOAc:pentane, 1:9); mp = 48-50 °C (EtOAc); ^1H NMR (500 MHz, $CDCl_3$) δ 7.56 (dd, *J* = 8.4, 2.2 Hz, 1H), 7.53 – 7.47 (m, 2H), 7.46 (d, *J* = 2.2 Hz, 1H), 7.31 (d, *J* = 8.4 Hz, 1H), 7.16 (d, *J* = 8.1 Hz, 2H), 7.08 (d, *J* = 7.9 Hz, 2H), 7.02 – 6.94 (m, 2H), 5.10 (s, 2H), 4.56 (s, 2H), 4.10 (s, 2H), 3.85 (s, 3H), 2.29 (s, 3H); ^{13}C NMR (126 MHz, $CDCl_3$) δ 168.6, 159.6, 141.1, 139.3, 137.4, 134.2, 132.2, 129.9, 129.5, 128.7, 128.2, 128.1, 127.9, 121.8, 114.5, 77.4, 77.2, 76.9, 68.4, 67.8, 55.5, 50.6, 21.2; *m/z* LRMS (ESI^+): 396 $[M+Na]^+$; HRMS (ESI^+): calc. for $C_{24}H_{24}NO_3$ $[M+H]^+$ 374.1750, found 374.1749.

3600 7-(4-fluorophenyl)-1-(4-methylbenzyl)-1,5-dihydrobenzo[e][1,4]oxazepin-2(3H)-one

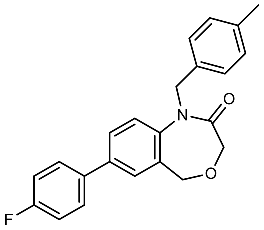

Following **general procedure 2** using $Pd(PPh_3)_4$, bromide **3146** (50 mg, 0.144 mmol) and 4-fluorophenylboronic acid (26 mg, 0.188 mmol)) afforded the title compound **3600** as a yellow solid (14 mg, 27%) after purification by flash column chromatography (EtOAc:pentane, 15%); mp = 127-129 °C (EtOAc); ^1H NMR (500 MHz, $CDCl_3$) δ 7.56 (dd, *J* = 8.4, 2.3 Hz, 1H), 7.56 – 7.48 (m, 2H), 7.46 (d, *J* = 2.2 Hz, 1H), 7.34 (s, 1H), 7.16 (d, *J* = 8.1 Hz, 2H), 7.13 (t, *J* = 8.7 Hz, 2H), 7.08 (d, *J* = 7.9 Hz, 2H), 5.10 (s, 2H), 4.57 (s, 2H), 4.10 (s, 2H), 2.30 (s, 3H); ^{13}C NMR (126 MHz, $CDCl_3$) δ 168.6, 163.8, 161.8, 141.7, 138.7, 137.4, 134.1, 130.0, 129.5, 129.1, 128.7, 128.6, 128.5, 127.9, 121.9, 116.1, 115.9, 77.4, 77.2, 76.9, 68.3, 67.8, 50.6, 21.2; *m/z* LRMS (ESI^+): 385 $[M+Na]^+$; HRMS (ESI^+): calc. for $C_{23}H_{21}FNO_2$ $[M+H]^+$ 362.1550, found 362.1546.

3601 1-(4-methylbenzyl)-7-(p-tolyl)-1,5-dihydrobenzo[e][1,4]oxazepin-2(3H)-one

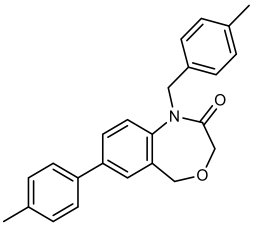

Following **general procedure 2** using $Pd(PPh_3)_4$, bromide **3146** (50 mg, 0.144 mmol) and 4-tolylboronic acid (26 mg, 0.188 mmol) afforded the title compound **3601** as a pale yellow solid (167 mg, 32%) after purification on silica gel (EtOAc:pentane, 15%); mp = 100-101 °C (EtOAc); ^{1}H NMR (500 MHz, $CDCl_3$) δ 7.49 (d, *J* = 2.2 Hz, 1H), 7.46 (d, *J* = 8.2 Hz, 2H), 7.32 (d, *J* = 8.4 Hz, 1H), 7.26 (s, 6H), 7.19 – 7.14 (m, 2H), 7.08 (d, *J* = 7.9 Hz, 2H), 5.10 (s, 2H), 4.57 (s, 2H), 4.10 (s, 2H), 2.39 (s, 3H), 2.30 (s, 3H); ^{13}C NMR (126 MHz, $CDCl_3$) δ 168.6, 141.5, 139.6, 137.8, 137.4, 136.8, 134.2, 129.9, 129.8, 129.5, 128.9, 128.4, 127.9, 126.9, 121.7, 68.4, 67.8, 50.6, 21.3, 21.2; *m/z* LRMS (ESI$^+$): 380 $[M+Na]^+$; HRMS (ESI$^+$): calc. for $C_{24}H_{24}NO_2$ $[M+H]^+$ 358.1801, found 358.1803.

3697 methyl 4-(1-(4-methylbenzyl)-2-oxo-1,2,3,5-tetrahydrobenzo[e][1,4]oxazepin-7-yl)benzoate

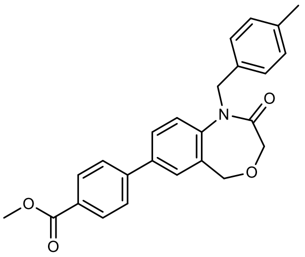

Following **general procedure 2** using $Pd(dppf)Cl_2$, bromide **3146** (200 mg, 0.578 mmol) and 4-methyoxycarbonyl-phenylboronic acid (135 mg, 0.751 mmol), afforded the title compound **3697** as a pale yellow solid (51 mg, 22%) after purification on silica gel (EtOAc:pentane, 3:7); mp = 138-140 °C (EtOAc); ^{1}H NMR (500 MHz, $CDCl_3$) δ 8.11 (d, *J* = 8.4 Hz, 2H), 7.66 (d, *J* = 2.2 Hz, 1H), 7.63 (d, *J* = 8.6 Hz, 2H), 7.37 (d, *J* = 8.4 Hz, 1H), 7.19 – 7.14 (m, 2H), 7.08 (d, *J* = 7.9 Hz, 2H), 5.12 (s, 2H), 4.59 (s, 2H), 4.11 (s, 2H), 3.94 (s, 3H), 2.30 (s, 3H); ^{13}C NMR (126 MHz, $CDCl_3$) δ 168.8, 167.2, 144.3, 142.8, 138.6, 137.7, 134.3, 130.7, 130.4, 129.8, 129.7, 129.7, 129.1, 128.1, 127.2, 122.2, 52.6, 50.8,

21.5.; *m/z* LRMS (ESI$^+$) 424 [M+Na]$^+$; HRMS (ESI$^+$): calc. for $C_{25}H_{24}NO_4$ [M+H]$^+$ 402.1700, found 402.1701.

3701 1-(4-methylbenzyl)-8-(pyridin-4-yl)-1,5-dihydrobenzo[e][1,4]oxazepin-2(3H)-one

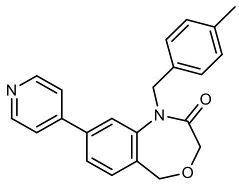

Following **general procedure 2** using Pd(dppf)Cl_2, bromide **3700** (201 mg, 0.578 mmol) and 4-pyridinylboronic acid (106 mg, 0.867 mmol) afforded the title compound **3701** as a brown solid (44 mg, 24%) after purification on silica gel (MeOH:CH_2Cl_2; 2%); mp = 108-110 ºC (MeOH); ^{1}H NMR (500 MHz, $CDCl_3$) δ 8.69 (d, *J* = 6.1 Hz, 1H), 7.50 (d, *J* = 1.7 Hz, 1H), 7.49 (dd, *J* = 7.7, 1.7 Hz, 1H), 7.45 – 7.39 (m, 3H), 7.17 (d, *J* = 7.9 Hz, 2H), 7.08 (d, *J* = 7.8 Hz, 2H), 5.14 (s, 2H), 4.57 (s, 2H), 4.10 (s, 2H), 2.30 (s, 3H); ^{13}C NMR (126 MHz, $CDCl_3$) δ 168.5, 150.7, 147.1, 143.5, 140.3, 137.6, 134.0, 131.5, 130.2, 129.6, 128.0, 125.3, 121.7, 120.1, 67.8, 67.8, 50.7, 21.3.; *m/z* LRMS (ESI$^+$) 345 [M+H]$^+$; HRMS (ESI$^+$): calc. for $C_{22}H_{21}N_2O_2$ [M+H]$^+$ 345.1578, found 345.1597

3704 4-(4-methylbenzyl)-8-(pyridin-4-yl)-3,4-dihydrobenzo[f][1,4]oxazepin-5(2H)-one

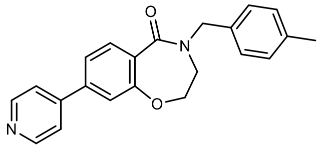

Following **general procedure 2** using Pd(dppf)Cl_2, bromide **3703** (40 mg, 115 mmol) and 4-pyridinylboronic acid (18 mg, 0.150 mmol) afforded the title compound **3704** as a brown solid (37 mg, 93%) after purification on silica gel (MeOH:CH_2Cl_2; 2%); mp = 127-129 ºC (MeOH); ^{1}H NMR (500 MHz, $CDCl_3$) δ 8.72 (s, 1H), 7.57 (s, 1H), 7.45 (dd, *J* = 8.1, 1.8 Hz, 1H), 7.29 – 7.26 (m, 1H), 7.26 (d, *J* = 3.4 Hz, 1H), 7.17 (d, *J* = 7.8 Hz, 1H), 4.81 (s, 1H), 4.22 (t, *J* = 5.1 Hz, 1H), 3.50 (t, *J* = 5.0 Hz, 1H), 2.35 (s, 2H); ^{13}C NMR (126 MHz, $CDCl_3$) δ 168.0, 154.6, 149.6, 142.4, 137.8, 133.8, 132.7, 129.7, 128.5, 127.5, 122.0, 120.1, 73.5, 50.9, 46.0, 21.3; *m/z* LRMS (ESI$^+$) 345 [M+H]$^+$; HRMS (ESI$^+$): calc. for $C_{22}H_{21}N_2O_2$ [M+H]$^+$ 345.1578, found 345.1596.

3705 1-(4-methylbenzyl)-7-(pyrimidin-5-yl)-1,5-dihydrobenzo[*e*][1,4]oxazepin-2(3*H*)-one

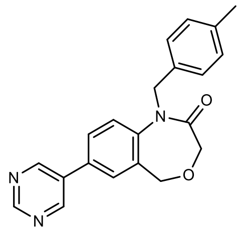

Following **general procedure 2** using Pd(dppf)Cl_2, bromide **3146** (50 mg, 0.144 mmol) and pyrimidine-5-boronic acid (23 mg, 0.188 mmol) afforded the title compound **3705** as a pale yellow solid (15 mg, 31%) after purification on silica gel (EtOAc:pentane, 2:3); mp = 192-194 °C (EtOAc); ^{1}H NMR (500 MHz, $CDCl_3$) δ 9.22 (s, 1H), 8.94 (s, 2H), 7.52 (d, *J* = 2.2 Hz, 1H), 7.44 (d, *J* = 8.3 Hz, 1H), 7.16 (d, *J* = 8.1 Hz, 3H), 7.09 (d, *J* = 7.9 Hz, 2H), 5.13 (s, 2H), 4.60 (s, 2H), 4.12 (s, 2H), 2.30 (s, 3H); ^{13}C NMR (126 MHz, $CDCl_3$) δ 168.4, 158.0, 154.9, 143.4, 137.6, 133.9, 133.1, 132.6, 130.7, 129.6, 129.2, 128.6, 127.9, 122.5, 68.1, 67.9, 50.5, 21.3; *m/z* LRMS (ESI$^+$): 346 [M+H]$^+$; HRMS (ESI$^+$): calc. for $C_{21}H_{20}N_3O_2$ [M+H]$^+$ 346.1550 found 346.1547.

3707 1-(4-methylbenzyl)-6-(pyridin-4-yl)-1,5-dihydrobenzo[e][1,4]oxazepin-2(3H)-one

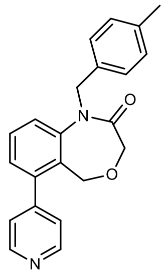

Following **general procedure 2** using Pd(dppf)Cl_2, bromide **3706** (60 mg, 0.173 mmol) and 4-pyridinylboronic acid (32 mg, 0.260 mmol) afforded the title compound **3707** as an orange solid (55 mg, 92%) after purification on silica gel (MeOH:CH_2Cl_2; 2%); mp = 134-136 °C (MeOH); ^{1}H NMR (500 MHz, $CDCl_3$) δ 8.68 (s, 2H), 7.47 (t, *J* = 7.9 Hz, 1H), 7.39 – 7.33 (m, 3H), 7.26 (dd, *J* = 7.6, 1.2 Hz, 1H), 7.07 (d, *J* = 7.8 Hz, 2H), 5.13 (s, 2H), 4.40 (s, 2H), 4.20 (s, 2H), 2.29 (s, 3H); ^{13}C NMR (126 MHz, $CDCl_3$) δ 168.2, 150.1, 147.3, 144.0, 141.2, 137.5, 133.9, 129.9, 129.5, 127.9, 127.7, 127.0,

124.3, 121.5, 67.9, 64.4, 50.8, 21.2; *m/z* LRMS (ESI$^+$) 345 [M+H]$^+$; HRMS (ESI$^+$): calc. for $C_{22}H_{21}N_2O_2$ [M+H]$^+$ requires 345.1598 found 345.1597.

3710 7-(benzylamino)-1-(4-methylbenzyl)-1,5-dihydrobenzo[*e*][1,4]oxazepin-2(3*H*)-one

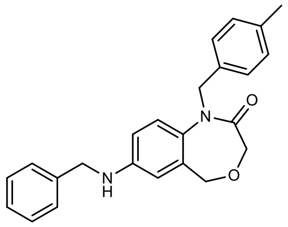

Bromide **3146** (50 mg, 0.144 mmol), benzylamine (19 µL, 0.173 mmol), NaOtBu (28 mg, 0.289 mmol), XPhos (7 mg, 0.014 mmol) and Pd(OAc)$_2$ (2 mg, 0.007 mmol) were added sequentially to a vial and degassed before addition of degassed 1,4-dioxane (3 mL). The vial was sealed and heated to 110 ºC for 16 h. The reaction was cooled down to RT and filtered through celite, using EtOAc as an eluent, the filtrate was then concentrated *in vacuo*. Purification on silica gel (EtOAc:pentane, 3:7) afforded the title compound **3710** as a yellow oil (42 mg, 78%); ^{1}H NMR (500 MHz, CDCl$_3$) δ 7.35 (d, *J* = 3.8 Hz, 4H), 7.32 – 7.27 (m, 1H), 7.13 (d, *J* = 8.0 Hz, 2H), 7.05 (dd, *J* = 8.3, 3.1 Hz, 3H), 6.62 (dd, *J* = 8.7, 2.8 Hz, 1H), 6.51 (d, *J* = 2.7 Hz, 1H), 4.98 (s, 2H), 4.38 (s, 2H), 4.31 (s, 2H), 4.03 (s, 2H), 2.29 (s, 3H); ^{13}C NMR (126 MHz, CDCl$_3$) δ 168.5, 146.8, 138.9, 137.2, 134.5, 132.5, 130.7, 129.3, 128.9, 128.1, 127.6, 127.6, 122.7, 114.0, 113.7, 50.6, 48.5, 21.2; *m/z* LRMS (ESI$^+$) 373 [M+H]$^+$; HRMS (ESI$^+$): calc. for $C_{24}H_{25}N_2O_2$ [M+H]$^+$ requires 373.1911, found 373.1907.

3824 1-(4-methylbenzyl)-7-(phenylamino)-1,5-dihydrobenzo[e][1,4]oxazepin-2(3H)-one

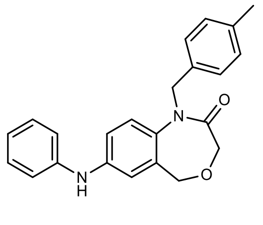

Bromide **3146** (101 mg, 0.289 mmol), aniline (32 µL, 0.347 mmol), NaO^{t}Bu (56 mg, 0.578 mmol), XPhos (14 mg, 0.029 mmol) and Pd(OAc)$_2$ (3 mg, 0.014 mmol) were added sequentially to a vial and degassed before addition of degassed 1,4-dioxane (3 mL). The vial was sealed and heated to 110 ºC for 16 h. The reaction was cooled down to RT and filtered through celite, using EtOAc as an eluent, the

filtrate was then concentrated *in vacuo*. Purification on silica gel (acetone:pentane, 3:7) afforded the title compound **3824** as a brown solid (41 mg, 40%) ; mp= 122-124 ºC (CH_2Cl_2); ^{1}H NMR (500 MHz, $CDCl_3$) δ 7.29 (dd, *J* = 8.5, 7.3 Hz, 2H), 7.15 (dd, *J* = 8.4, 2.1 Hz, 3H), 7.10 – 7.02 (m, 5H), 7.01 – 6.95 (m, 1H), 6.95 (d, *J* = 2.7 Hz, 1H), 5.02 (s, 2H), 4.42 (s, 2H), 4.07 (s, 2H), 2.29 (s, 3H); ^{13}C NMR (126 MHz, $CDCl_3$) δ 168.5, 142.2, 142.2, 137.3, 135.1, 134.4, 130.8, 129.6, 129.4, 128.0, 122.7, 122.1, 118.8, 118.3, 118.2, 77.4, 77.2, 76.9, 50.6, 21.2; *m/z* LRMS (ESI$^+$) 359 [M+H]$^+$; HRMS (ESI$^+$): calc. for $C_{23}H_{23}N_2O_2$ [M+H]$^+$ requires 359.1754, found 359.2904.

3825 1-benzyl-7-(pyridin-4-yl)-1,5-dihydrobenzo[e][1,4]oxazepin-2(3H)-one

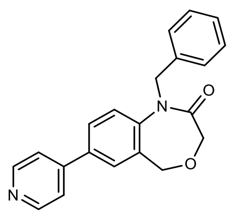

Following **general procedure 2** using Pd(dppf)Cl_2, bromide **3829** (300 mg, 0.908 mmol) and 4-pyridinylboronic acid (144 mg, 1.17 mmol) afforded the title compound **3825** as a brown solid (96 mg, 32%) after purification on silica gel (MeOH:CH_2Cl_2, 2%); mp = 129-130 ºC (MeOH); ^{1}H NMR (500 MHz, MeOD) δ 8.59 (d, *J* = 5.4 Hz, 1H), 7.89 (dd, *J* = 8.4, 2.3 Hz, 1H), 7.80 (d, *J* = 2.3 Hz, 1H), 7.76 – 7.71 (m, 2H), 7.62 (d, *J* = 8.5 Hz, 1H), 7.32 – 7.23 (m, 3H), 5.22 (s, 1H), 4.59 (s, 2H), 4.05 (s, 1H); ^{13}C NMR (126 MHz, MeOD) δ 170.5, 150.7, 149.1, 144.5, 138.3, 137.3, 131.8, 130.1, 129.8, 129.7, 129.0, 128.7, 123.7, 122.0, 68.7, 68.4; *m/z* LRMS (ESI$^+$) 331 [M+H]$^+$; HRMS (ESI$^+$): calc. for $C_{21}H_{19}N_2O_2$+H [M+H]$^+$ requires 331.1441, found 331.1439.

4115 1-(4-methylbenzyl)-7-(pyridin-2-yl)-1,5-dihydrobenzo[e][1,4]oxazepin-2(3H)-one

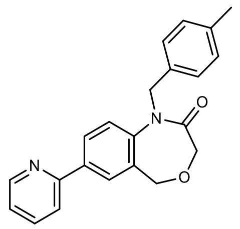

Bromide **3146** (110 mg, 0.328 mmol), 2-pyridyl-MIDA boronate (116 mg, 0.492 mmol), $Cu(OAc)_2$ (33 mg, 0.164 mmol), K_2CO_3 (227 mg, 1.64 mmol), XPhos (10 mg, 0.020 mmol) and $Pd_2(dba)_3$ (6 mg, 0.066 mmol) were added sequentially to a vial and degassed before addition of degassed DMF. The resulting suspension was degassed further for 5 min, the vial was vealed and heated to 100 °C for 16 h. The reaction was cooled down before addition of EtOAc (10 mL) and H_2O:brine (1:1, 30 mL). The organic phase was washed further with H_2O:brine (1:1, 2 x 30 mL), dried (Na_2SO_4), filtered and concentrated *in vacuo*. The residue was purified on silica gel (MeOH:CH_2Cl_2, 3%) to afford the title product **4115** (113 mg, 99%) as a beige solid; ^{1}H NMR (400 MHz, $CDCl_3$) δ 8.67 (ddd, *J* = 4.8, 1.8, 1.0 Hz, 1H), 8.01 (dd, *J* = 8.4, 2.2 Hz, 1H), 7.94 (d, *J* = 2.1 Hz, 1H), 7.79 – 7.70 (m, 1H), 7.69 (dt, *J* = 8.0, 1.2 Hz, 1H), 7.38 (d, *J* = 8.4 Hz, 1H), 7.23 (ddd, *J* = 7.3, 4.8, 1.3 Hz, 1H), 7.14 (d, *J* = 8.1 Hz, 2H), 7.04 (d, *J* = 7.9 Hz, 2H), 5.11 (s, 2H), 4.58 (s, 2H), 4.09 (s, 2H), 2.26 (s, 3H); ^{13}C NMR (101 MHz, $CDCl_3$) δ 168.5, 155.9, 149.9, 143.0, 137.7, 137.3, 137.0, 134.0, 129.9, 129.4, 129.1, 128.4, 127.9, 122.5, 121.7, 120.4, 68.2, 67.7, 50.3; ; *m/z* LRMS (ESI$^+$) 345 $[M+H]^+$; HRMS (ESI$^+$): calc. for $C_{22}H_{21}N_2O_2$ $[M+H]^+$ requires 345.1598 found 345.1599.

4116 7-(2-methoxypyridin-4-yl)-1-(4-methylbenzyl)-1,5-dihydrobenzo[e][1,4]oxazepin-2(3H)-one

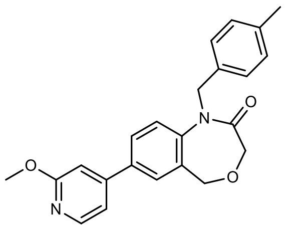

Following **general procedure 3**, **3146** (110 mg, 0.328 mmol) and 2-methoxypyridine boronic acid (55 mg, 0.36 mmol) afforded the title product **4116** (119 mg, 97%) as a light yellow oil that solidified on standing after purification on silica gel (EtOAc:pentane, 2:3); ^{1}H NMR (400 MHz, $CDCl_3$) δ 8.21 (dd, *J* = 5.4, 0.7 Hz, 1H), 7.63 (dd, *J* = 8.4, 2.2 Hz, 1H), 7.54 (d, *J* = 2.2 Hz, 1H), 7.37 (d, *J* = 8.4 Hz, 1H), 7.15 (d, *J* = 8.1 Hz, 2H), 7.08 (d, *J* = 7.8 Hz, 2H), 7.07 (dd, *J* = 5.5, 1.6 Hz, 1H), 6.91 (dd, *J* = 1.6, 0.7 Hz, 1H), 5.11 (s, 2H), 4.58 (s, 2H), 4.10 (s, 2H), 3.98 (s, 3H), 2.29 (s, 3H); ^{13}C NMR (101 MHz, $CDCl_3$) δ 168.5, 165.1, 149.7, 147.5, 143.3, 137.5, 136.5, 134.0, 130.2, 129.5, 129.2, 128.6, 127.8, 122.0, 115.1,

108.4, 68.2, 67.9, 53.8, 50.5, 21.2; *m/z* LRMS (ESI$^+$) 375 [M+H]$^+$; HRMS (ESI$^+$): calc. for $C_{23}H_{23}N_2O_2$ [M+H]$^+$ requires 375.1709 found 375.1709.

4117 7-(2-aminopyridin-4-yl)-1-(4-methylbenzyl)-1,5-dihydrobenzo[e][1,4]oxazepin-2(3H)-one

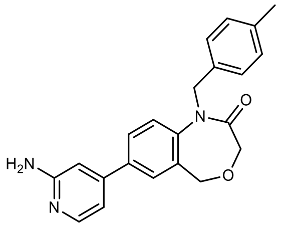

Following **general procedure 3**, **3146** (110 mg, 0.328 mmol) and 2-aminopyridine-4-boronic pinacol ester (79 mg, 0.361 mmol) afforded the title product **4117** (113 mg, 96%) as a light yellow oil that solidified on standing after purification on silica gel (MeOH:CH_2Cl_2, 4%); ^{1}H NMR (400 MHz, MeOD) δ 7.92 (dd, *J* = 5.5, 0.8 Hz, 1H), 7.71 (dd, *J* = 8.4, 2.2 Hz, 1H), 7.60 (d, *J* = 2.2 Hz, 1H), 7.52 (d, *J* = 8.4 Hz, 1H), 7.13 (d, *J* = 8.2 Hz, 2H), 7.04 (dd, *J* = 8.4, 0.8 Hz, 2H), 6.83 (dd, *J* = 5.5, 1.7 Hz, 1H), 6.80 (dd, *J* = 1.6, 0.8 Hz, 1H), 5.47 (s, 2H), 5.12 (s, 2H), 4.86 (s, 4H), 4.51 (s, 2H), 3.99 (s, 2H), 2.23 (s, 3H); ^{13}C NMR (101 MHz, MeOD) δ 170.4, 161.4, 150.2, 148.6, 143.9, 138.6, 138.4, 135.3, 131.5, 130.2, 130.2, 129.8, 129.5, 129.0, 123.3, 112.1, 107.4, 68.7, 68.4, 50.8, 25.0; *m/z* LRMS (ESI$^+$) 360 [M+H]$^+$; HRMS (ESI$^+$): calc. for $C_{22}H_{22}N_3O_2$ [M+H]$^+$ requires 360.1712 found 360.1711.

4118 1-methyl-7-(pyridin-4-yl)-1,5-dihydrobenzo[e][1,4]oxazepin-2(3H)-one

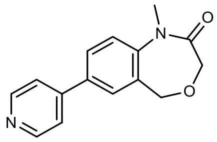

Following **general procedure 3**, 7-bromo-1-methyl-1,5-dihydrobenzo[e][1,4]oxazepin-3(2H)-one (30 mg, 0.118 mmol) and 4-pyridyl boronic acid hydrate (16 mg, 0.129 mmol) afforded the title product **4118** (22 mg, 74%) as a beige solid after purification on silica gel (MeOH:CH_2Cl_2, 2%); ^{1}H NMR (400 MHz, $CDCl_3$) δ 8.72 – 8.66 (m, 2H), 7.74 (dd, *J* = 8.4, 2.2 Hz, 1H), 7.63 (d, *J* = 2.2 Hz, 1H), 7.56 – 7.46 (m, 2H), 7.36 (d, *J* = 8.4 Hz, 1H), 4.73 (s, 2H), 4.08 (s, 2H), 3.48 (s, 3H) ; ^{13}C NMR (126 MHz,

CDCl$_3$) δ 168.5, 150.5, 147.0, 144.6, 136.1, 129.8, 129.1, 128.7, 121.5, 121.4, 68.1, 67.8, 34.8; *m/z* LRMS (ESI$^+$) 255 [M+H]$^+$; HRMS (ESI$^+$): calc. for $C_{15}H_{15}N_2O_2$ [M+H]$^+$ requires 255.1134 found 255.1135.

4120 1-(cyclopropylmethyl)-7-(pyridin-4-yl)-1,5-dihydrobenzo[e][1,4]oxazepin-2(3H)-one

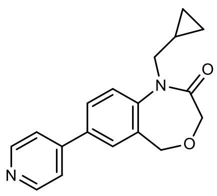

Following **general procedure 3**, **4119** (134 mg, 0.453 mmol) and 4-pyridyl boronic acid hydrate (61 mg, 0.498 mmol) afforded the title product **4120** (130 mg, 97%) as an orange oil that solidified on standing after purification on silica gel (MeOH:CH_2Cl_2, 4%); ^{1}H NMR (500 MHz, DMSO) δ 8.69 (s, 2H), 8.01 (d, *J* = 2.2 Hz, 1H), 7.98 (dd, *J* = 8.4, 2.3 Hz, 1H), 7.79 (d, *J* = 4.6 Hz, 2H), 7.66 (d, *J* = 8.4 Hz, 1H), 4.72 (s, 2H), 3.86 (s, 2H), 3.84 (d, *J* = 7.2 Hz, 2H), 0.98 (tt, *J* = 7.6, 4.7 Hz, 1H), 0.42 – 0.32 (m, 2H), 0.22 – 0.15 (m, 2H); ^{13}C NMR (126 MHz, DMSO) δ 167.3, 150.3, 145.6, 143.3, 134.8, 130.1, 128.7, 128.4, 122.7, 67.0, 66.8, 50.1, 10.0, 3.5; *m/z* LRMS (ESI$^+$) 295 [M+H]$^+$; HRMS (ESI$^+$): calc. for $C_{18}H_{19}N_2O_2$ [M+H]$^+$ requires 295.1447 found 295.1448.

4122 1-(4-methylbenzyl)-7-(thiazol-5-yl)-1,5-dihydrobenzo[e][1,4]oxazepin-2(3H)-one

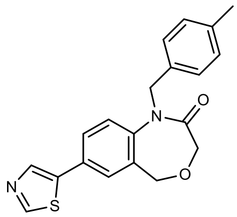

Following **general procedure 3**, **3146** (100 mg, 0.290 mmol) and thiazole-5-boronic pinacol ester (73 mg, 0.348 mmol) afforded the title product **4122** (98 mg, 97%) as an off white solid purification on silica gel (MeOH:CH_2Cl_2, 4%); ^{1}H NMR (400 MHz, MeOD) δ 8.96 (s, 1H), 8.18 (s, 1H), 7.74 (dd, *J* = 8.4, 2.3 Hz, 1H), 7.65 (d, *J* = 2.3 Hz, 1H), 7.53 (d, *J* = 8.5 Hz, 1H), 7.15 (d, *J* = 8.0 Hz, 2H), 7.06 (d, *J* = 8.2 Hz, 2H), 5.13 (s, 2H), 4.51 (s, 2H), 4.01 (s, 2H), 2.24 (s, 3H); ^{13}C NMR (101 MHz, MeOD) δ

170.4, 154.9, 143.4, 140.2, 139.6, 138.6, 135.3, 131.9, 130.9, 130.3, 129.6, 129.5, 129.1, 129.1, 123.9, 68.5, 68.4, 25.0; *m/z* LRMS (ESI$^+$) 351 [M+H]$^+$; HRMS (ESI$^+$): calc. for $C_{20}H_{19}N_2O_2S$ [M+H]$^+$ requires 351.1167 found 351.1166

4123 7-(1-methyl-1H-pyrazol-5-yl)-1-(4-methylbenzyl)-1,5-dihydrobenzo[e][1,4]oxazepin-2(3H)-one

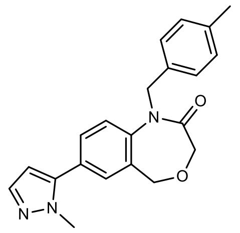

Following **general procedure 3**, **3146** (100 mg, 0.290 mmol) and 1-methylpyrazole-5- boronic acid (72 mg, 0.348 mmol) afforded the title product **4237** (96 mg, 95%) as a light orange oil that solidified on standing after purification on silica gel (MeOH:CH_2Cl_2, 4%); ^{1}H NMR (400 MHz, MeOD) δ 7.57 (d, *J* = 1.3 Hz, 2H), 7.47 (d, *J* = 2.0 Hz, 1H), 7.46 (t, *J* = 1.3 Hz, 1H), 7.15 (d, *J* = 8.1 Hz, 2H), 7.05 (d, *J* = 7.4 Hz, 1H), 6.36 (d, *J* = 2.0 Hz, 1H), 5.14 (s, 1H), 4.88 (s, 1H), 4.52 (s, 2H), 4.01 (s, 2H), 3.84 (s, 3H), 2.23 (s, 3H); ^{13}C NMR (101 MHz, MeOD) δ 170.4, 143.9, 143.6, 139.4, 138.6, 135.3, 131.6, 131.4, 130.3, 130.2, 129.0, 123.4, 107.4, 68.5, 68.4, 37.7, 21.1; *m/z* LRMS (ESI$^+$) 348 [M+H]$^+$; HRMS (ESI$^+$): calc. for $C_{21}H_{22}N_3O_2$ [M+H]$^+$ requires 348.1712 found 348.1713

3699 2-(4-methylbenzyl)-6-(pyridin-4-yl)-3,4-dihydroisoquinolin-1(2H)-one

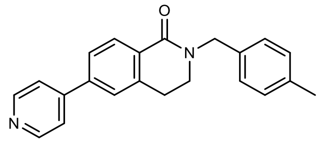

Following **general procedure 2** using Pd(dppf)Cl_2, compound **3698** (50 mg, 0.151 mmol) and 4-pyridinylboronic acid (24 mg, 0.197 mmol), afforded the title compound as a brown solid (48 mg, 96%) after purification by flash column chromatography (MeOH:CH_2Cl_2, 5%); mp = 151-152 ºC (MeOH); ^{1}H NMR (500 MHz, $CDCl_3$) δ 8.69 (d, *J* = 4.7 Hz, 2H), 8.26 (d, *J* = 8.0 Hz, 1H), 7.62 (dd, *J* = 8.1, 1.9 Hz, 1H), 7.52 (d, *J* = 6.1 Hz, 1H), 7.42 (d, *J* = 1.8 Hz, 1H), 7.24 (d, *J* = 8.1 Hz, 2H), 7.15 (d, *J* = 7.9

Hz, 2H), 4.78 (s, 2H), 3.52 (t, *J* = 6.6 Hz, 2H), 3.01 (t, *J* = 6.6 Hz, 2H), 2.34 (s, 3H); ^{13}C NMR (126 MHz, $CDCl_3$) δ 164.1, 150.5, 147.5, 141.4, 139.0, 137.4, 134.3, 130.1, 129.5, 128.3, 125.9, 125.7, 121.8, 50.3, 45.3, 28.4, 21.3; *m/z* LRMS (ESI$^+$) 329 $[M+H]^+$; HRMS (ESI$^+$): calc. for $C_{22}H_{21}N_3O$ $[M+H]^+$ requires 329.1648 found 329.1646.

4236 2-(4-methylbenzyl)-6-(pyridin-3-yl)-3,4-dihydroisoquinolin-1(2H)-one

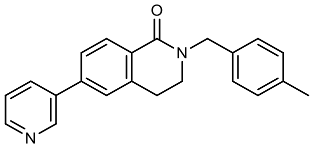

Following **general procedure 3**, **3698** (80 mg, 0.243 mmol) and 3-pyridyl boronic acid (33 mg, 0.267 mmol) afforded the title product **4236** (76 mg, 99%) as a brown oil that solidified on standing after purification on silica gel (MeOH:CH_2Cl_2, 4%); ^{1}H NMR (400 MHz, Acetone) δ 8.92 (d, *J* = 2.6 Hz, 1H), 8.60 (dd, *J* = 4.9, 1.6 Hz, 1H), 8.15 (d, *J* = 8.1 Hz, 1H), 8.11 – 8.03 (m, 1H), 7.71 (dd, *J* = 8.1, 1.9 Hz, 1H), 7.62 (t, *J* = 1.2 Hz, 1H), 7.48 (dd, *J* = 8.1, 4.7 Hz, 1H), 7.28 (d, *J* = 7.9 Hz, 2H), 7.17 (d, *J* = 7.9 Hz, 2H), 4.76 (s, 2H), 3.57 (dd, *J* = 7.1, 6.2 Hz, 2H), 3.08 (t, *J* = 6.6 Hz, 2H), 2.31 (s, 3H); ^{13}C NMR (101 MHz, $CDCl_3$) δ 164.3, 148.9, 148.1, 141.0, 139.0, 137.3, 136.0, 134.8, 134.4, 132.2, 129.5, 129.3, 128.2, 128.2, 126.0, 125.7, 123.9, 50.3, 45.3, 28.3, 21.2; *m/z* LRMS (ESI$^+$) 329 $[M+H]^+$; HRMS (ESI$^+$): calc. for $C_{22}H_{21}N_3O$ $[M+H]^+$ requires 329.1648 found 329.1647.

4237 6-(1-methyl-1H-pyrazol-5-yl)-2-(4-methylbenzyl)-3,4-dihydroisoquinolin-1(2H)-one

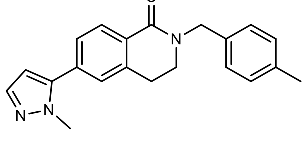

Following **general procedure 3**, **3698** (80 mg, 0.243 mmol) and 1-methylpyrazole-5- boronic acid (34 mg, 0.267 mmol) afforded the title product **4237** (71 mg, 88%) as a brown oil after purification on silica gel (MeOH:CH_2Cl_2, 6%); ^{1}H NMR (400 MHz, MeOD) δ 8.07 (d, *J* = 8.0 Hz, 1H), 7.48 (dd, *J* = 2.0, 1.1 Hz, 1H), 7.44 (dd, *J* = 8.0, 1.7 Hz, 1H), 7.32 (t, *J* = 1.3 Hz, 1H), 7.19 (d, *J* = 7.8 Hz, 2H), 7.10 (d, *J* =

7.7 Hz, 2H), 6.39 (dd, *J* = 2.1, 1.0 Hz, 1H), 4.70 (s, 2H), 3.85 (d, *J* = 1.1 Hz, 3H), 3.51 – 3.43 (m, 2H), 2.94 (t, *J* = 6.6 Hz, 2H), 2.26 (s, 3H) ^{13}C NMR (101 MHz, MeOD) δ 165.7, 144.2, 140.6, 139.5, 138.4, 135.3, 135.1, 130.3, 130.1, 129.4, 129.0, 128.5, 128.2, 107.6, 46.5, 37.9, 28.7, 21.2; *m/z* LRMS (ESI$^+$) 332 [M+H]$^+$; HRMS (ESI$^+$): calc. for $C_{21}H_{22}N_3O$ [M+H]$^+$ requires 332.1763 found 332.1762.

4238 6-(2-aminopyridin-4-yl)-2-(4-methylbenzyl)-3,4-dihydroisoquinolin-1(2H)-one

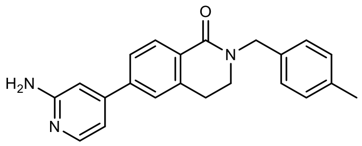

Following **general procedure 3**, **3698** (80 mg, 0.243 mmol) and 2-amino-pyridine-4-boronic pinacol ester (59 mg, 0.267 mmol) afforded the title product **4238** (79 mg, 95%) as a brown oil that solidified on standing after purification on silica gel (MeOH:CH_2Cl_2, 8%); ^{1}H NMR (600 MHz, DMSO) δ 8.01 (d, *J* = 8.1 Hz, 1H), 7.99 (d, *J* = 5.0 Hz, 1H), 7.62 (dd, *J* = 8.1, 1.8 Hz, 1H), 7.55 (d, *J* = 1.9 Hz, 1H), 7.21 (d, *J* = 8.0 Hz, 2H), 7.15 (d, *J* = 7.9 Hz, 2H), 6.81 (dd, *J* = 5.4, 1.7 Hz, 1H), 6.74 (dd, *J* = 1.6, 0.8 Hz, 1H), 6.01 (s, 2H), 4.68 (s, 2H), 3.49 (t, *J* = 6.6 Hz, 2H), 3.01 (t, *J* = 6.6 Hz, 2H), 2.28 (s, 3H); ^{13}C NMR (151 MHz, DMSO) δ 163.0, 160.4, 148.5, 147.3, 141.4, 139.4, 136.3, 134.6, 129.1, 128.3, 127.6, 125.4, 124.8, 110.0, 105.2, 49.4, 45.1, 27.4, 20.7; *m/z* LRMS (ESI$^+$) 344 [M+H]$^+$; HRMS (ESI$^+$): calc. for $C_{22}H_{22}N_3O$ [M+H]$^+$ requires 344.1763 found 344.1764.

4239 2-(4-methylbenzyl)-6-(thiazol-5-yl)-3,4-dihydroisoquinolin-1(2H)-one

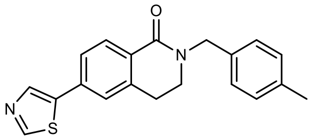

Following **general procedure 3**, **3698** (80 mg, 0.243 mmol) and thiazole boronic acid (56 mg, 0.267 mmol) afforded the title product **4239** (78 mg, 97%) as a light orange oil that solidified on standing after purification on silica gel (MeOH:CH_2Cl_2, 6%); ^{1}H NMR (400 MHz, $CDCl_3$) δ 8.82 (s, 1H), 8.19 (d, *J* = 8.1 Hz, 1H), 8.16 (s, 1H), 7.57 (dd, *J* = 8.0, 1.6 Hz, 1H), 7.37 (s, 1H), 7.24 (d, *J* = 14.2 Hz, 4H), 7.15 (d, *J* = 7.8 Hz, 2H), 4.76 (s, 2H), 3.50 (dd, *J* = 8.4, 4.8 Hz, 2H), 2.97 (t, *J* = 6.6 Hz, 2H), 2.34 (s,

3H); ^{13}C NMR (126 MHz, $CDCl_3$) δ 164.0, 139.2, 137.4, 134.3, 129.6, 129.6, 129.5, 129.5, 129.5, 128.3, 125.8, 125.5, 50.4, 45.2, 28.3, 21.3; *m/z* LRMS (ESI$^+$) 335 $[M+H]^+$; HRMS (ESI$^+$): calc. for $C_{20}H_{19}N_2OS$ $[M+H]^+$ requires 335.1218 found 335.1218.

NMR Spectra

3700

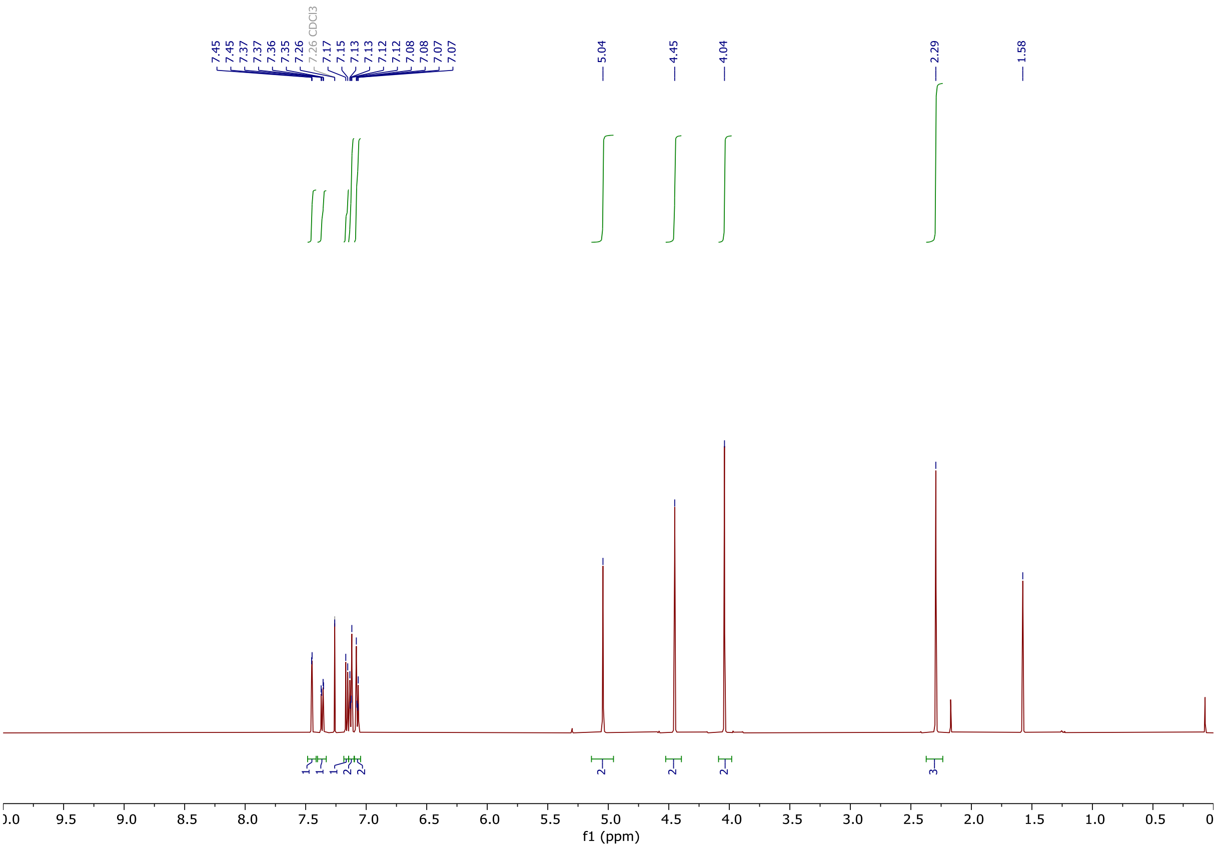

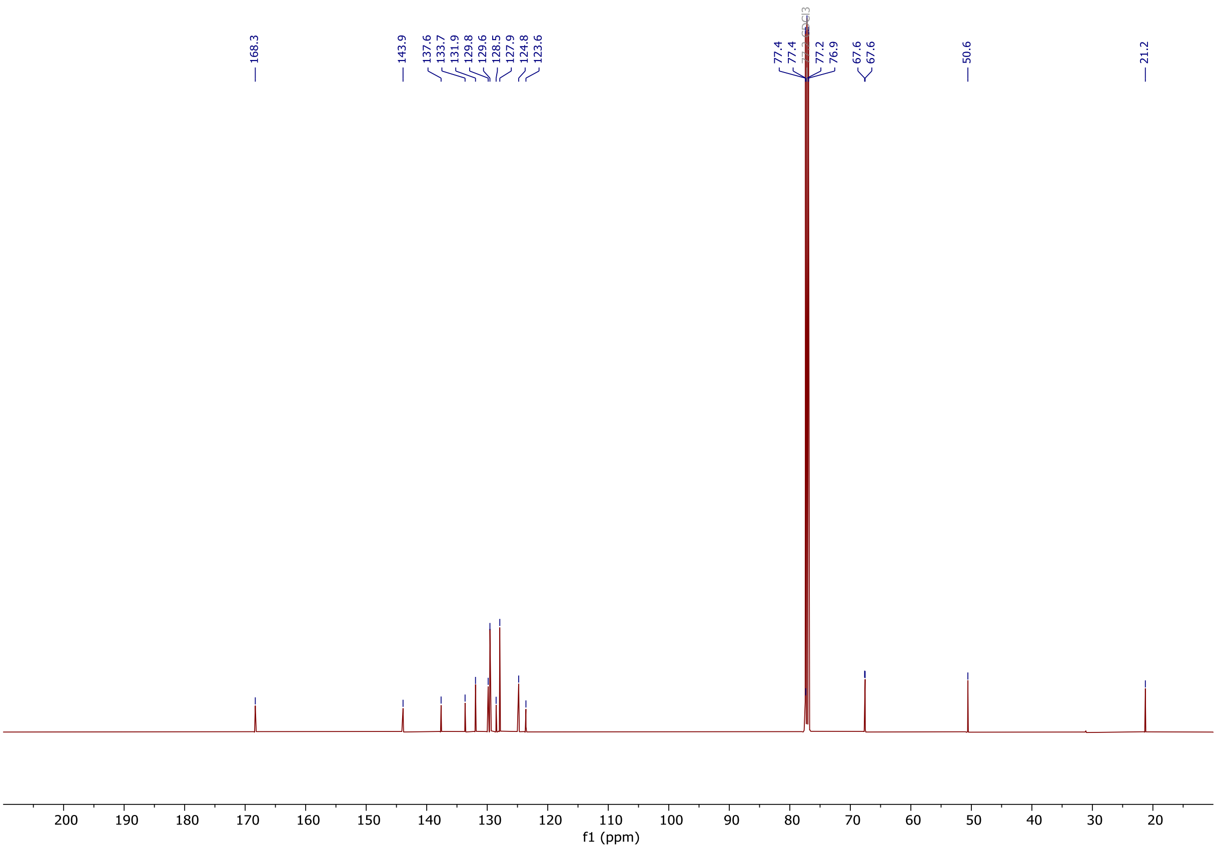

168.3
143.9
137.6
133.7
131.9
129.8
129.6
128.5
127.9
124.8
123.6
77.4
77.4
77.2 CDCl3
77.2
76.9
67.6
67.6
50.6
21.2
f1 (ppm)

3702

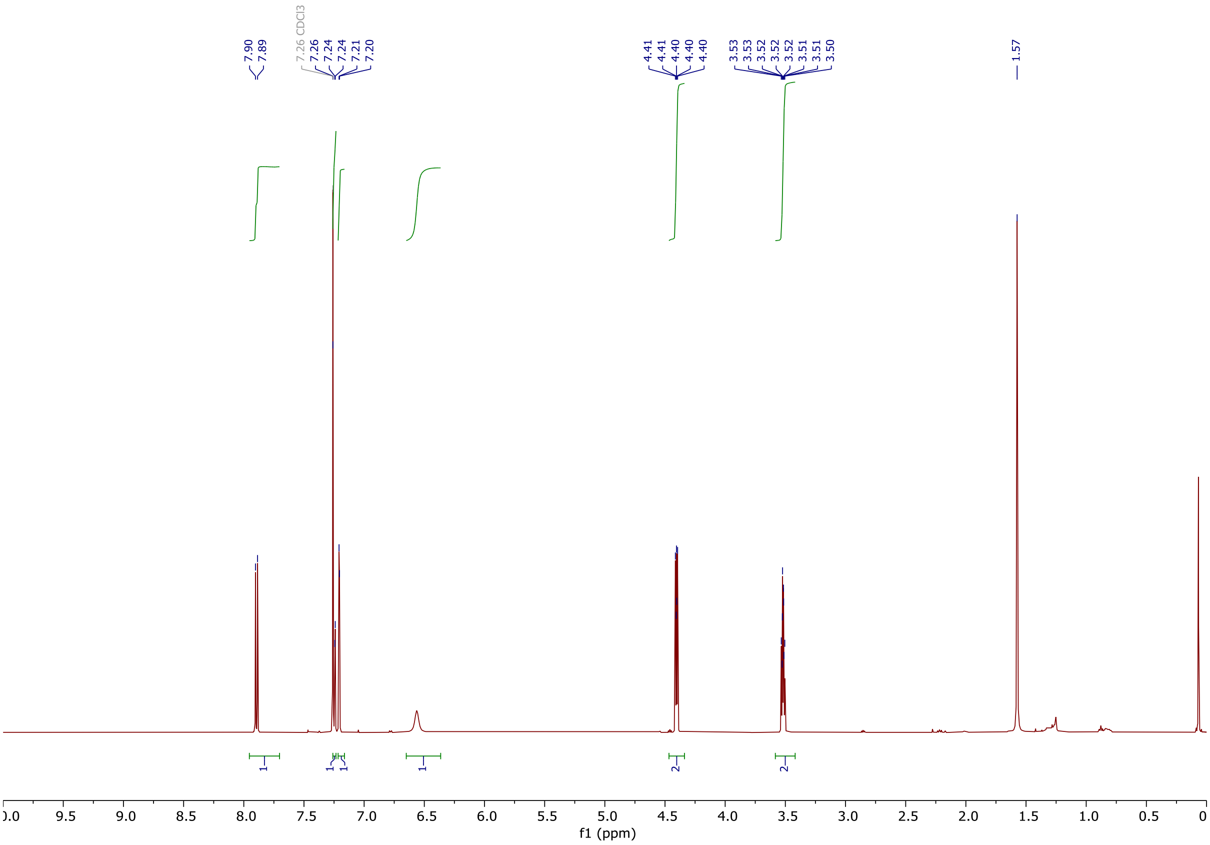

7.90
7.89
7.26 CDCl3
7.26
7.24
7.24
7.21
7.20
4.41
4.41
4.40
4.40
4.40
3.53
3.53
3.52
3.52
3.52
3.51
3.51
3.50
1.57
f1 (ppm)

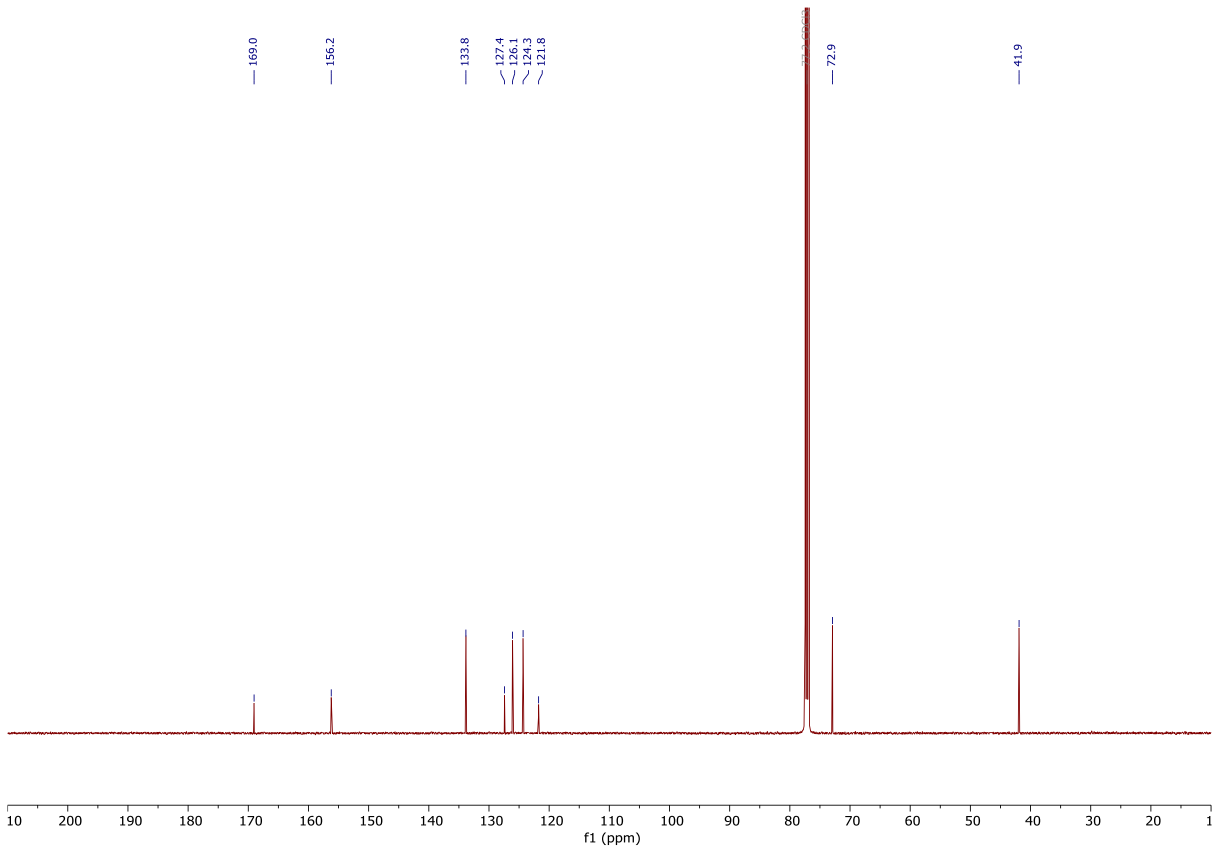
169.0
156.2
133.8
127.4
126.1
124.3
121.8
72.9
41.9
f1 (ppm)

3703

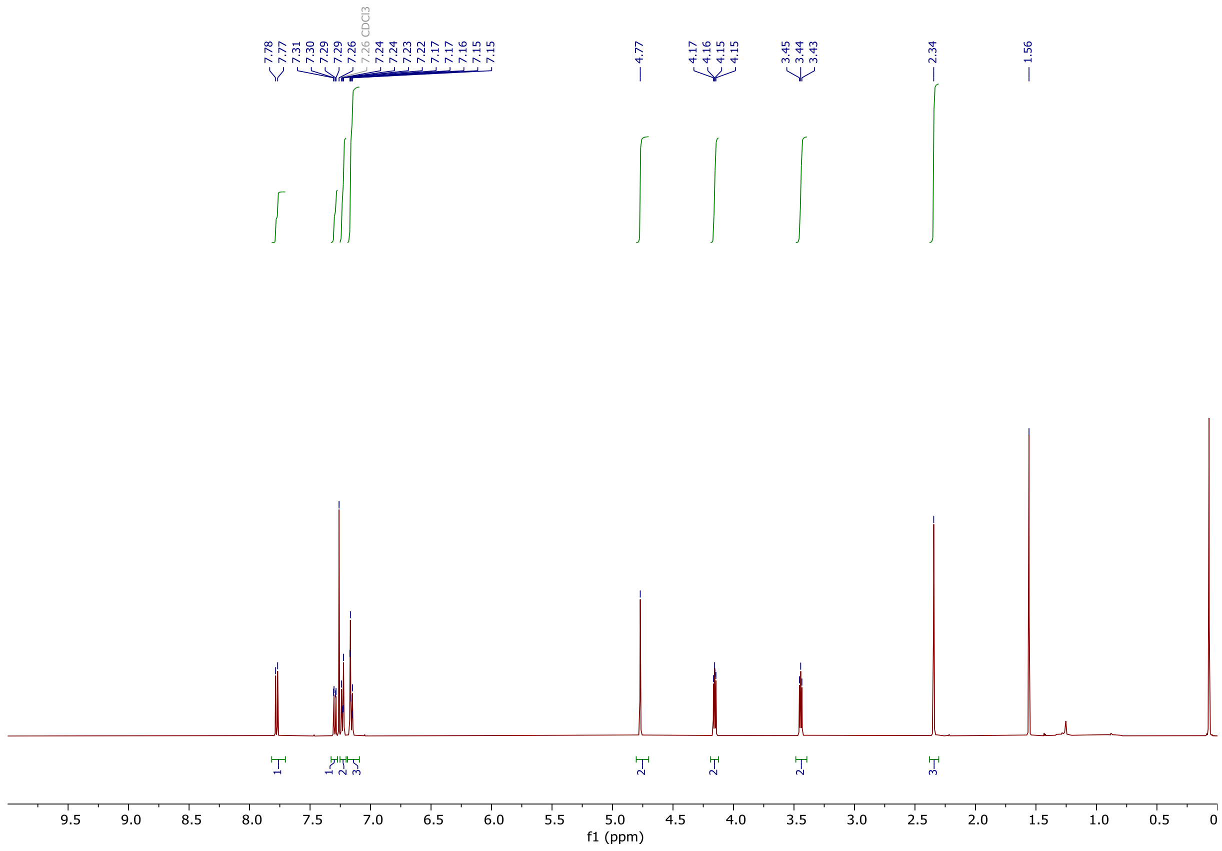
7.78
7.77
7.31
7.30
7.29
7.29
7.26
7.26 CDCl3
7.24
7.24
7.23
7.22
7.17
7.17
7.16
7.15
7.15
4.77
4.17
4.16
4.15
4.15
3.45
3.44
3.43
2.34
1.56
f1 (ppm)

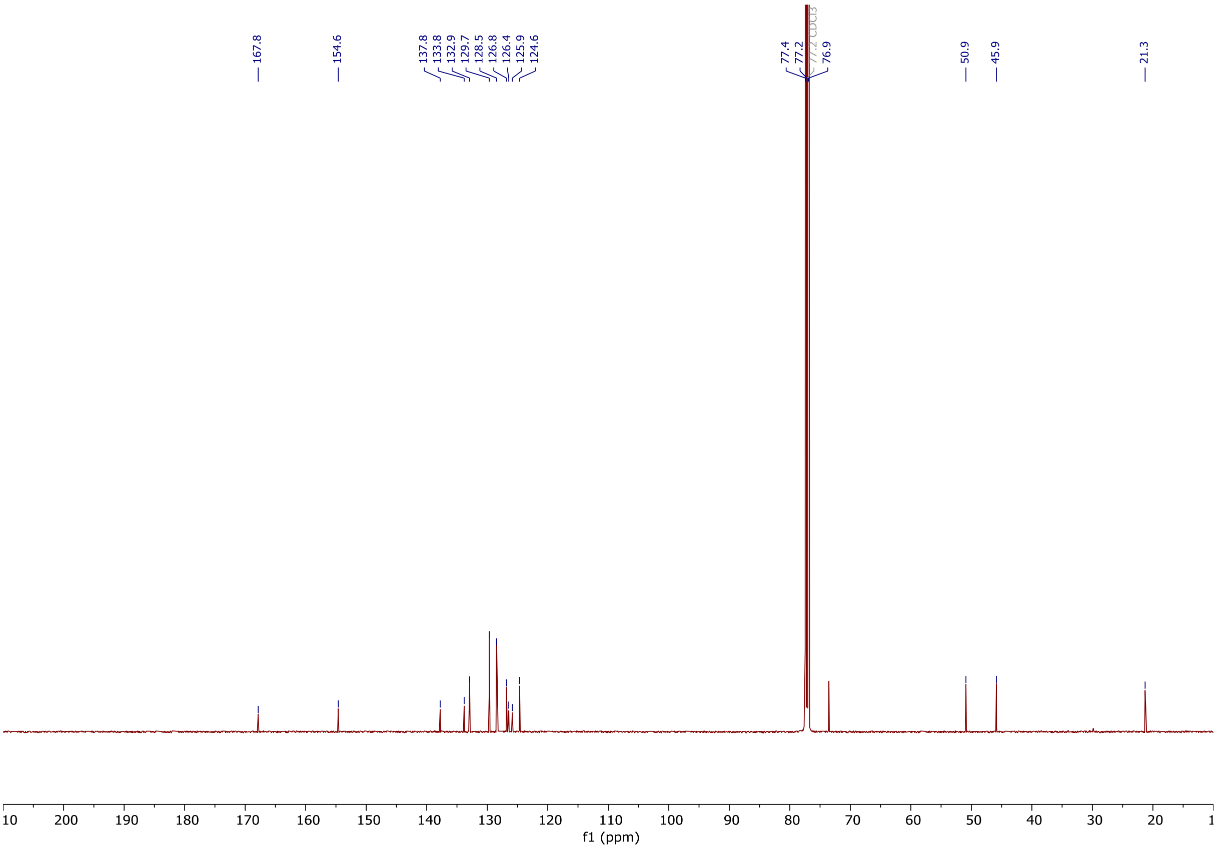
167.8
154.6
137.8
133.8
132.9
129.7
128.5
126.8
126.4
125.9
124.6
77.4
77.2
77.2 CDCl3
76.9
50.9
45.9
21.3
f1 (ppm)

3705

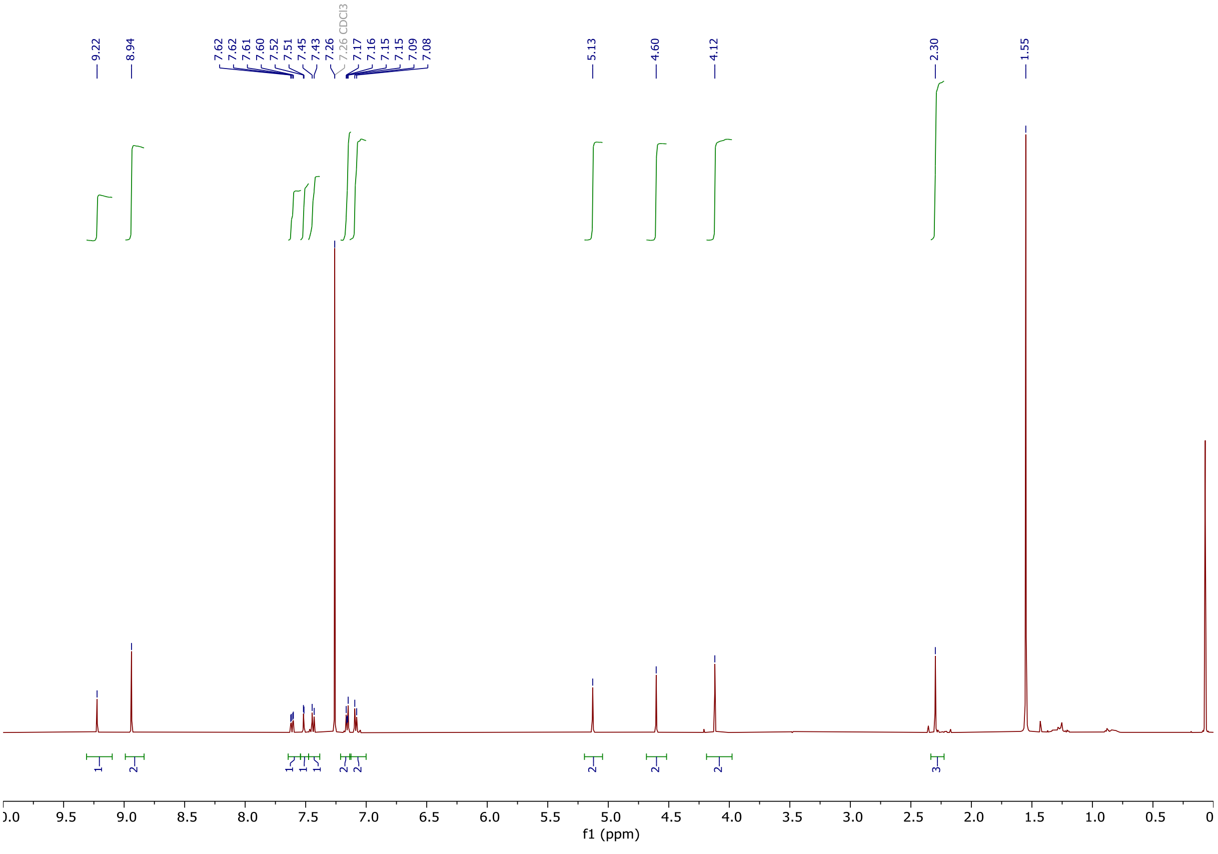
9.22
8.94
7.62
7.62
7.61
7.60
7.52
7.51
7.45
7.43
7.26 CDCl3
7.17
7.16
7.15
7.15
7.09
7.08
5.13
4.60
4.12
2.30
1.55
f1 (ppm)

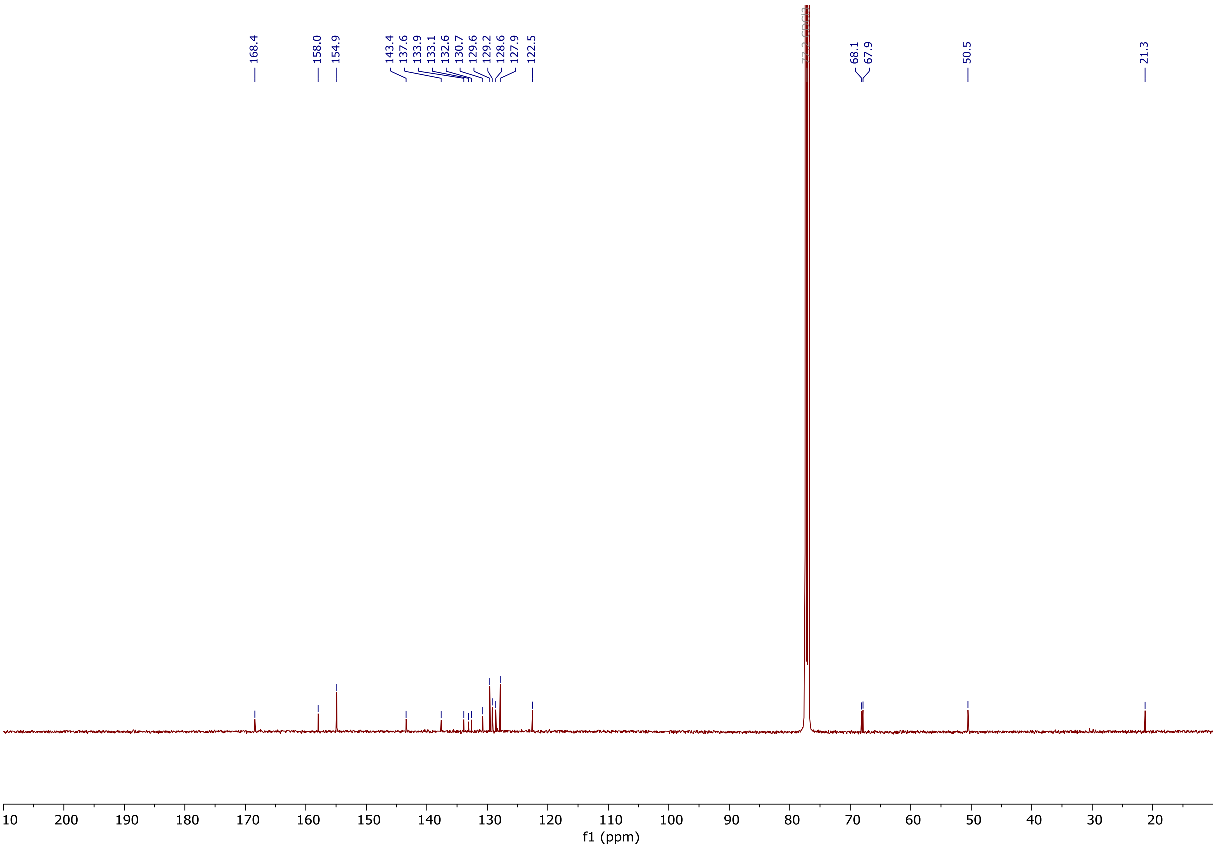

168.4
158.0
154.9
143.4
137.6
133.9
133.1
132.6
130.7
129.6
129.2
128.6
127.9
122.5
68.1
67.9
50.5
21.3
f1 (ppm)

3706

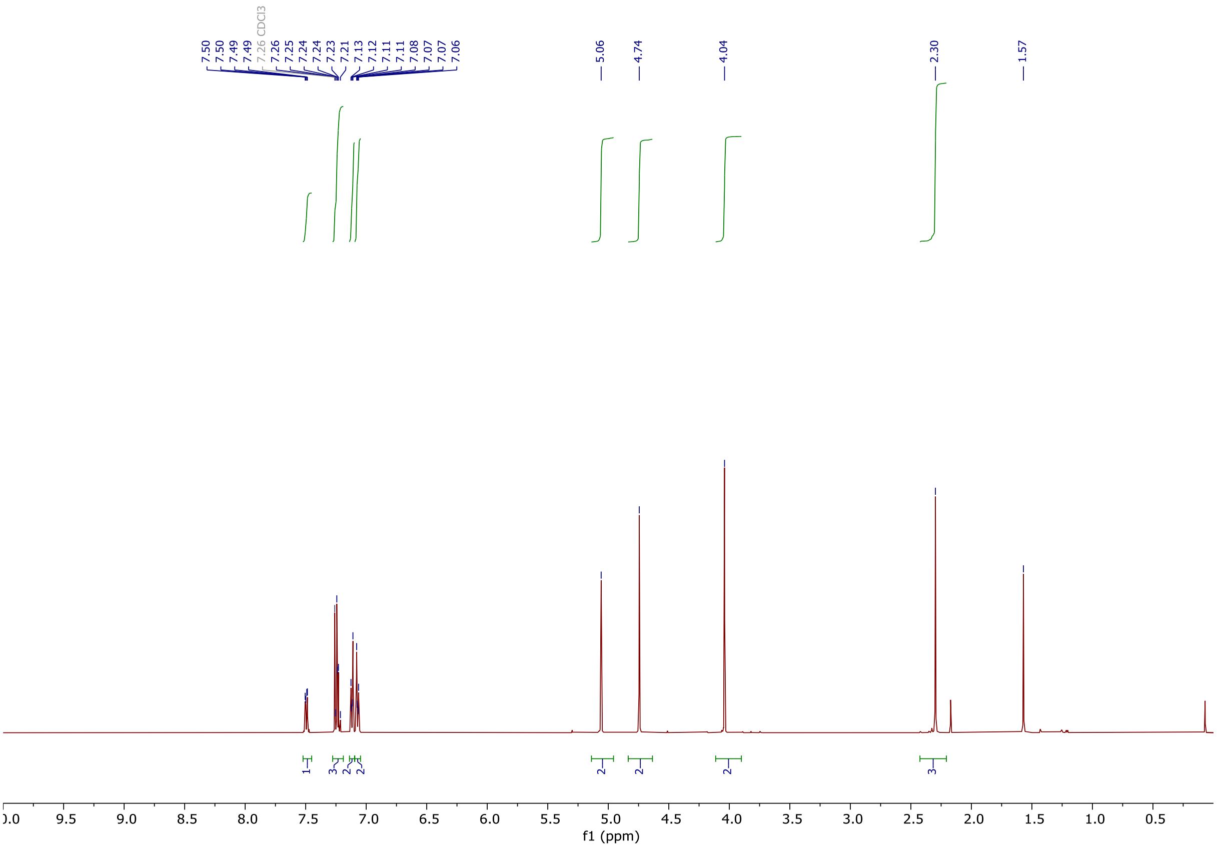

7.50
7.50
7.49
7.49
7.26 CDCl3
7.26
7.25
7.24
7.24
7.23
7.21
7.13
7.12
7.11
7.11
7.08
7.07
7.07
7.06
5.06
4.74
4.04
2.30
1.57
f1 (ppm)

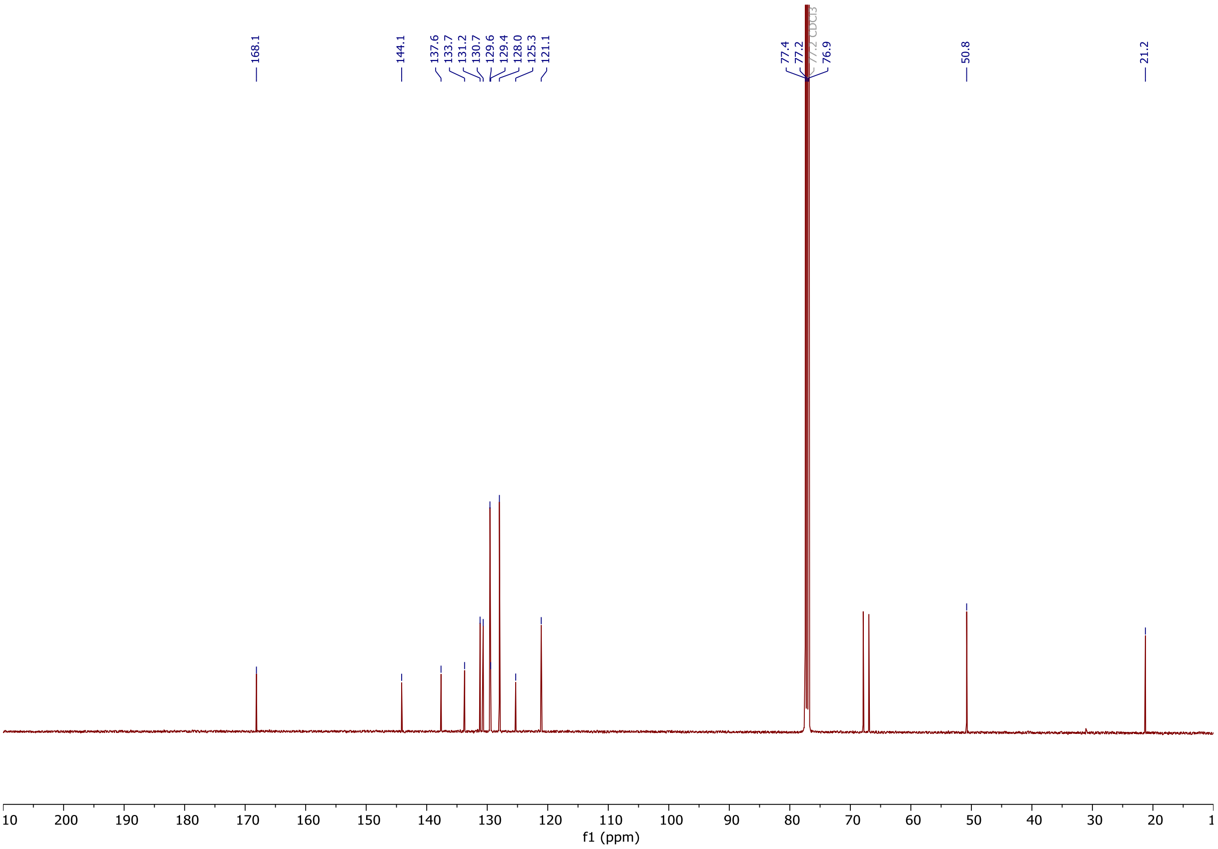
168.1
144.1
137.6
133.7
131.2
130.7
129.6
129.4
128.0
125.3
121.1
77.4
77.2
77.2 CDCl3
76.9
50.8
21.2
f1 (ppm)

3827

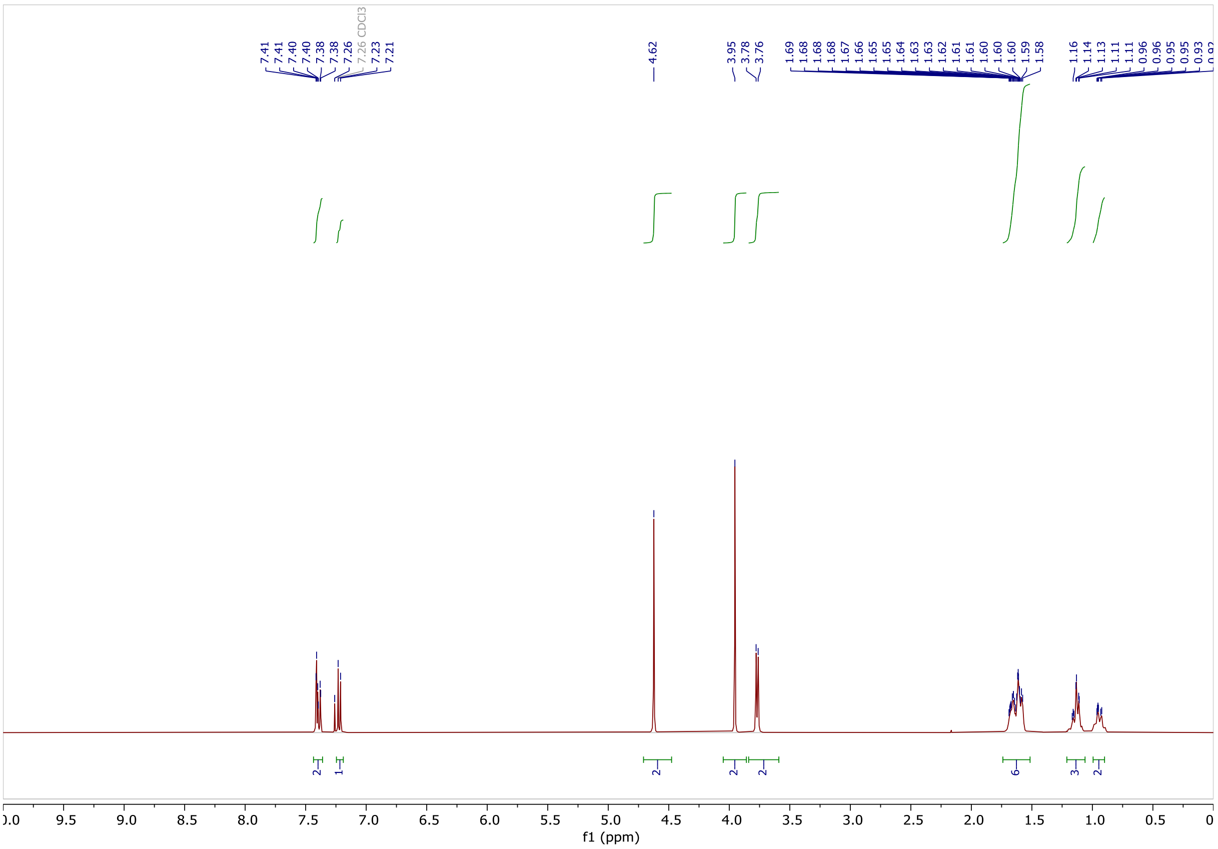
7.41
7.41
7.40
7.38
7.38
7.26
7.26 CDCl3
7.23
7.21
4.62
3.95
3.78
3.76
1.69
1.68
1.68
1.67
1.66
1.65
1.65
1.64
1.63
1.63
1.62
1.61
1.61
1.60
1.60
1.59
1.58
1.16
1.14
1.13
1.11
1.11
0.96
0.96
0.95
0.93
f1 (ppm)

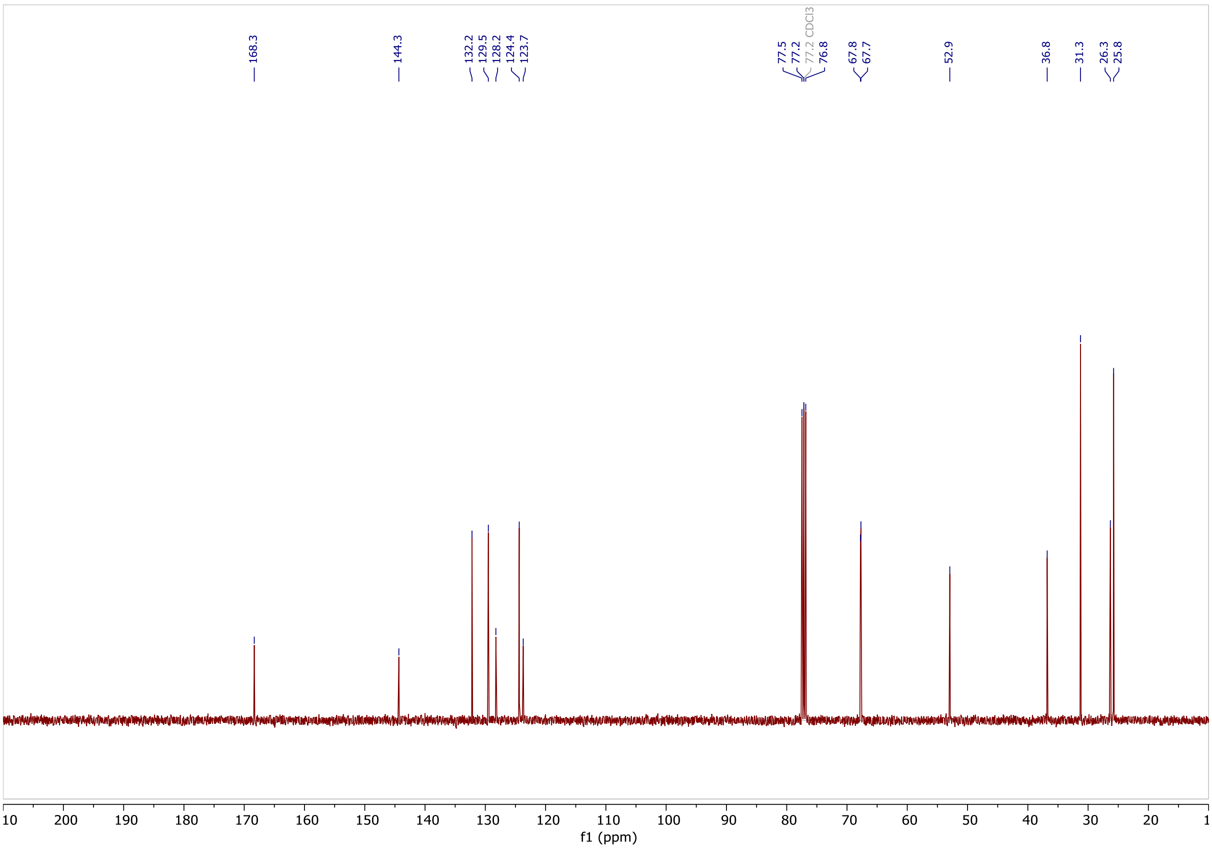
168.3
144.3
132.2
129.5
128.2
124.4
123.7
77.5
77.2
77.2 CDCl3
76.8
67.8
67.7
52.9
36.8
31.3
26.3
25.8
f1 (ppm)

3829

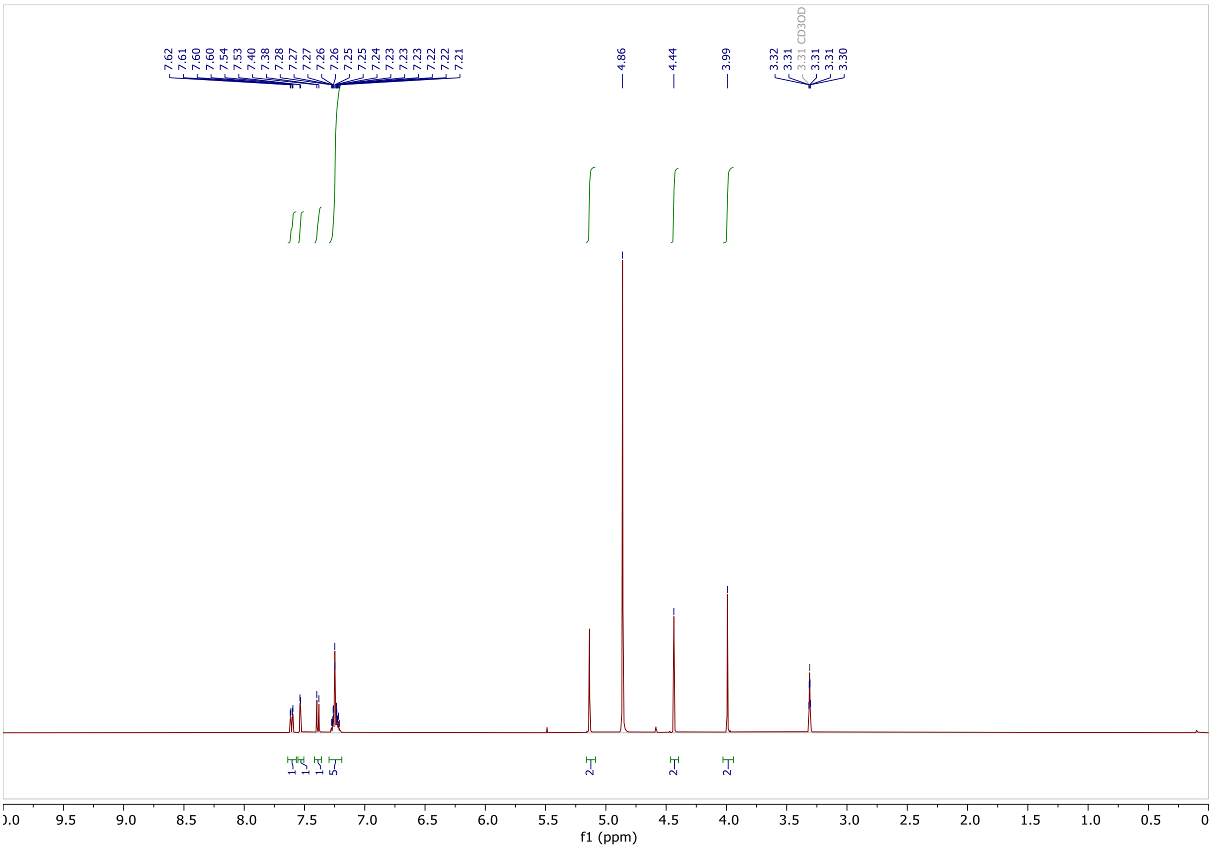
7.62
7.61
7.60
7.54
7.53
7.40
7.38
7.28
7.27
7.27
7.26
7.25
7.25
7.24
7.23
7.23
7.23
7.22
7.22
7.21
4.86
4.44
3.99
3.32
3.31
3.31 CD3OD
3.31
3.31
3.30
f1 (ppm)

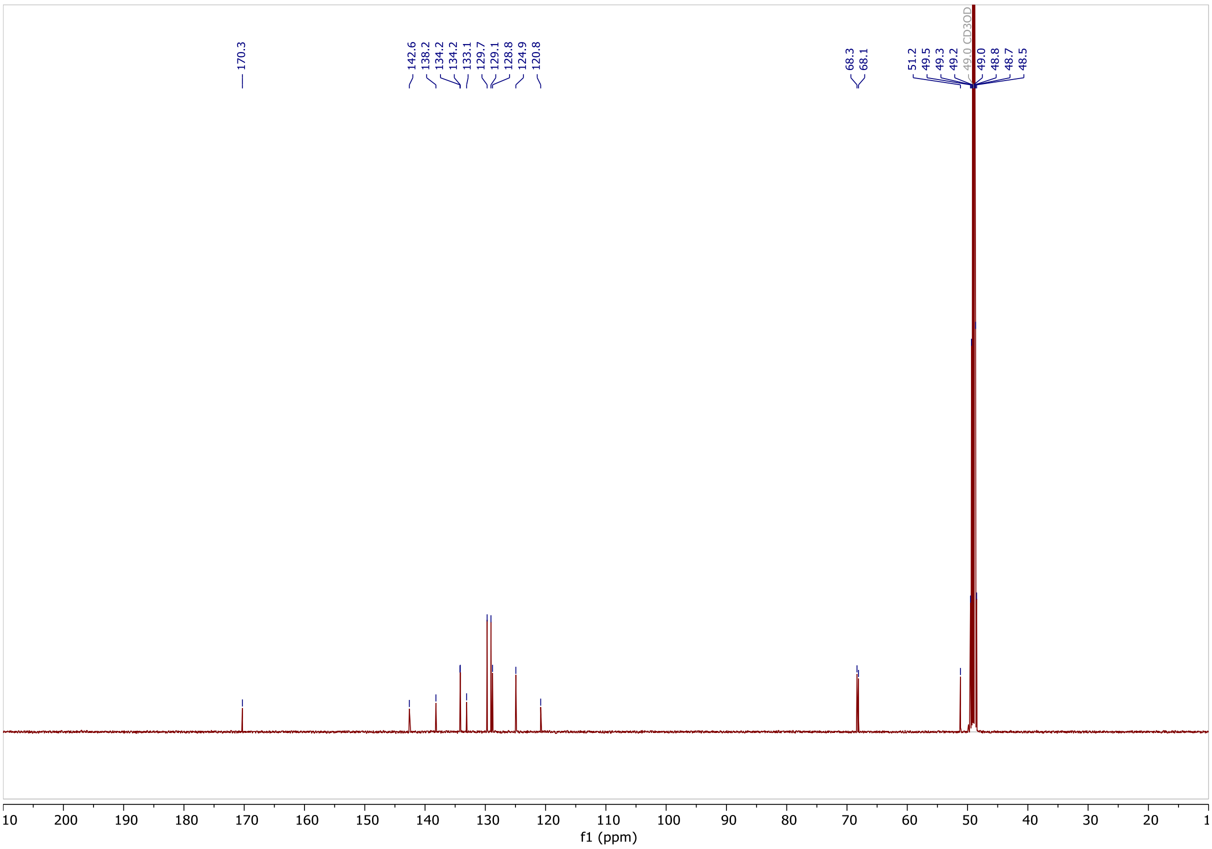
170.3
142.6
138.2
134.2
134.2
133.1
129.7
129.1
128.8
124.9
120.8
68.3
68.1
51.2
49.5
49.3
49.2
49.0 CD3OD
49.0
48.8
48.7
48.5
f1 (ppm)

3698

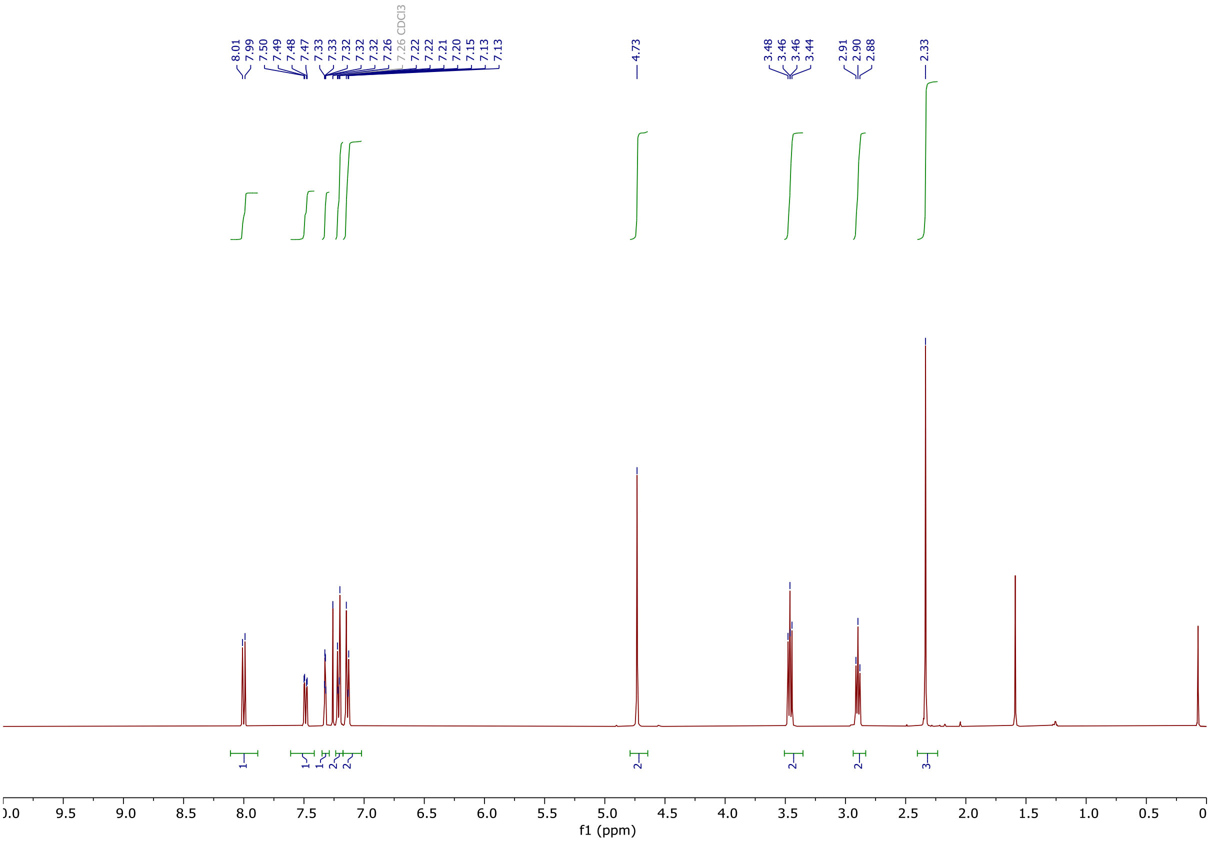
8.01
7.99
7.50
7.49
7.48
7.47
7.33
7.33
7.32
7.32
7.32
7.26 CDCl3
7.22
7.22
7.21
7.20
7.15
7.13
7.13
4.73
3.48
3.46
3.46
3.44
2.91
2.90
2.88
2.33
f1 (ppm)

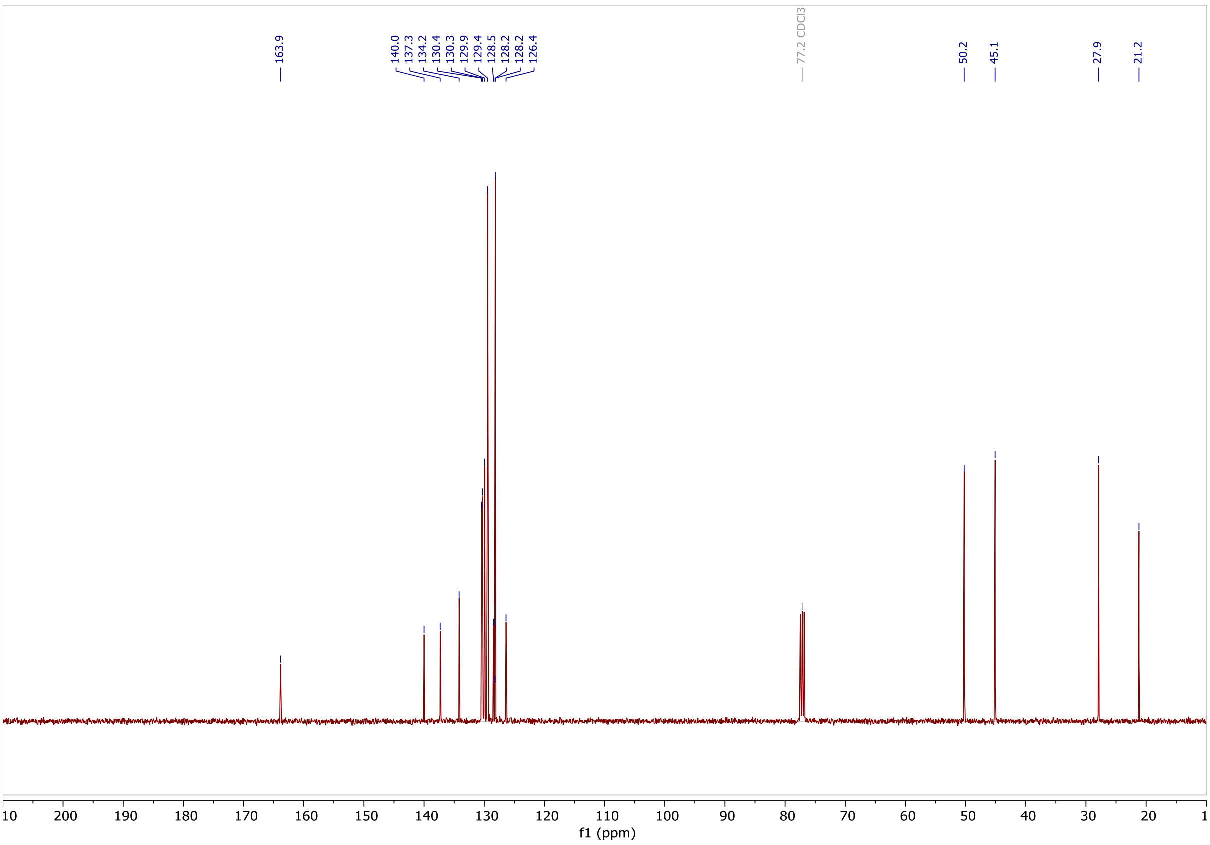
163.9
140.0
137.3
134.2
130.4
130.3
129.9
129.4
128.5
128.2
128.2
126.4
77.2 CDCl3
50.2
45.1
27.9
21.2
f1 (ppm)

4119

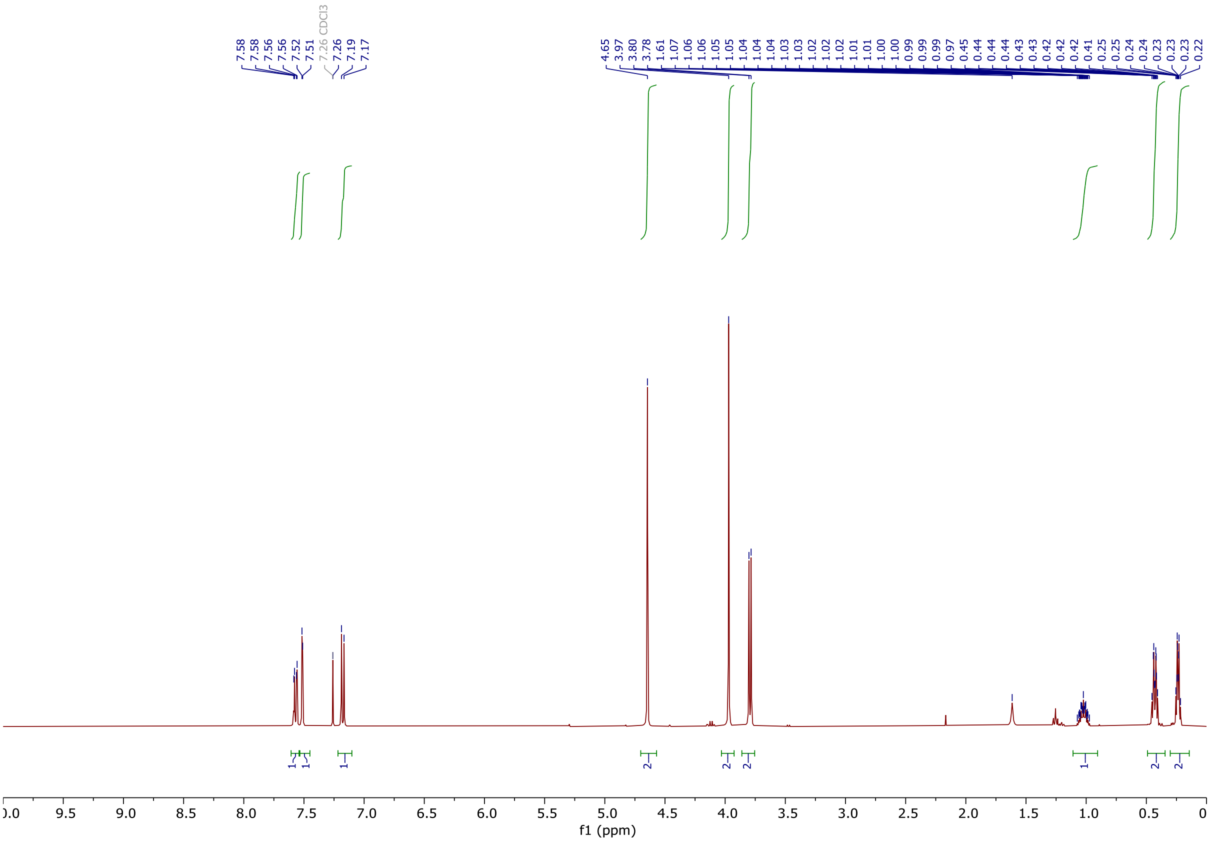
7.58
7.58
7.56
7.56
7.52
7.51
7.26 CDCl3
7.26
7.19
7.17
4.65
3.97
3.80
3.78
1.61
1.07
1.06
1.06
1.05
1.05
1.04
1.04
1.04
1.03
1.03
1.02
1.02
1.02
1.01
1.01
1.00
0.99
0.99
0.99
0.97
0.45
0.44
0.44
0.44
0.43
0.43
0.42
0.42
0.42
0.41
0.25
0.25
0.24
0.24
0.23
0.23
0.23
0.23
0.22
1
1
1
2
2
2
1
2
2
f1 (ppm)

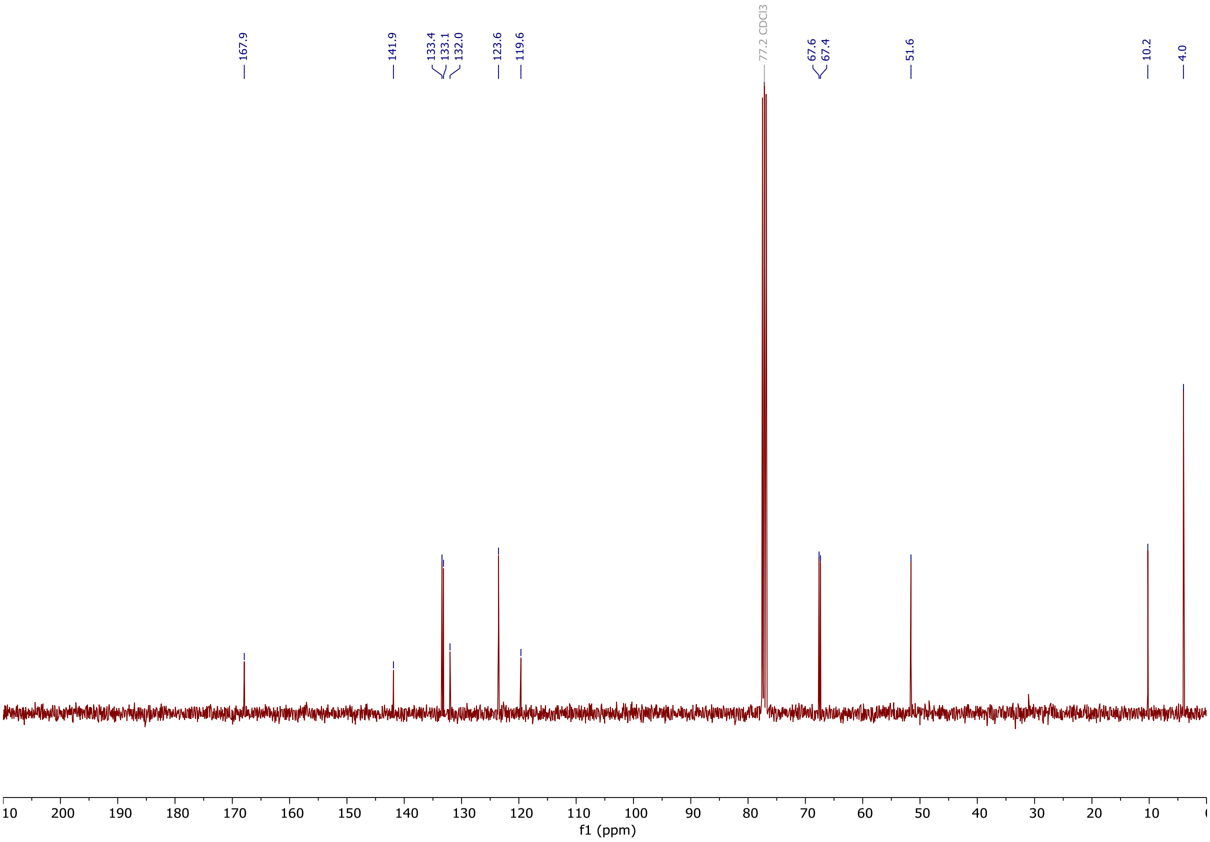
167.9
141.9
133.4
133.1
132.0
123.6
119.6
77.2 CDCl3
67.6
67.4
51.6
10.2
4.0
f1 (ppm)

4118a

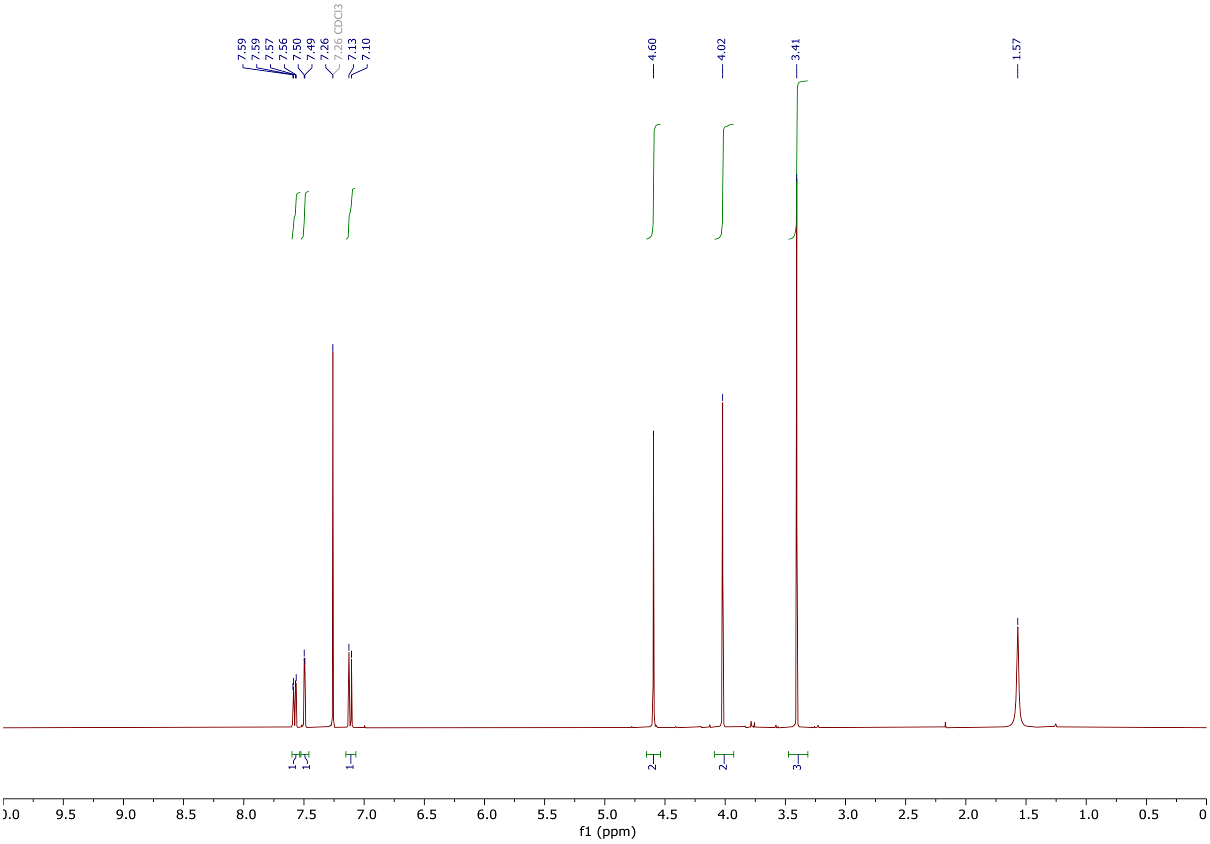
7.59
7.59
7.57
7.56
7.50
7.49
7.26 CDCl3
7.13
7.10
4.60
4.02
3.41
1.57
1
1
1
2
2
3
f1 (ppm)

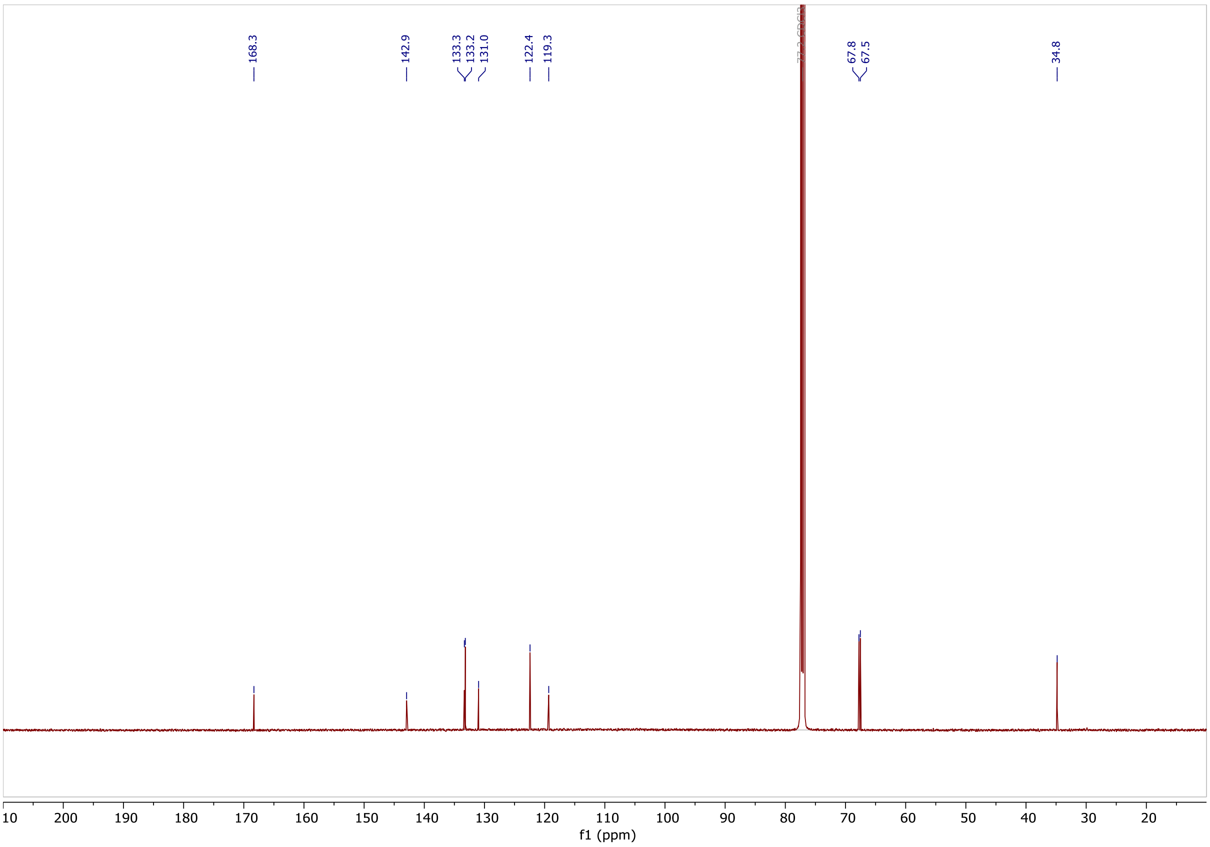
168.3
142.9
133.3
133.2
131.0
122.4
119.3
67.8
67.5
34.8
f1 (ppm)

3145

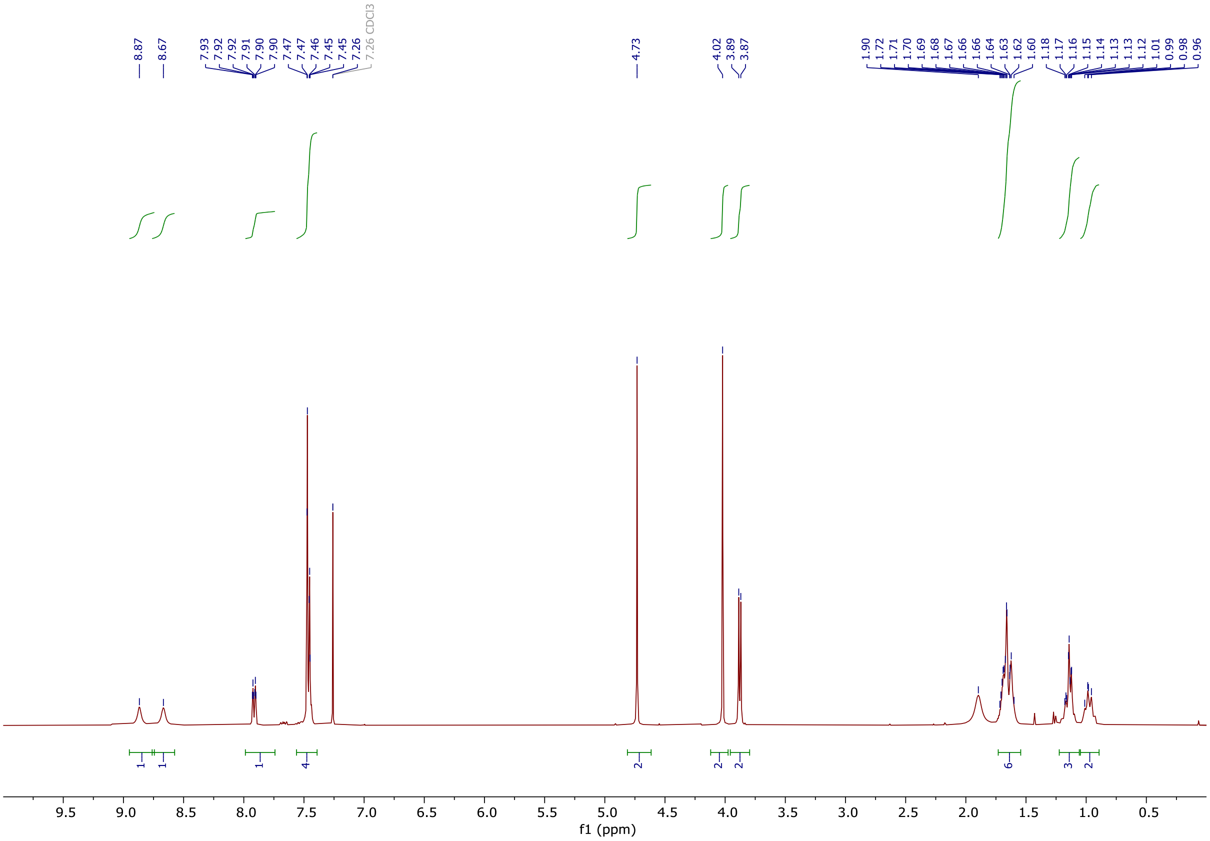

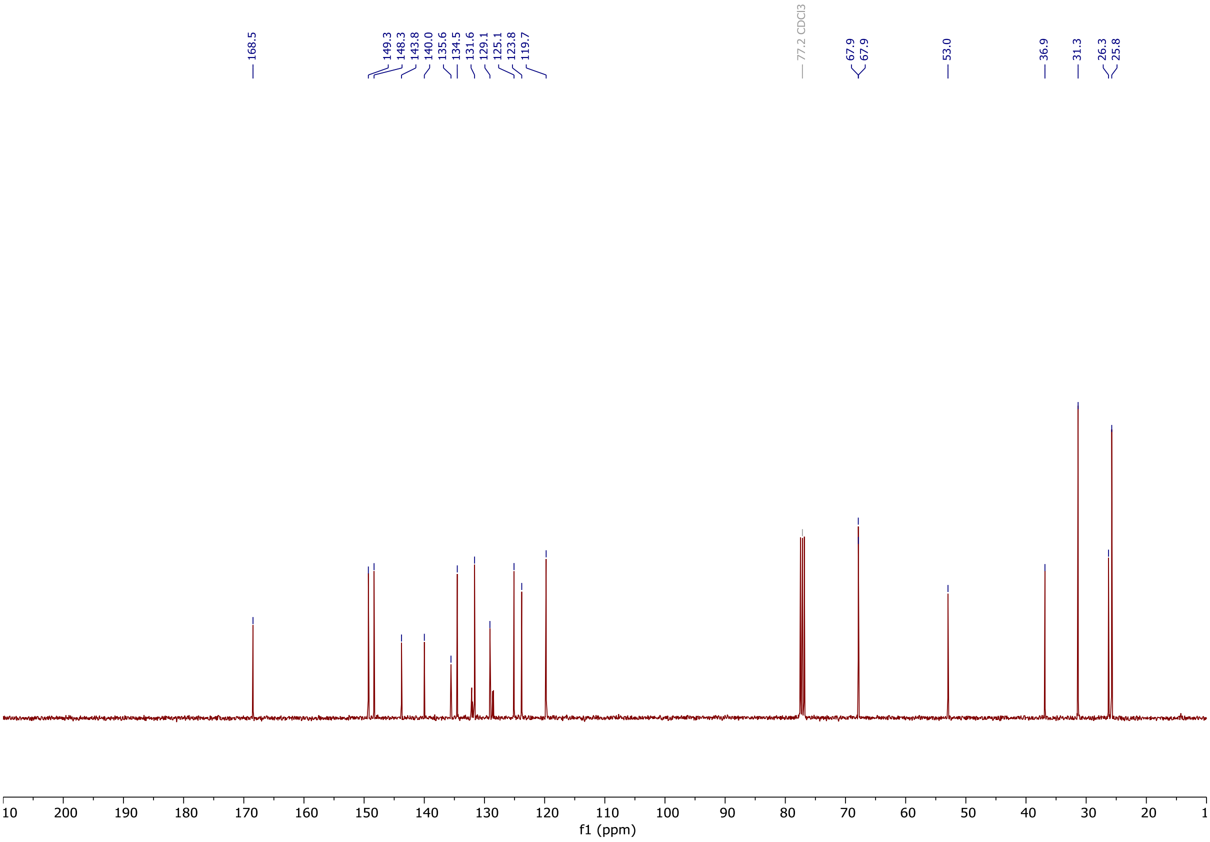

3593

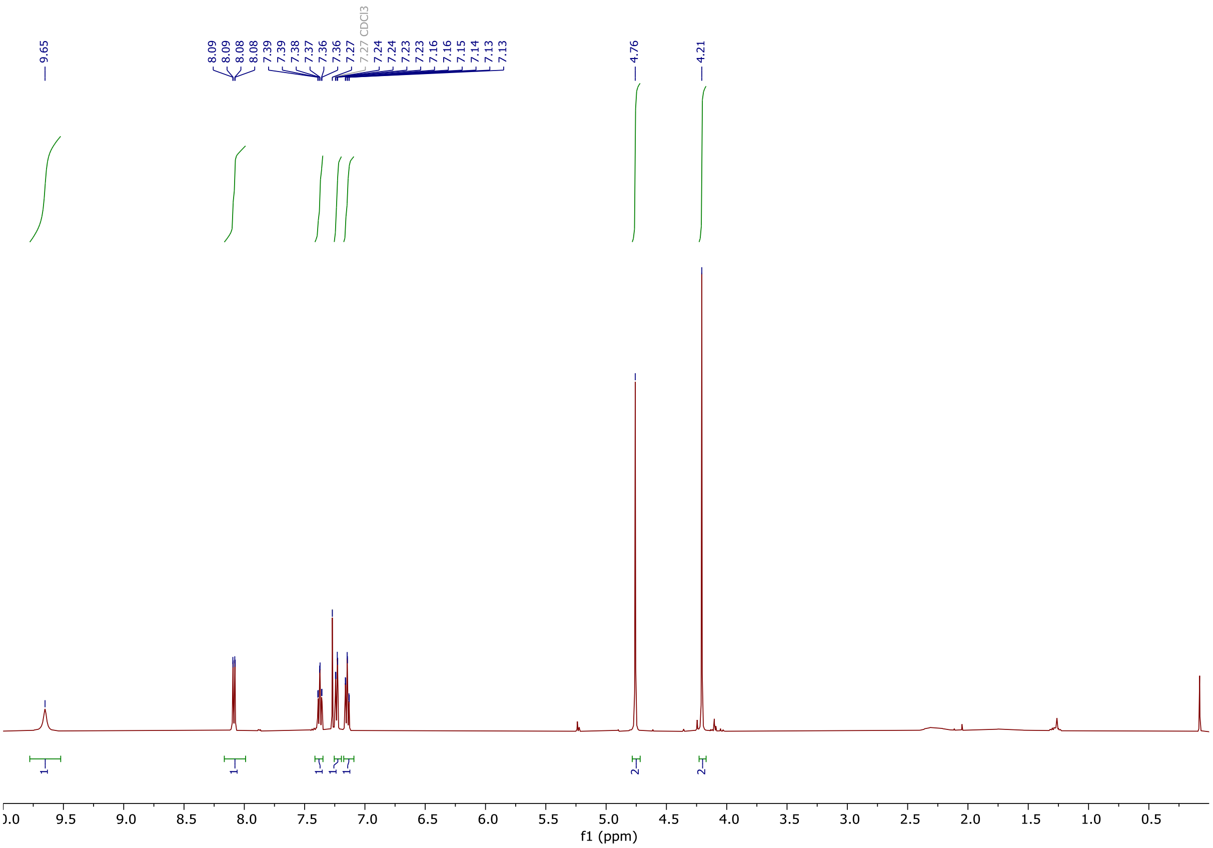
9.65
8.09
8.09
8.08
8.08
7.39
7.39
7.38
7.37
7.36
7.36
7.27 CDCl3
7.27
7.24
7.24
7.24
7.23
7.23
7.16
7.16
7.15
7.14
7.13
7.13
4.76
4.21
f1 (ppm)

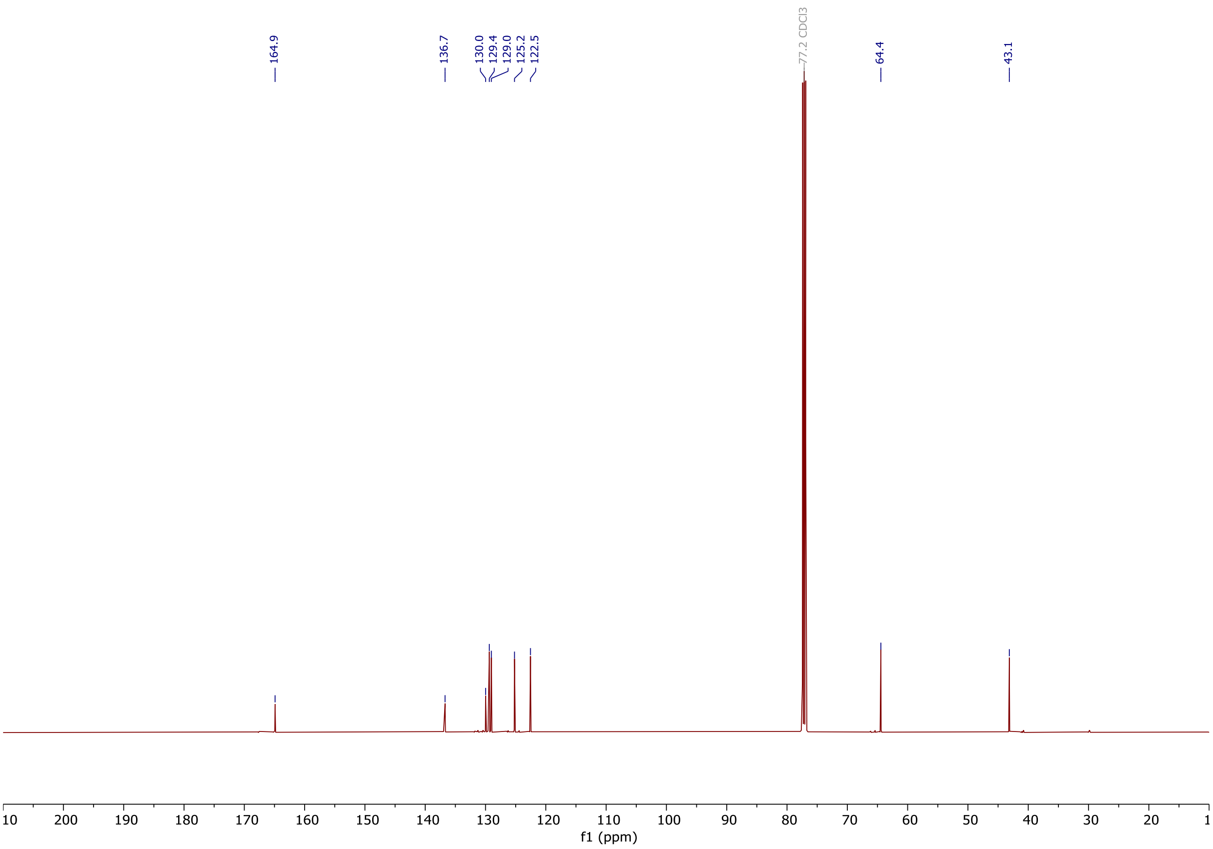
164.9
136.7
130.0
129.4
129.0
125.2
122.5
77.2 CDCl3
64.4
43.1
f1 (ppm)

3594

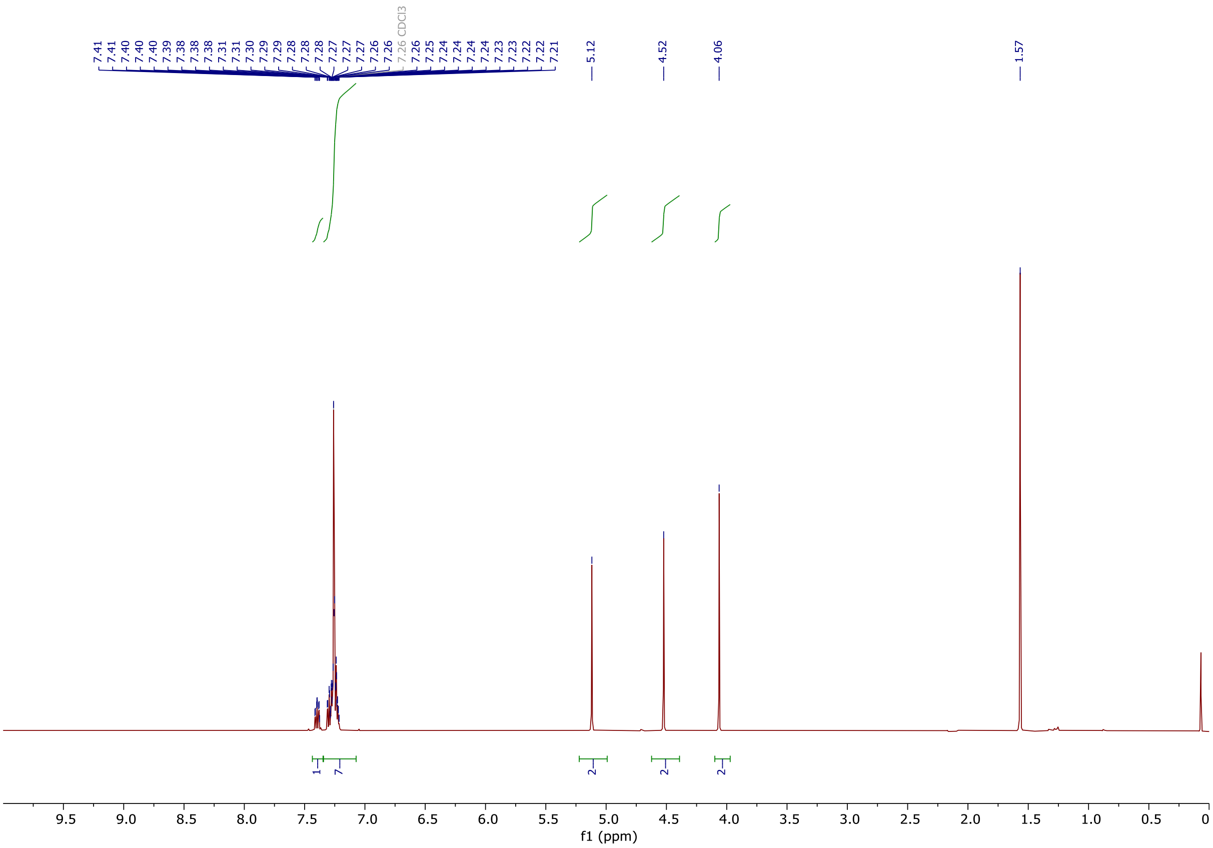

7.41
7.41
7.40
7.40
7.40
7.39
7.38
7.38
7.38
7.31
7.31
7.30
7.29
7.29
7.28
7.28
7.28
7.27
7.27
7.27
7.26
7.26 CDCl3
7.26
7.25
7.24
7.24
7.24
7.24
7.23
7.23
7.22
7.22
7.21
5.12
4.52
4.06
1.57
1
7
2
2
2
f1 (ppm)

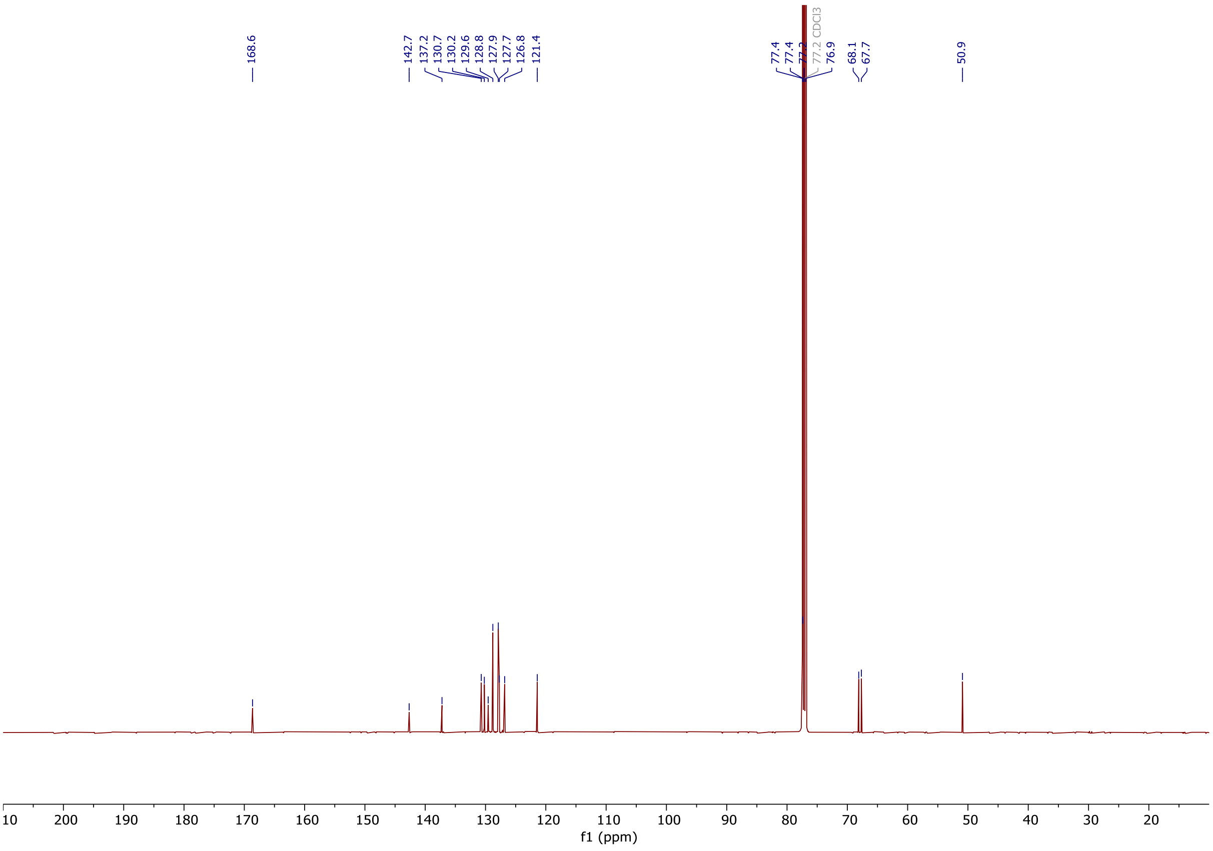

168.6
142.7
137.2
130.7
130.2
129.6
128.8
127.9
127.7
126.8
121.4
77.4
77.4
77.2 CDCl3
76.9
68.1
67.7
50.9
f1 (ppm)

3596

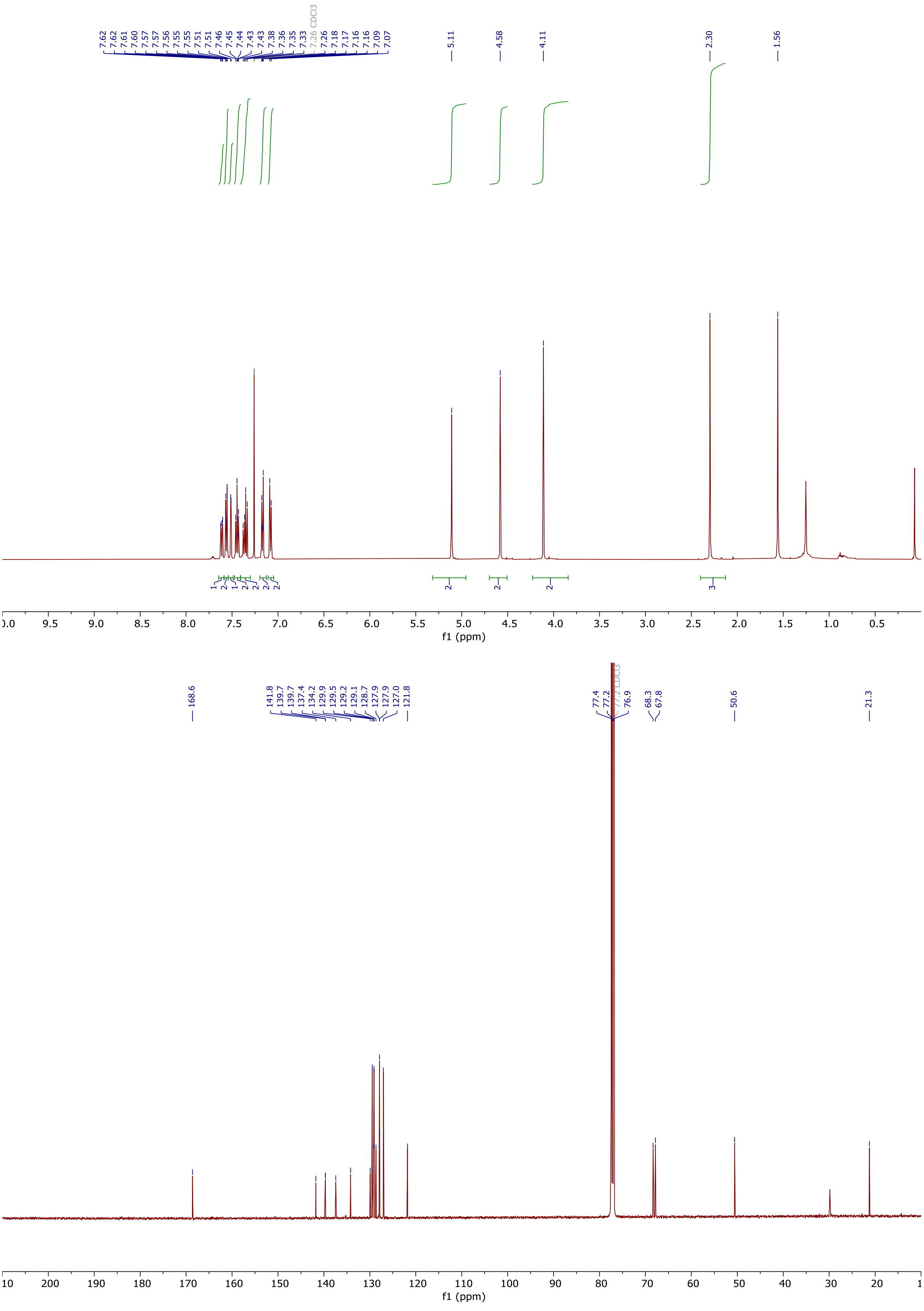
7.62
7.62
7.61
7.60
7.57
7.56
7.55
7.55
7.51
7.51
7.46
7.45
7.44
7.43
7.43
7.38
7.36
7.35
7.33
7.26 CDCl3
7.26
7.18
7.17
7.16
7.16
7.09
7.07
5.11
4.58
4.11
2.30
1.56
f1 (ppm)
168.6
141.8
139.7
139.7
137.4
134.2
129.9
129.5
129.2
129.1
128.7
127.9
127.9
127.0
121.8
77.4
77.2
77.2 CDCl3
76.9
68.3
67.8
50.6
21.3
f1 (ppm)

3599

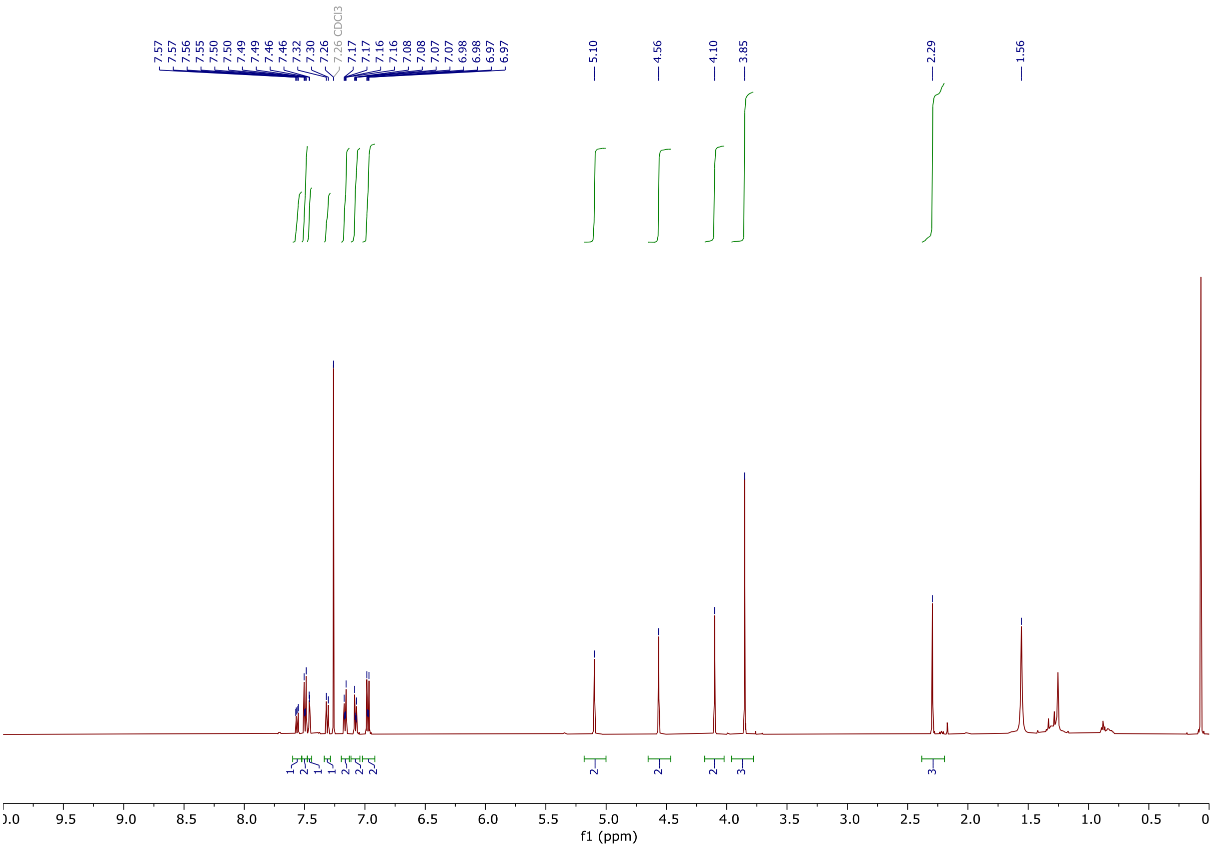

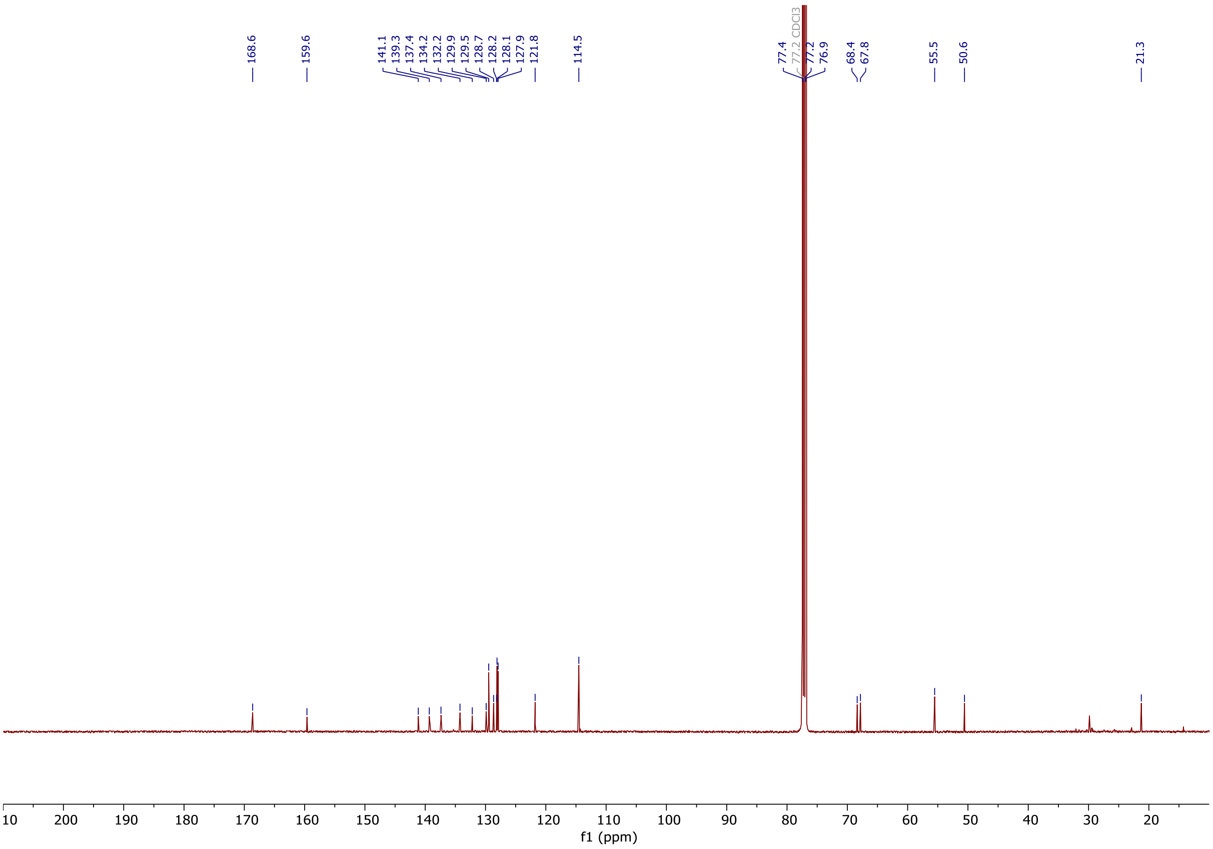

3600

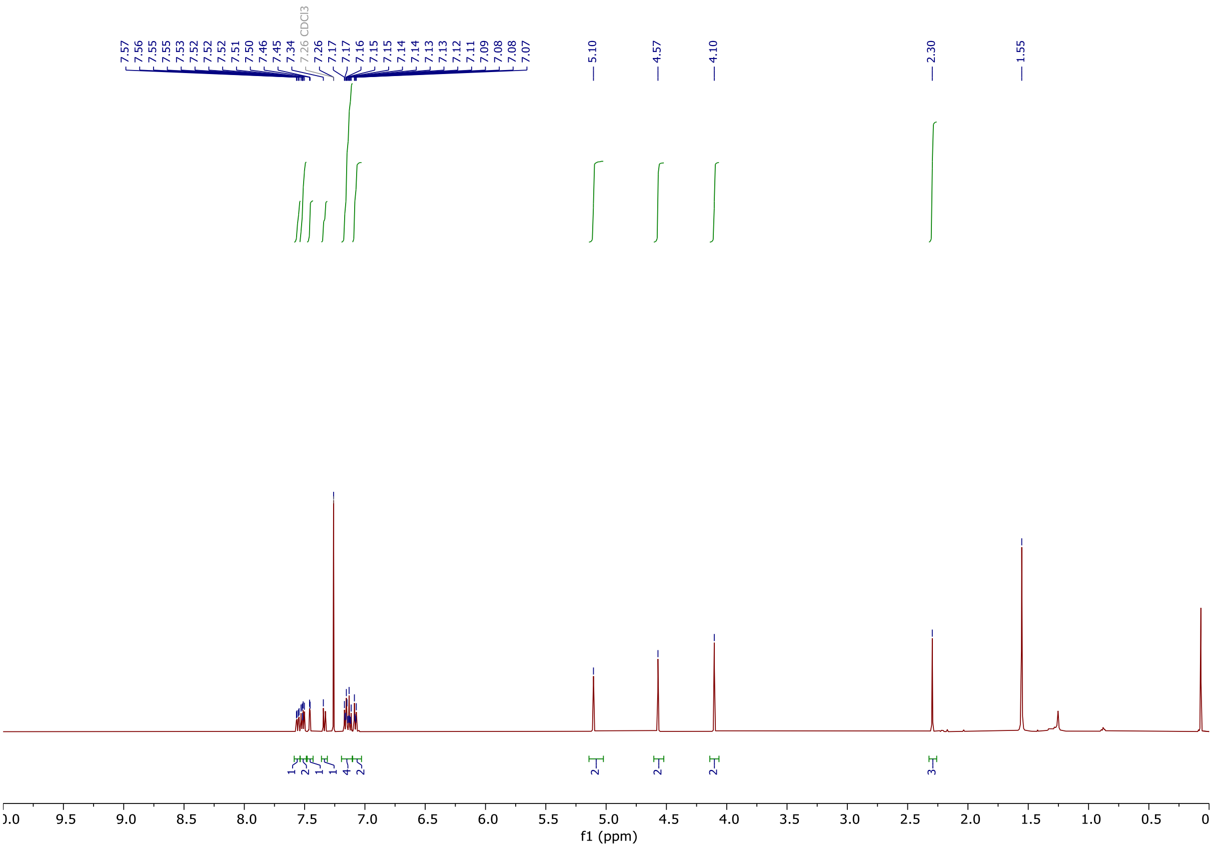
7.57 7.56 7.55 7.55 7.53 7.52 7.52 7.52 7.51 7.50 7.46 7.45 7.34 7.26 CDCl3 7.26 7.17 7.17 7.17 7.16 7.15 7.15 7.14 7.14 7.13 7.13 7.12 7.11 7.09 7.08 7.08 7.07
5.10
4.57
4.10
2.30
1.55
f1 (ppm)

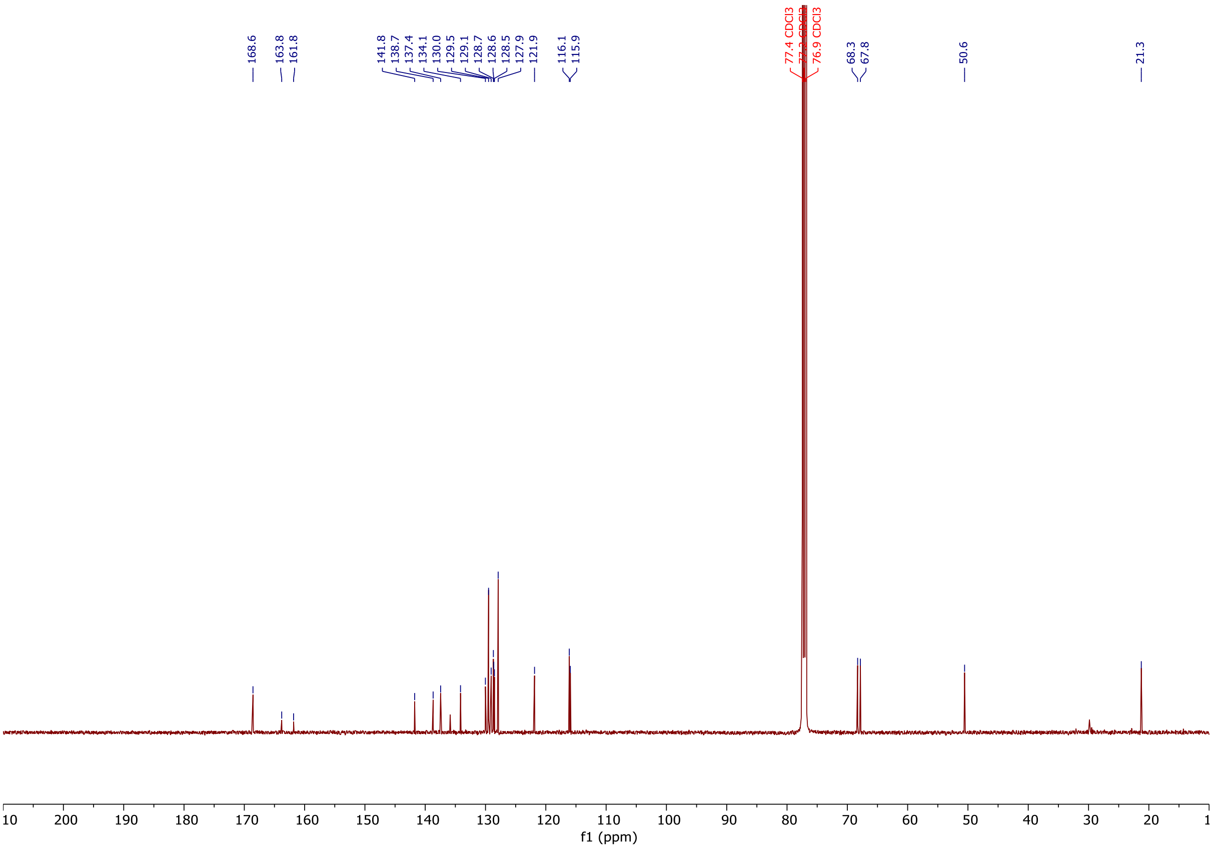
168.6 163.8 161.8
141.8 138.7 137.4 134.1 130.0 129.5 129.1 128.7 128.6 128.5 127.9 121.9
116.1 115.9
77.4 CDCl3 76.9 CDCl3
68.3 67.8
50.6
21.3
f1 (ppm)

3601

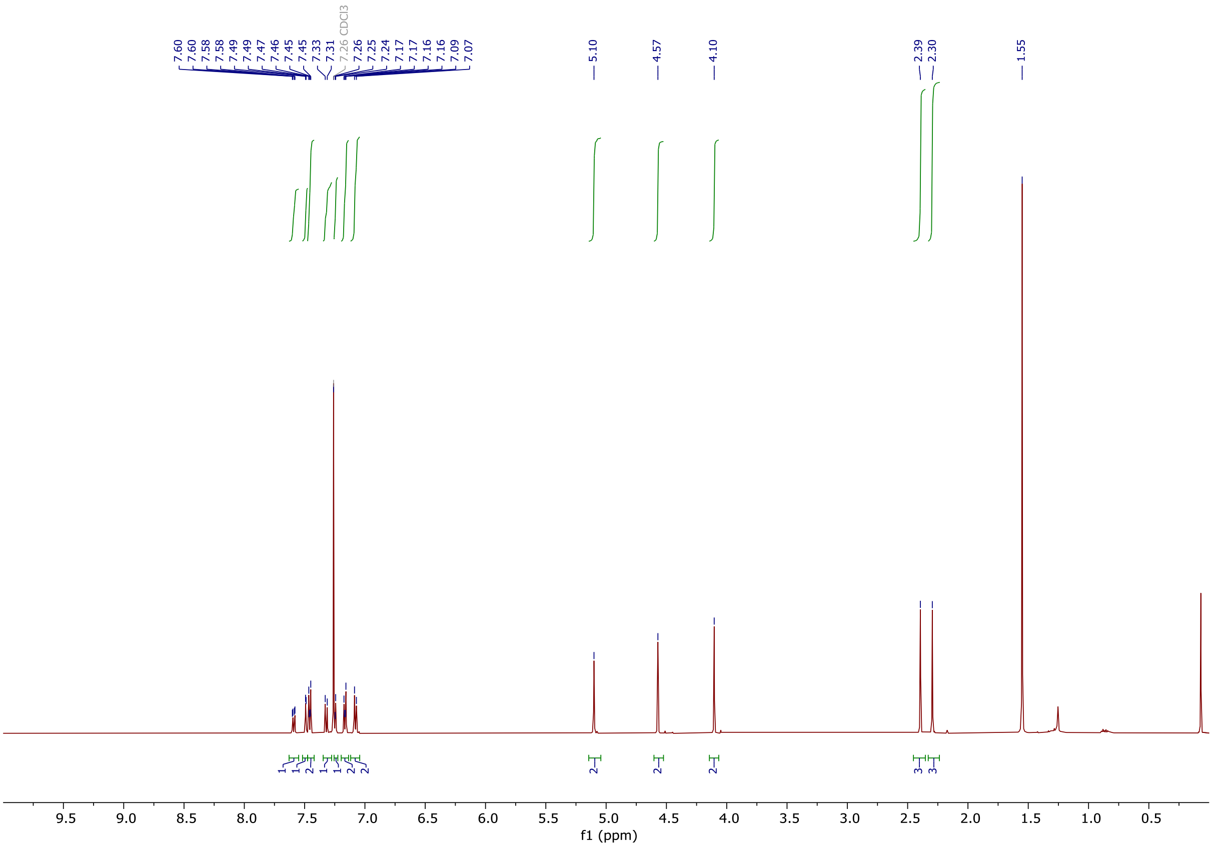
7.60 7.60 7.58 7.58 7.49 7.49 7.47 7.46 7.45 7.45 7.33 7.31 7.26 CDCl3 7.26 7.25 7.24 7.17 7.17 7.16 7.16 7.09 7.07
5.10
4.57
4.10
2.39 2.30
1.55
1 1 2 1 1 2 2
2
2
2
3 3
9.5 9.0 8.5 8.0 7.5 7.0 6.5 6.0 5.5 5.0 4.5 4.0 3.5 3.0 2.5 2.0 1.5 1.0 0.5
f1 (ppm)

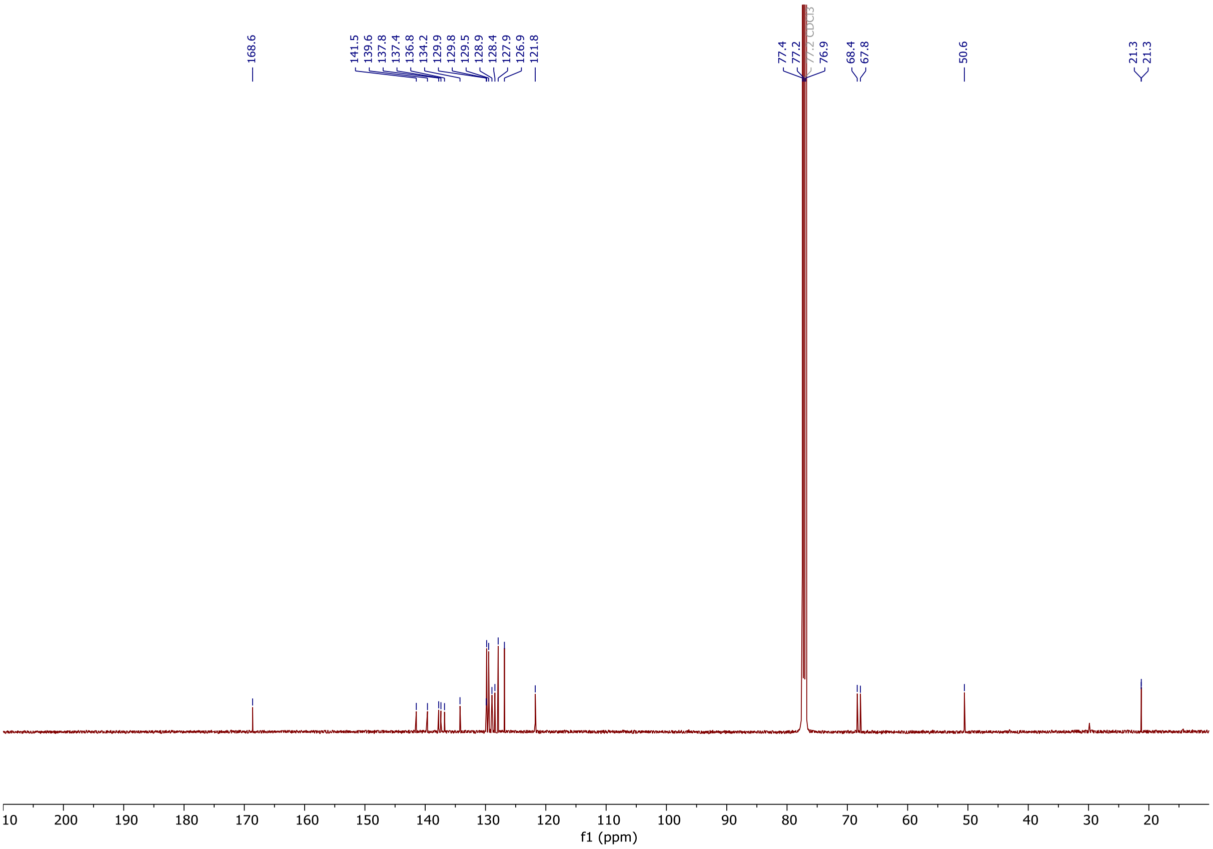
168.6
141.5 139.6 137.8 137.4 136.8 134.2 129.9 129.8 129.5 128.9 128.4 127.9 126.9 121.8
77.4 77.2 77.2 CDCl3 76.9
68.4 67.8
50.6
21.3 21.3
10 200 190 180 170 160 150 140 130 120 110 100 90 80 70 60 50 40 30 20
f1 (ppm)

3697

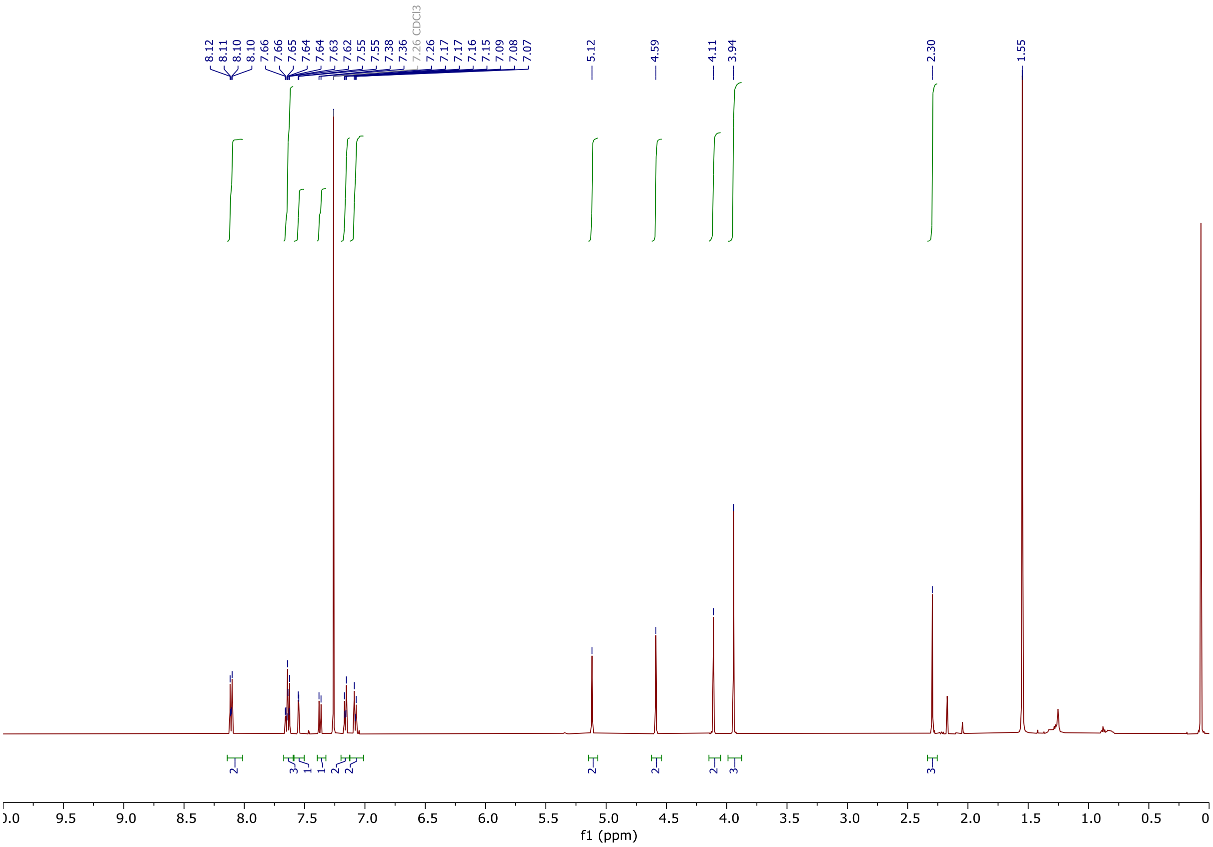
8.12
8.11
8.10
8.10
7.66
7.66
7.65
7.64
7.64
7.63
7.62
7.55
7.55
7.38
7.36
7.26 CDCl3
7.26
7.17
7.17
7.16
7.15
7.09
7.08
7.07
5.12
4.59
4.11
3.94
2.30
1.55
f1 (ppm)

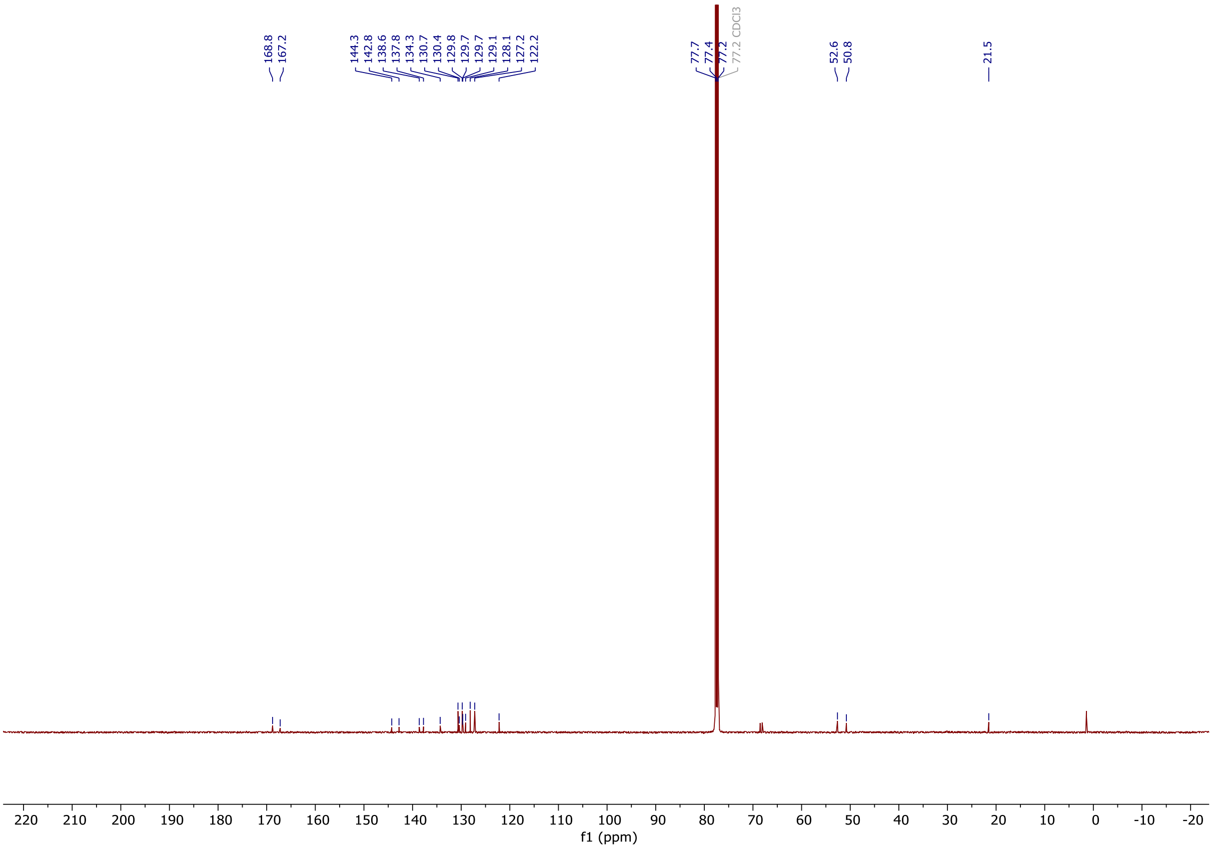
168.8
167.2
144.3
142.8
138.6
137.8
134.3
130.7
130.4
129.8
129.7
129.7
129.1
128.1
127.2
122.2
77.7
77.4
77.2
77.2 CDCl3
52.6
50.8
21.5
f1 (ppm)

3701

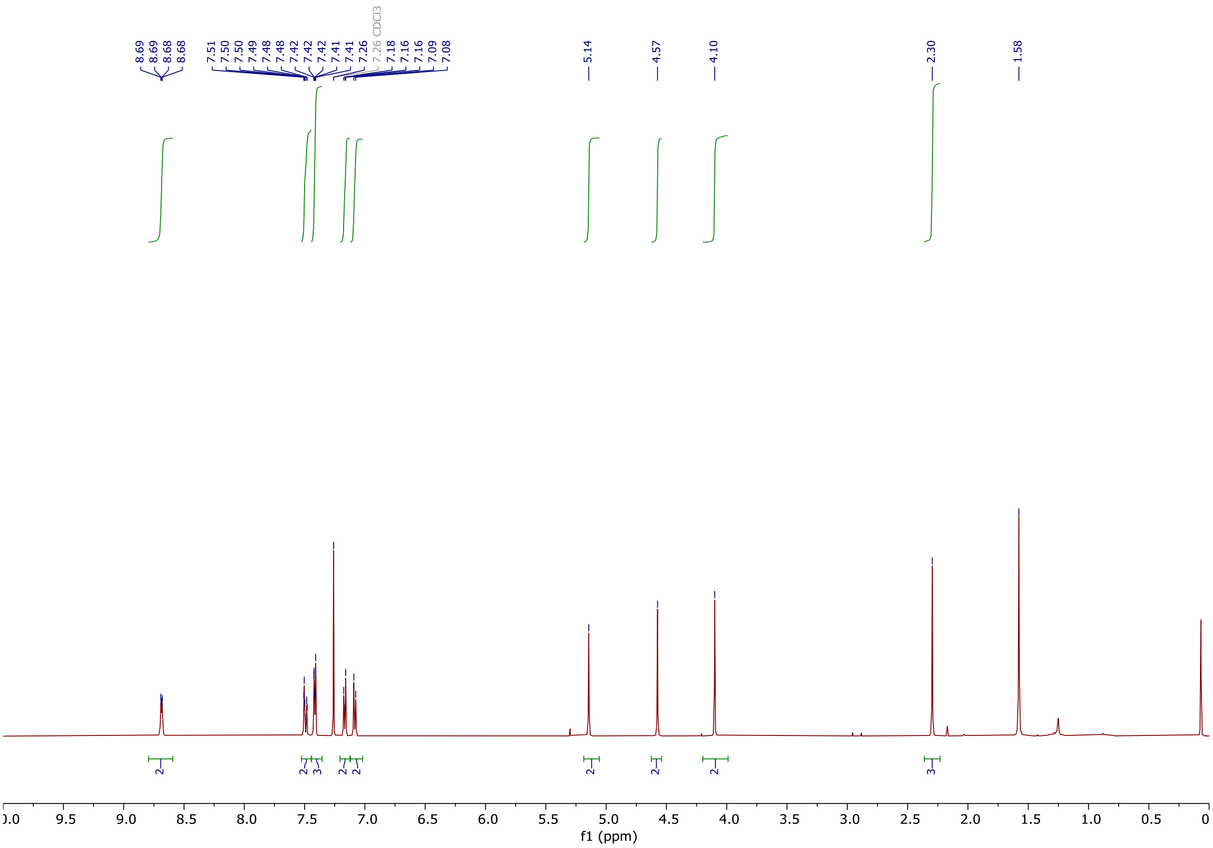
8.69
8.69
8.68
8.68
7.51
7.50
7.50
7.49
7.48
7.48
7.42
7.42
7.42
7.41
7.41
7.26 CDCl3
7.18
7.16
7.16
7.09
7.08
5.14
4.57
4.10
2.30
1.58
2
2
3
2
2
2
2
2
3
f1 (ppm)

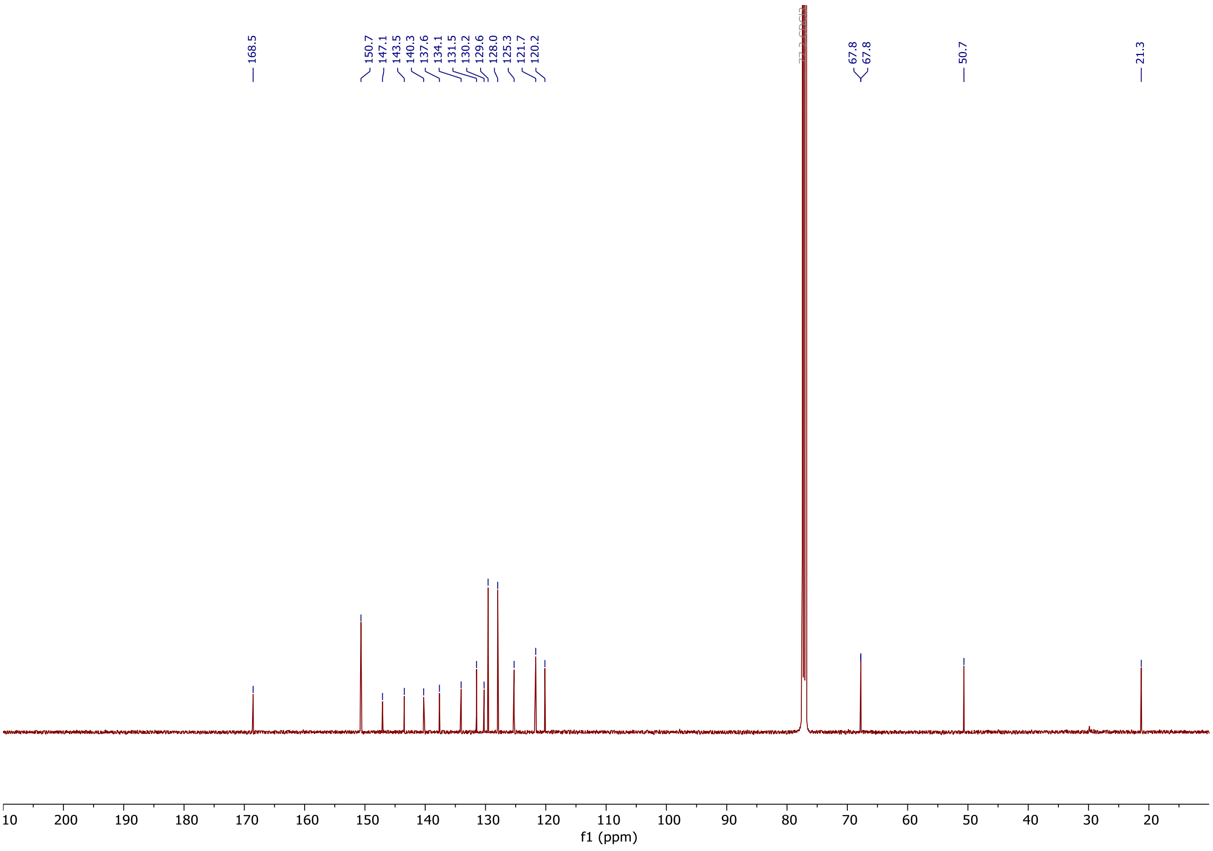
168.5
150.7
147.1
143.5
140.3
137.6
134.1
131.5
130.2
129.6
128.0
125.3
121.7
120.2
67.8
67.8
50.7
21.3
f1 (ppm)

3704

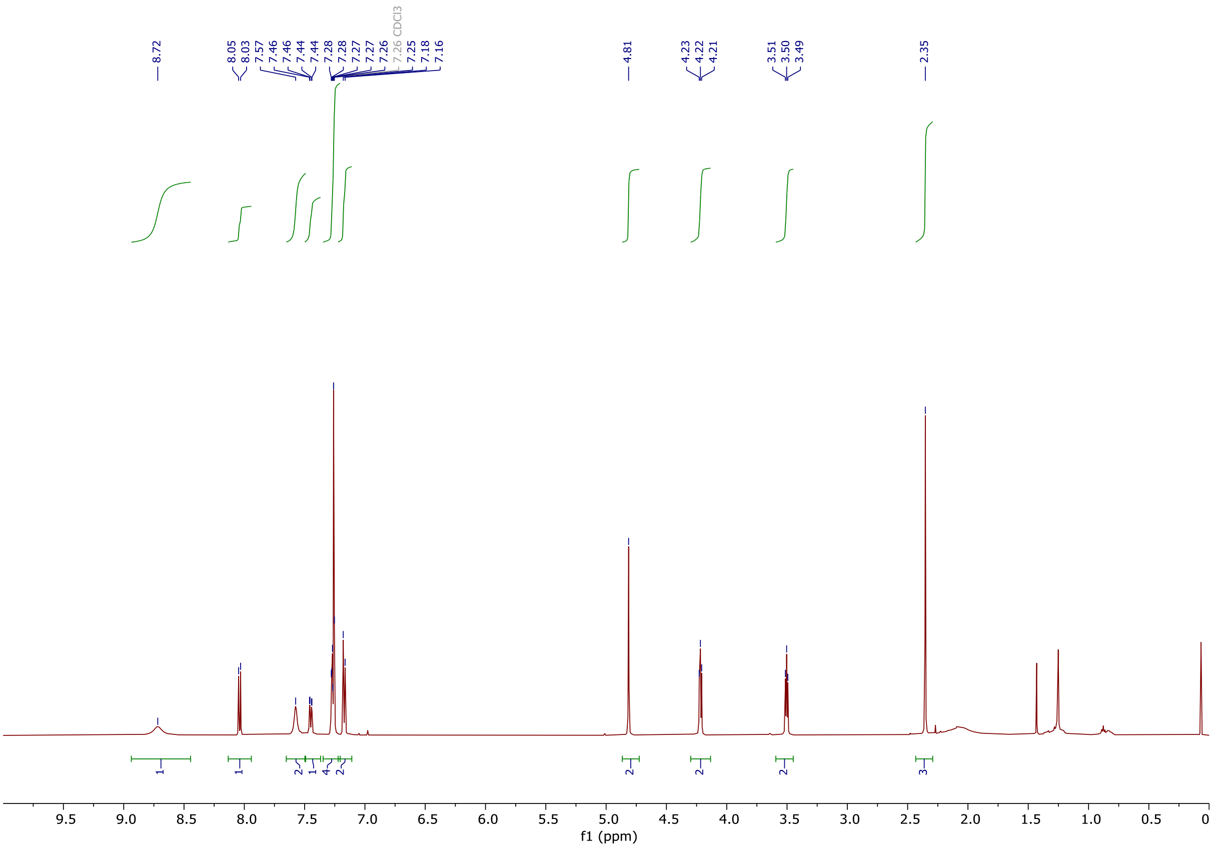

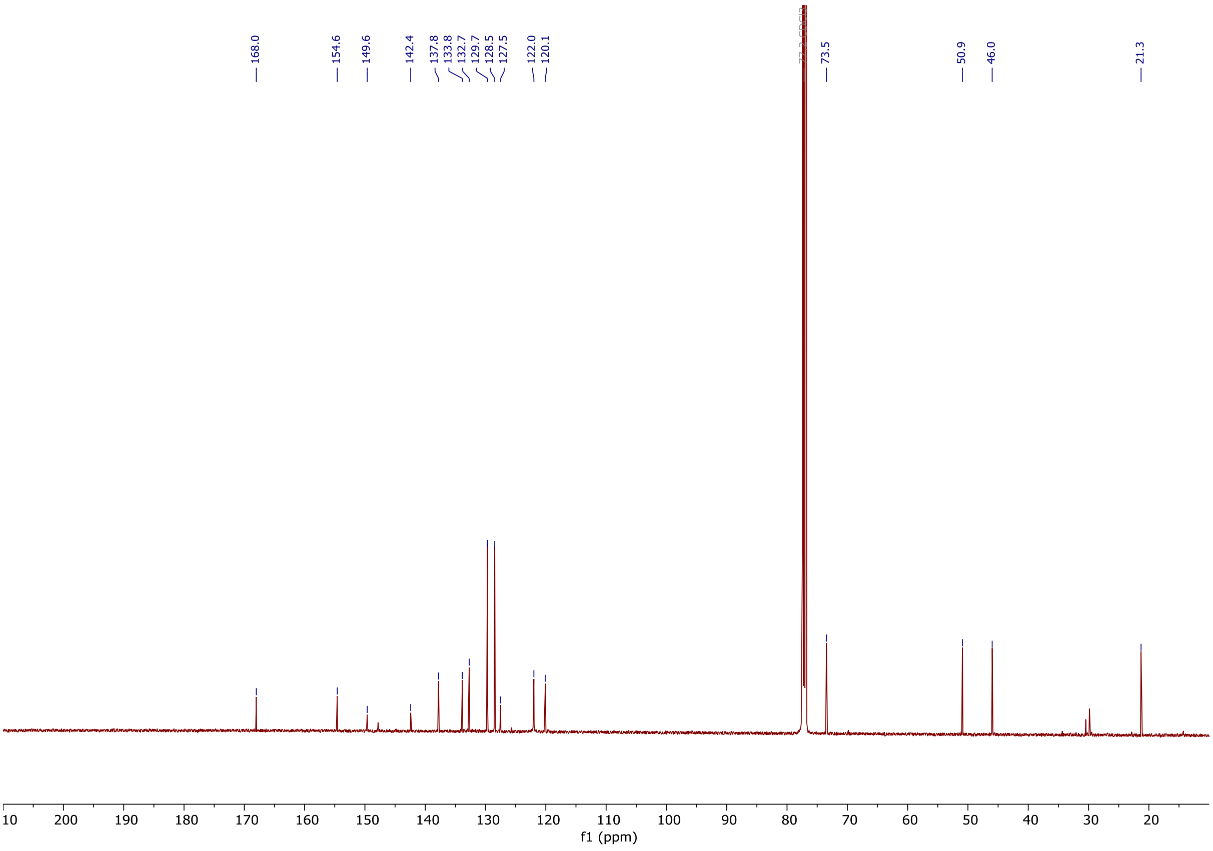

3707

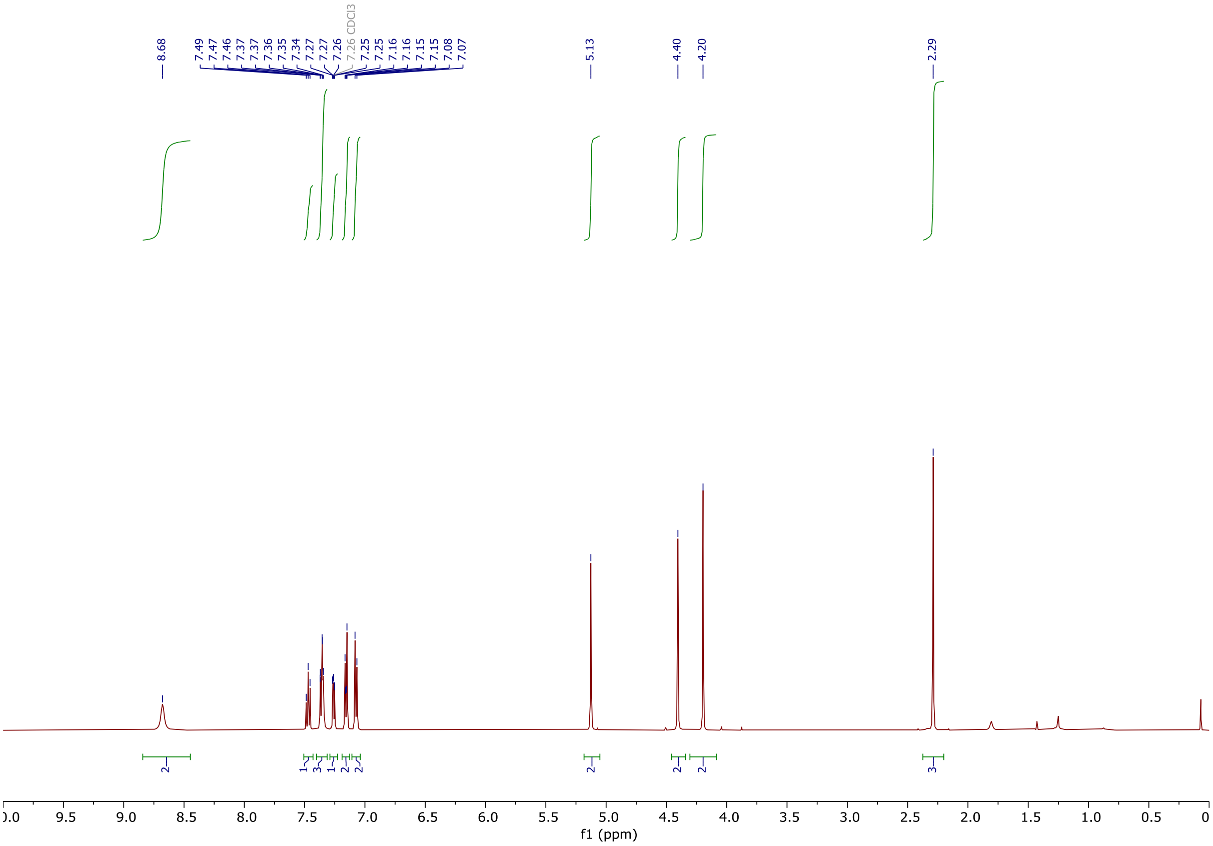
8.68
7.49
7.47
7.46
7.37
7.37
7.36
7.35
7.34
7.27
7.27
7.26
7.26 CDCl3
7.25
7.25
7.16
7.16
7.15
7.15
7.08
7.07
5.13
4.40
4.20
2.29
2
1
3
1
2
2
2
2
2
3
f1 (ppm)

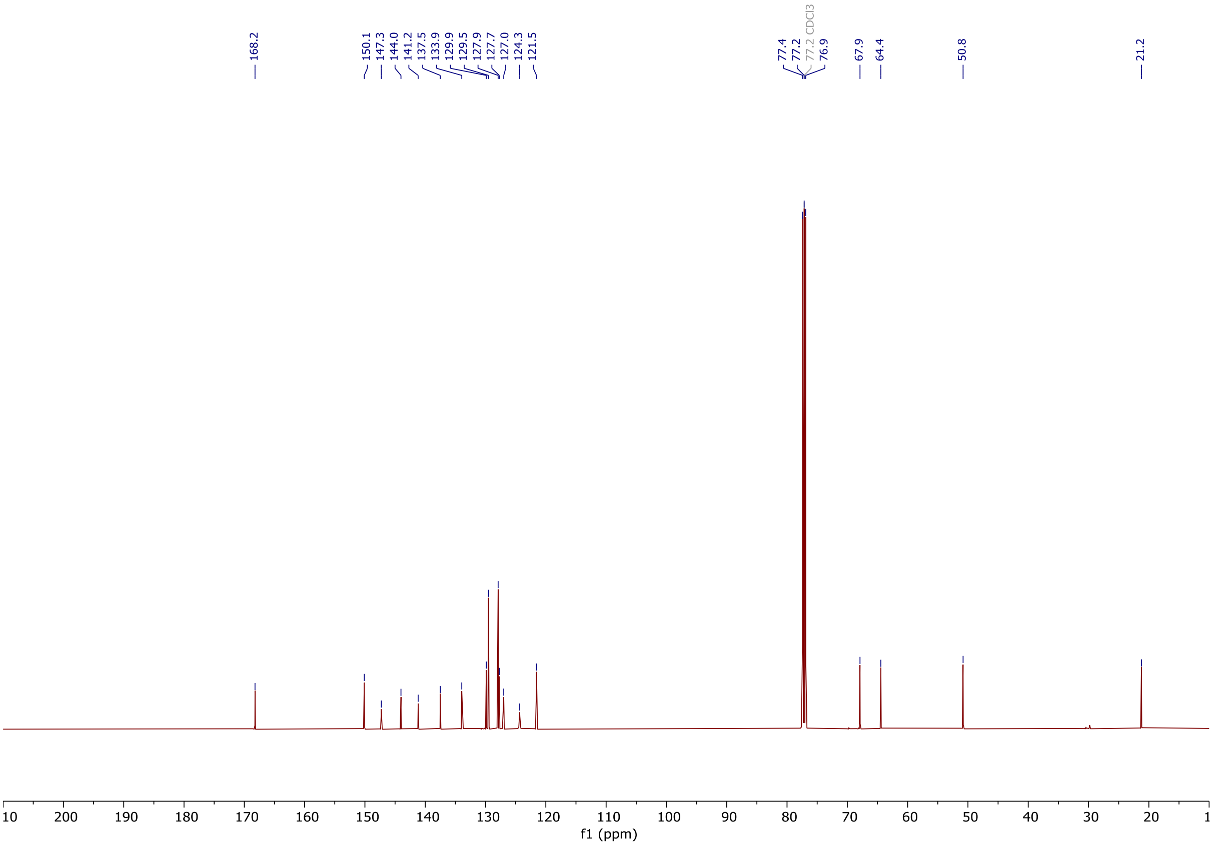
168.2
150.1
147.3
144.0
141.2
137.5
133.9
129.9
129.5
127.9
127.7
127.0
124.3
121.5
77.4
77.2
77.2 CDCl3
76.9
67.9
64.4
50.8
21.2
f1 (ppm)

3710

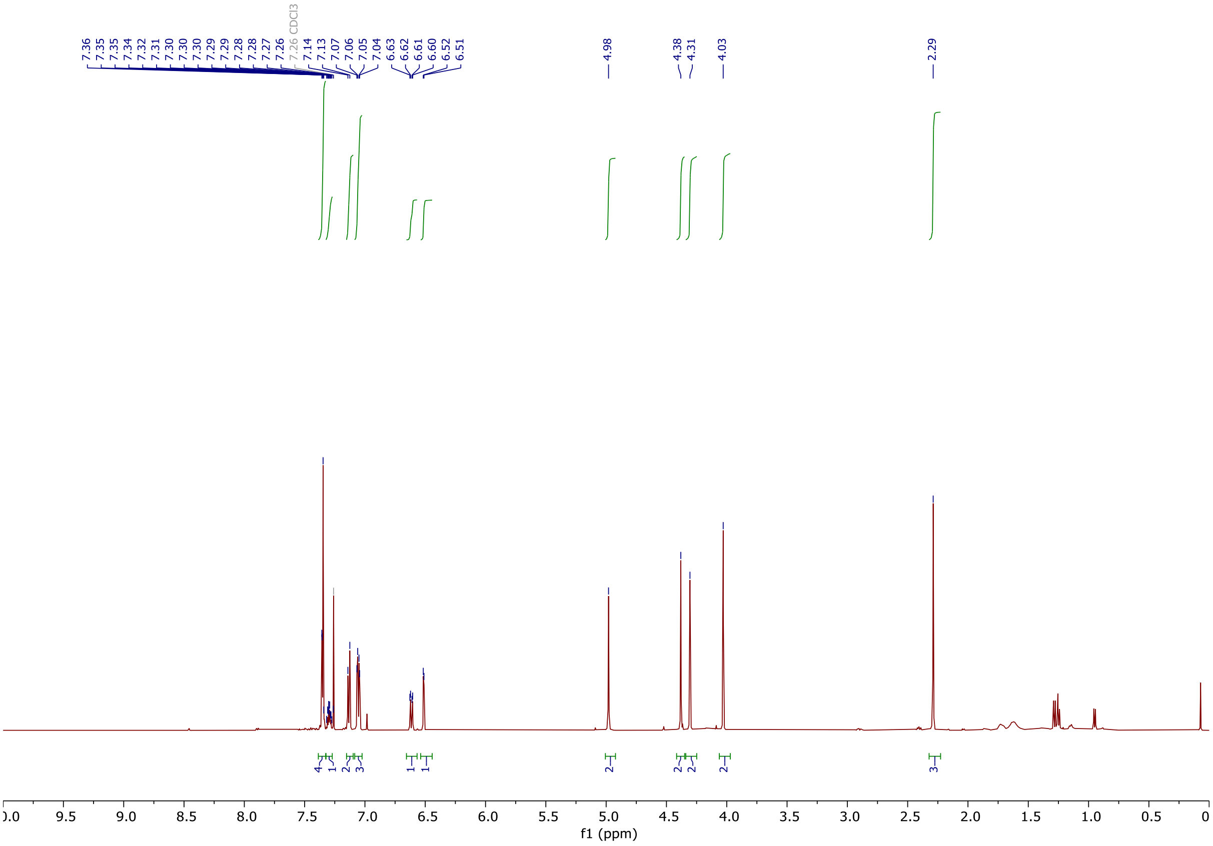

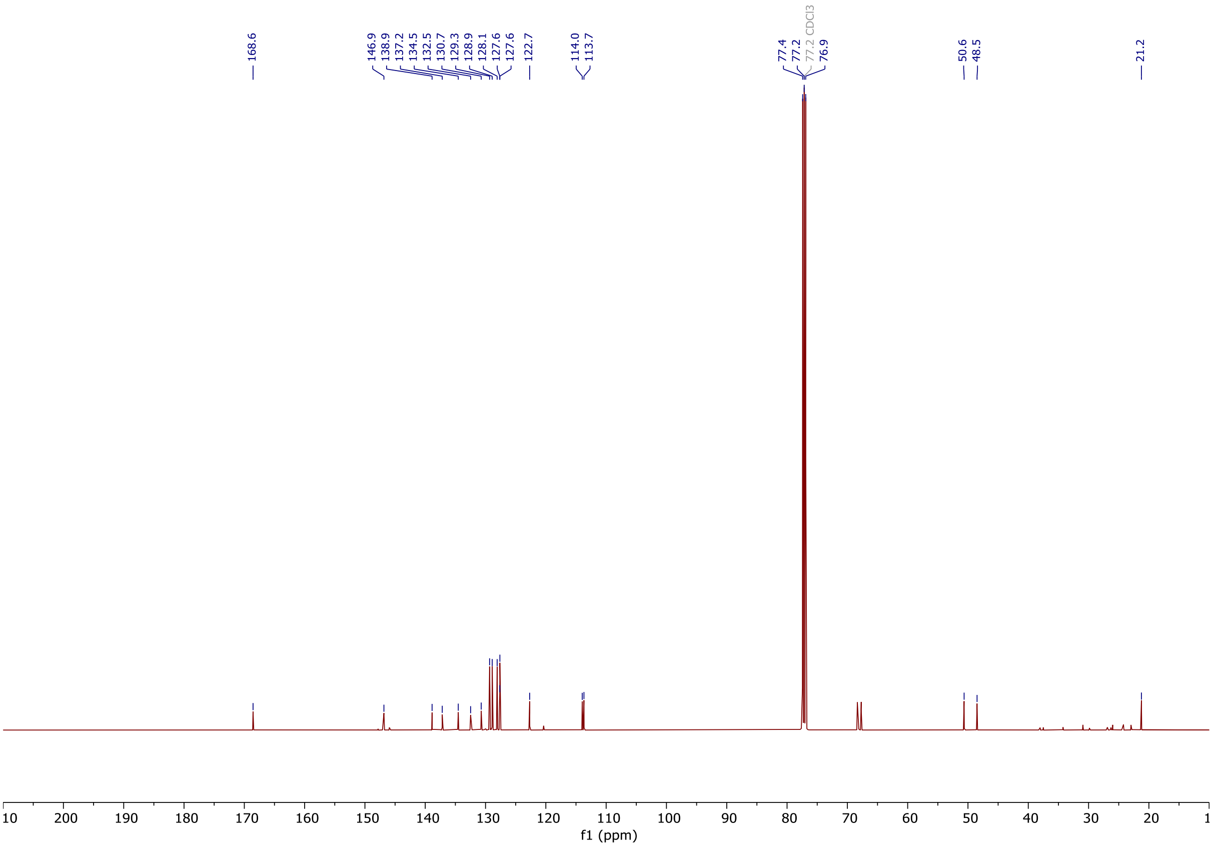

3824

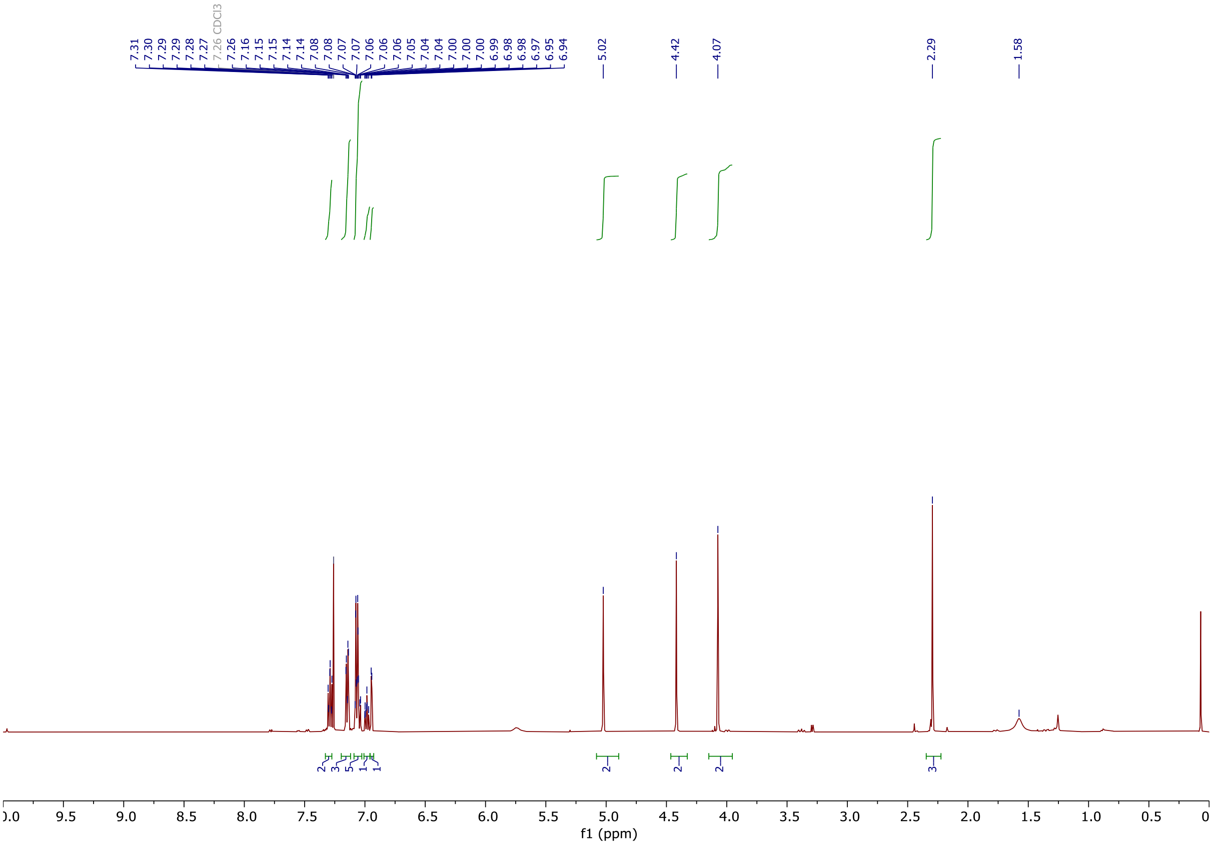
7.31
7.30
7.29
7.29
7.28
7.27
7.26 CDCl3
7.26
7.16
7.15
7.15
7.14
7.14
7.08
7.07
7.07
7.06
7.06
7.05
7.04
7.04
7.00
7.00
7.00
6.99
6.98
6.98
6.97
6.95
6.94
5.02
4.42
4.07
2.29
1.58
2
3
5
1
1
2
2
2
3
f1 (ppm)

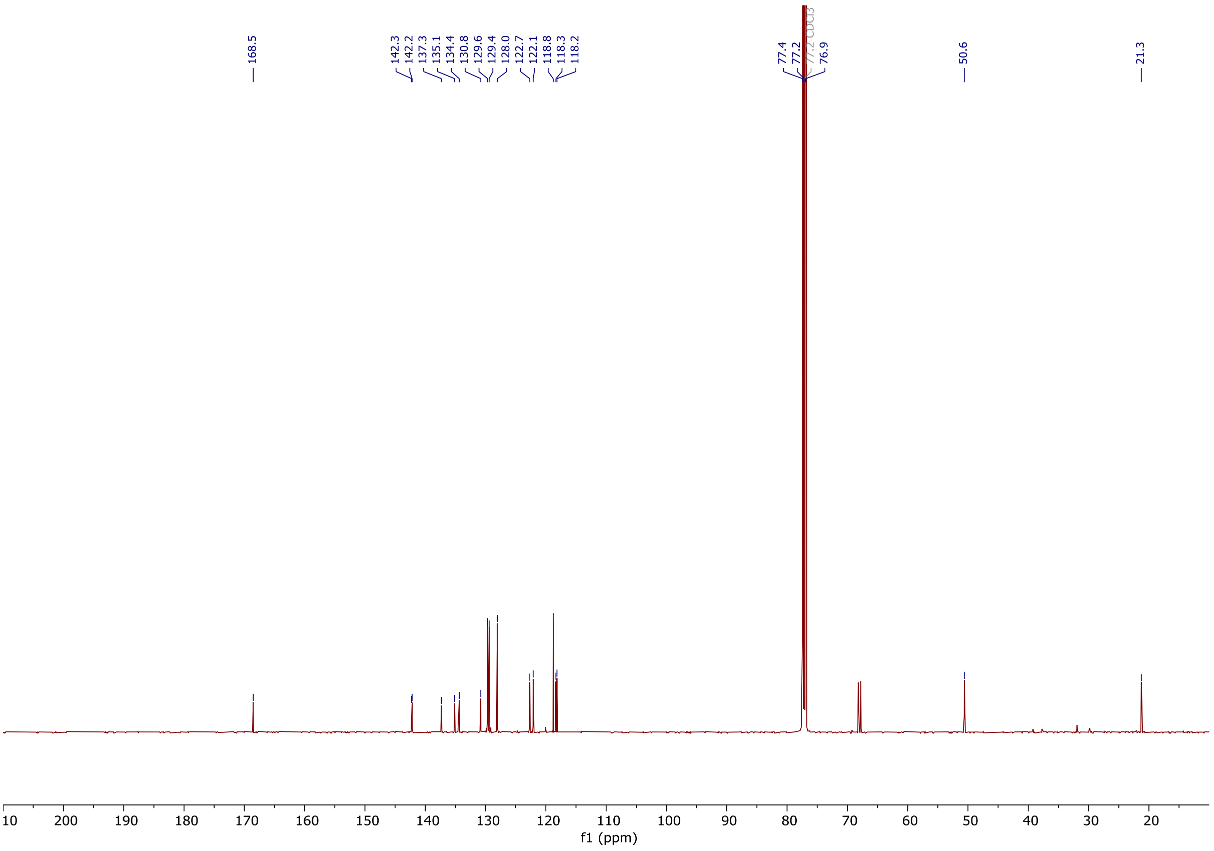
168.5
142.3
142.2
137.3
135.1
134.4
130.8
129.6
129.4
128.0
122.7
122.1
118.8
118.3
118.2
77.4
77.2
77.2 CDCl3
76.9
50.6
21.3
f1 (ppm)

3825

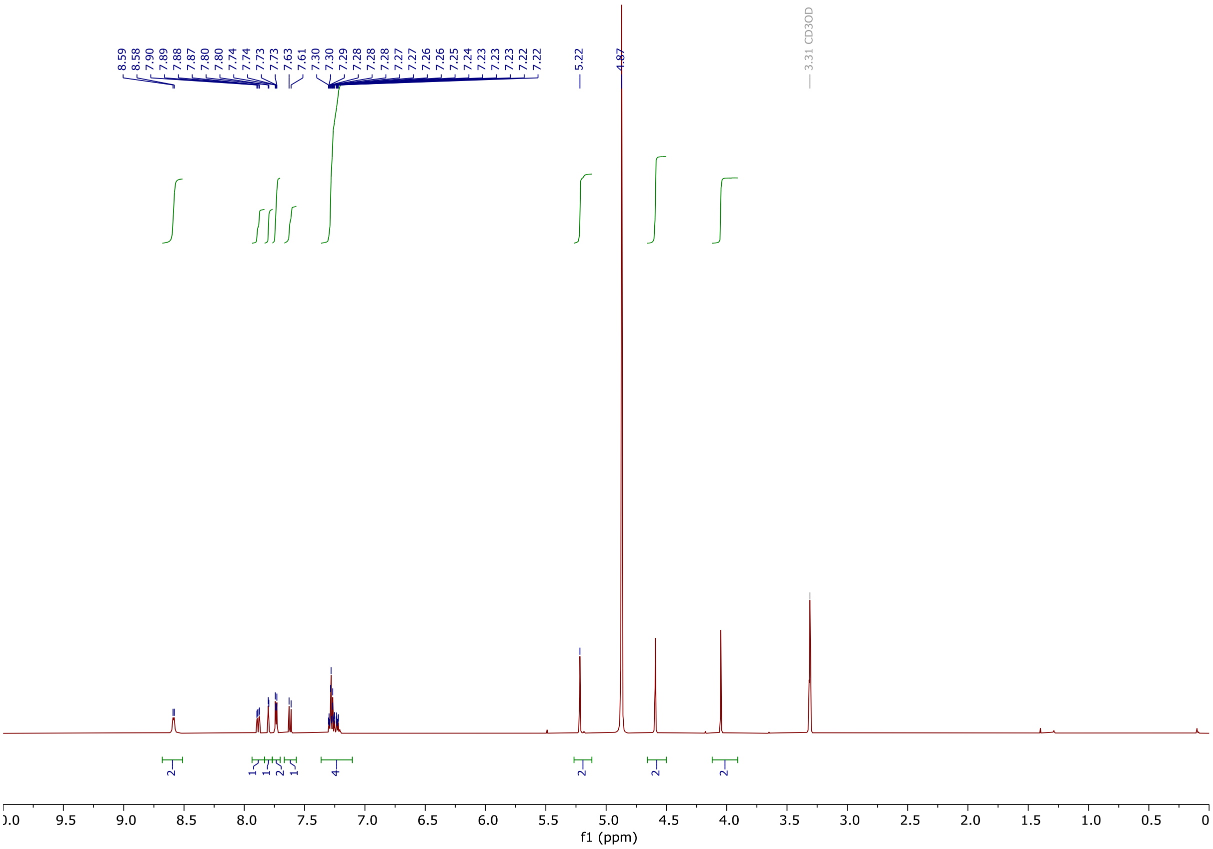
8.59
8.58
7.90
7.89
7.88
7.87
7.80
7.80
7.74
7.73
7.73
7.63
7.61
7.30
7.29
7.28
7.28
7.27
7.27
7.26
7.26
7.25
7.24
7.23
7.23
7.23
7.22
7.22
5.22
4.87
3.31 CD3OD
2
1
1
2
1
4
2
2
2
f1 (ppm)

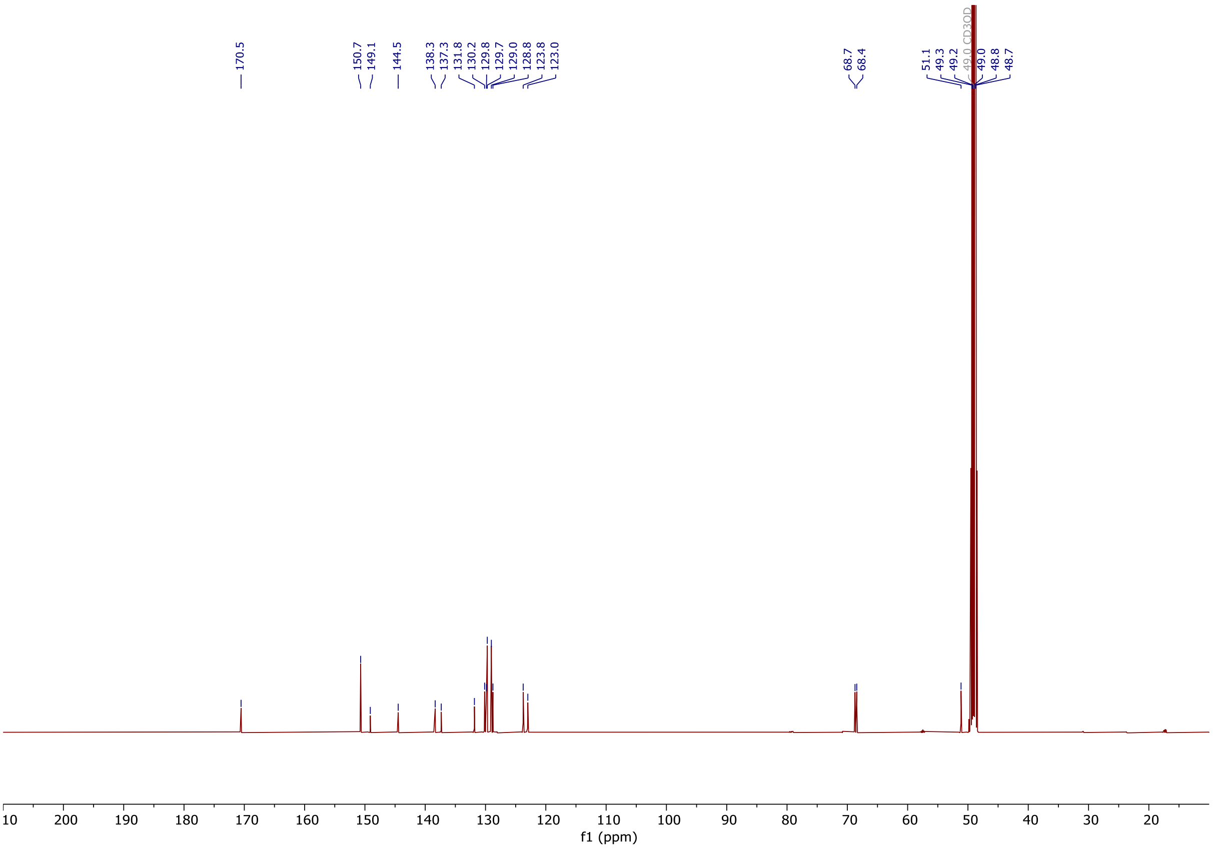
170.5
150.7
149.1
144.5
138.3
137.3
131.8
130.2
129.8
129.7
129.0
128.8
123.8
123.0
68.7
68.4
51.1
49.3
49.2
49.0 CD3OD
49.0
48.8
48.7
f1 (ppm)

4115

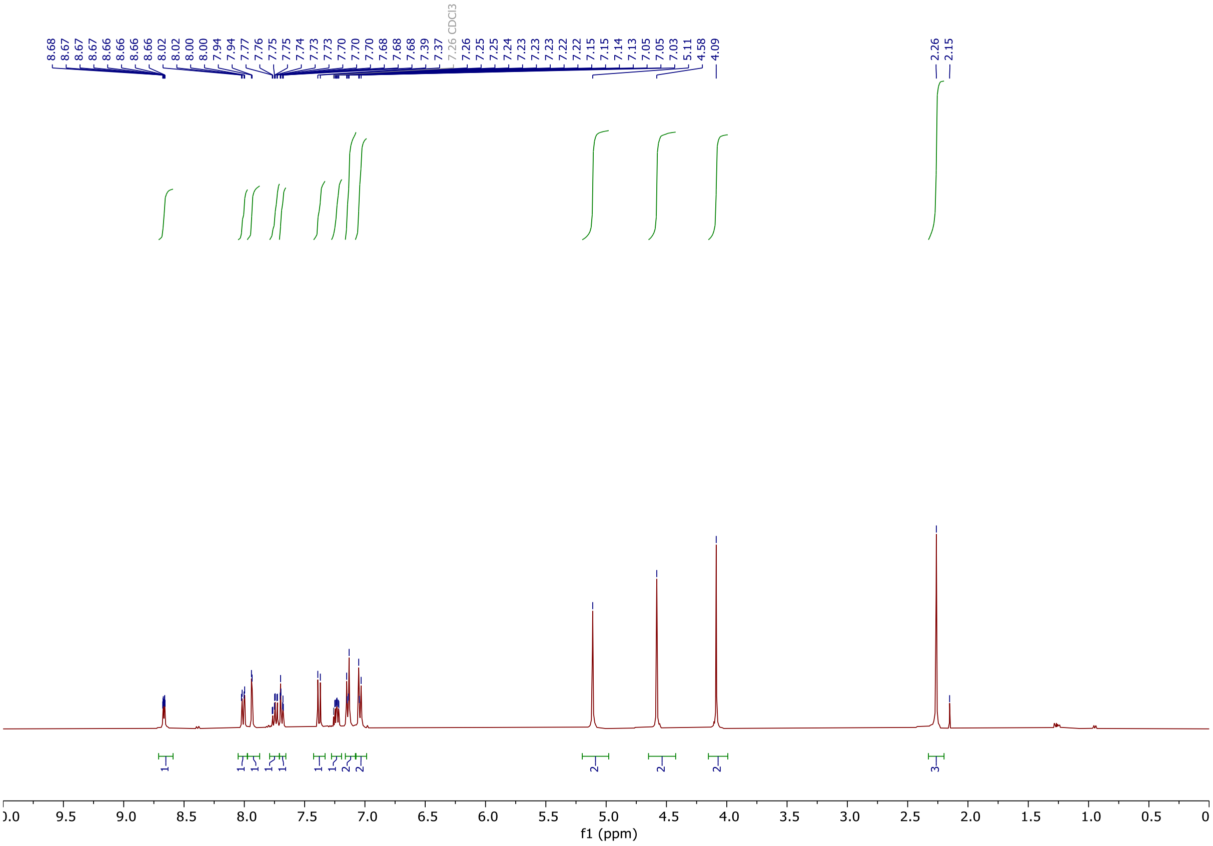
8.68
8.67
8.67
8.66
8.66
8.66
8.66
8.02
8.02
8.00
8.00
7.94
7.77
7.76
7.75
7.75
7.74
7.73
7.73
7.70
7.70
7.70
7.68
7.68
7.68
7.39
7.37
7.26 CDCl3
7.26
7.25
7.25
7.24
7.23
7.23
7.23
7.22
7.22
7.15
7.15
7.14
7.13
7.05
7.05
7.03
5.11
4.58
4.09
2.26
2.15
f1 (ppm)

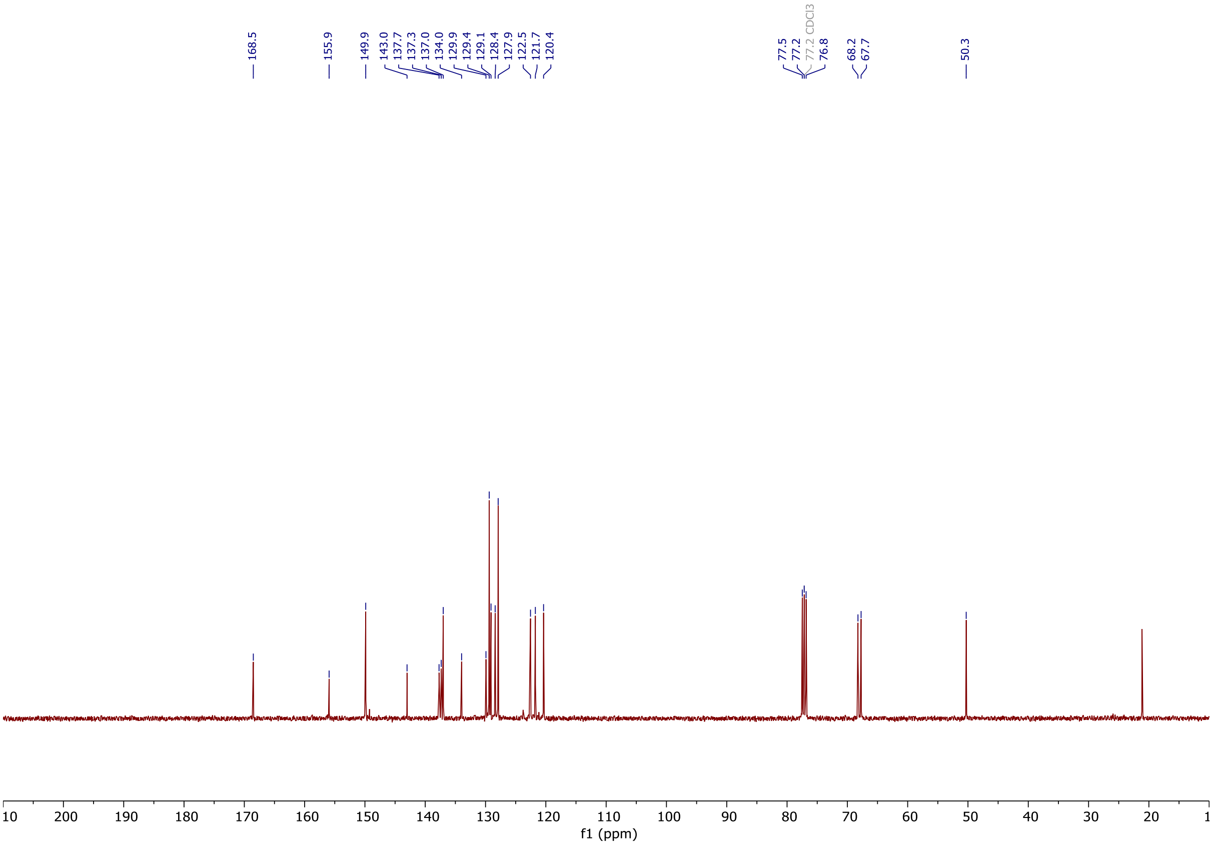
168.5
155.9
149.9
143.0
137.7
137.3
137.0
134.0
129.9
129.4
129.1
128.4
127.9
122.5
121.7
120.4
77.5
77.2
77.2 CDCl3
76.8
68.2
67.7
50.3
f1 (ppm)

4116

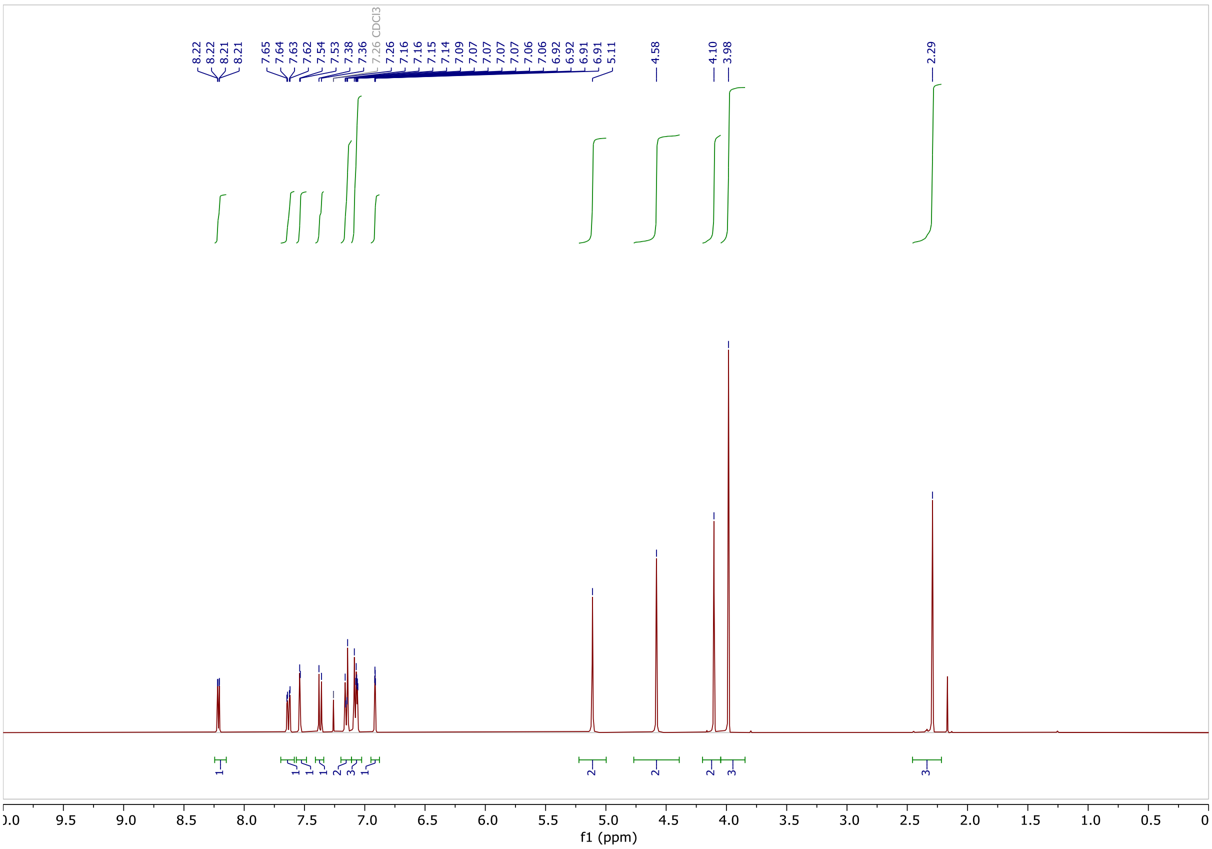
8.22
8.22
8.21
8.21
7.65
7.64
7.63
7.62
7.54
7.53
7.38
7.36
7.26 CDCl3
7.26
7.16
7.16
7.15
7.14
7.09
7.07
7.07
7.07
7.07
7.06
6.92
6.92
6.91
6.91
5.11
4.58
4.10
3.98
2.29
f1 (ppm)

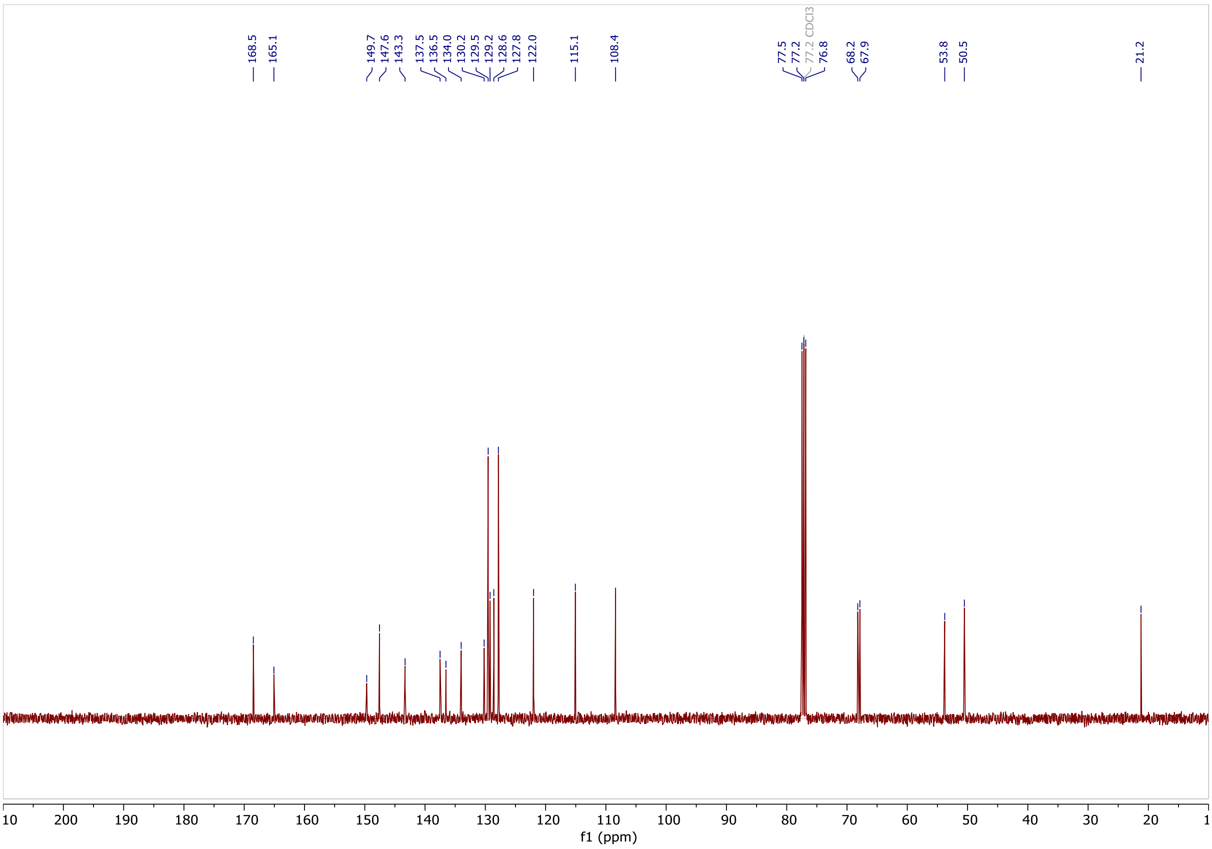
168.5
165.1
149.7
147.6
143.3
137.5
136.5
134.0
130.2
129.5
129.2
128.6
127.8
122.0
115.1
108.4
77.5
77.2
77.2 CDCl3
76.8
68.2
67.9
53.8
50.5
21.2
f1 (ppm)

4117

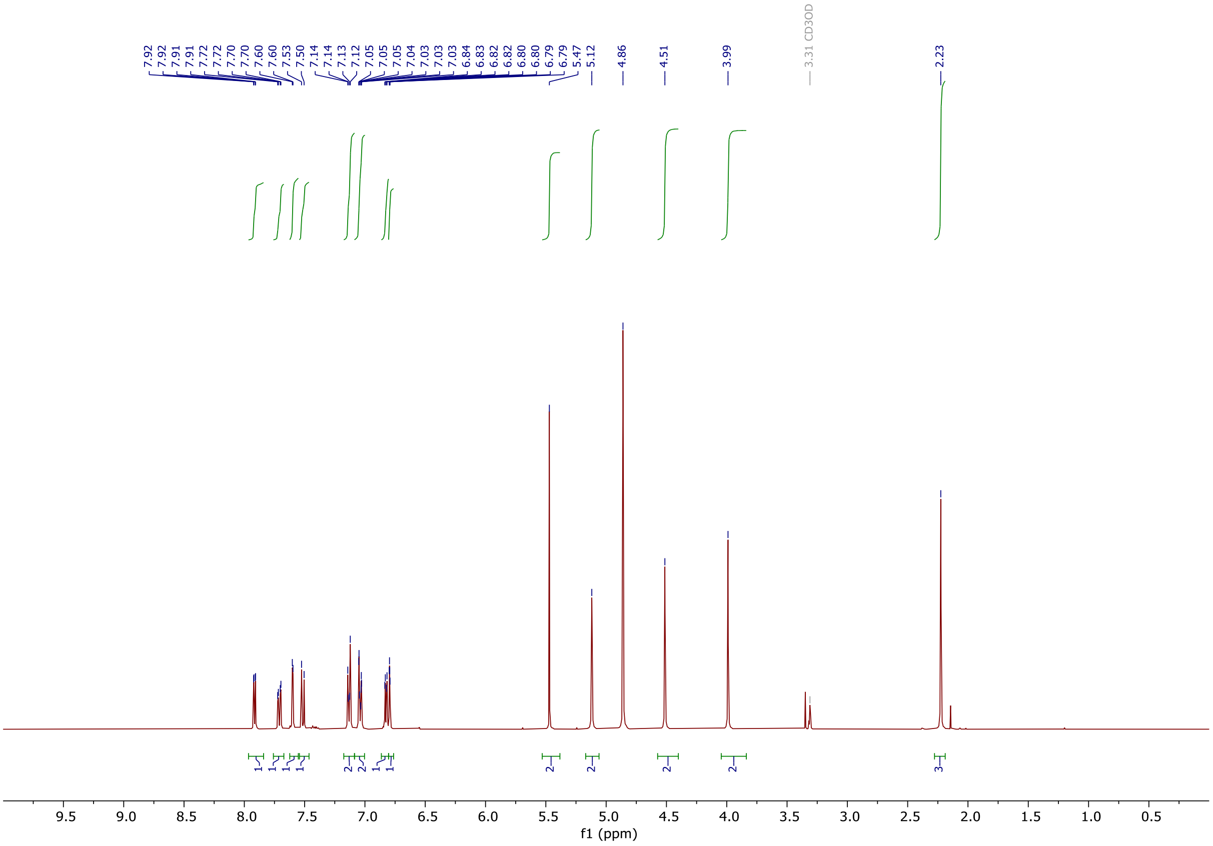

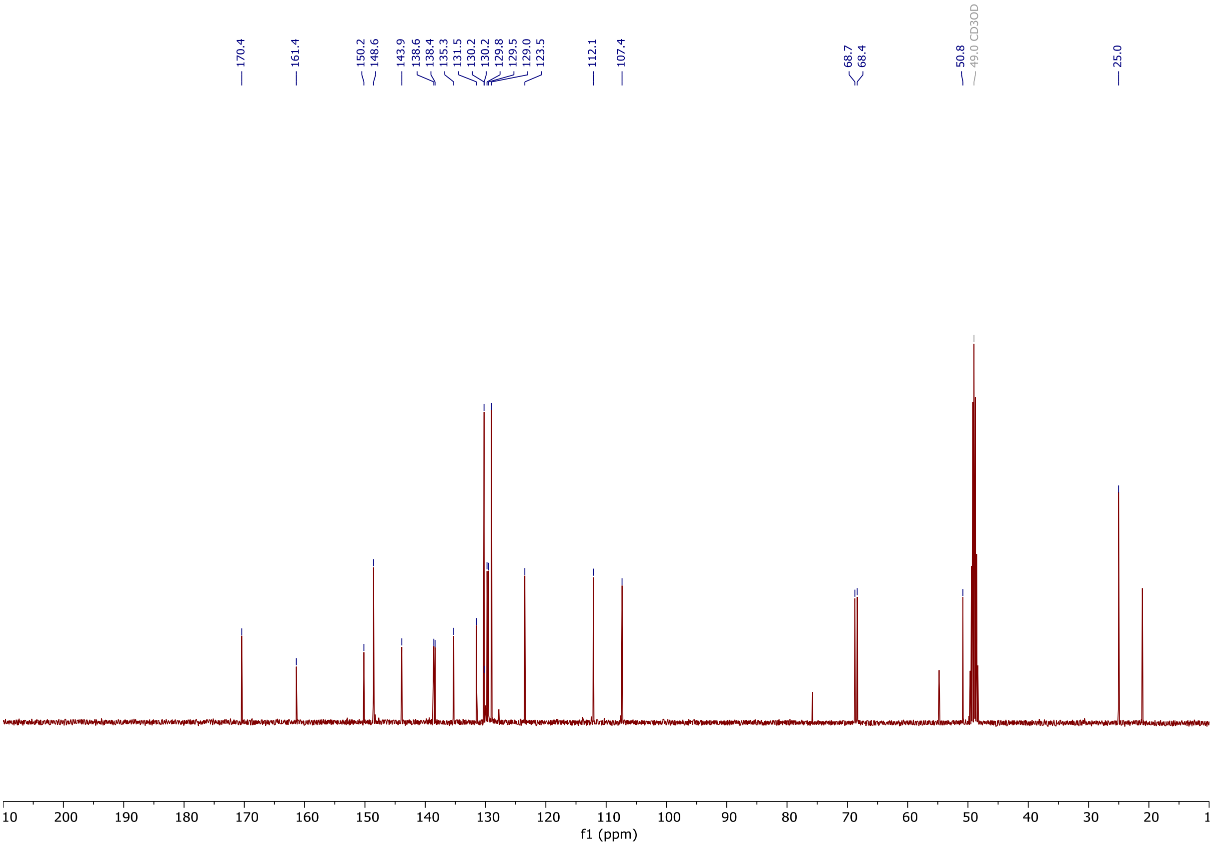

4118

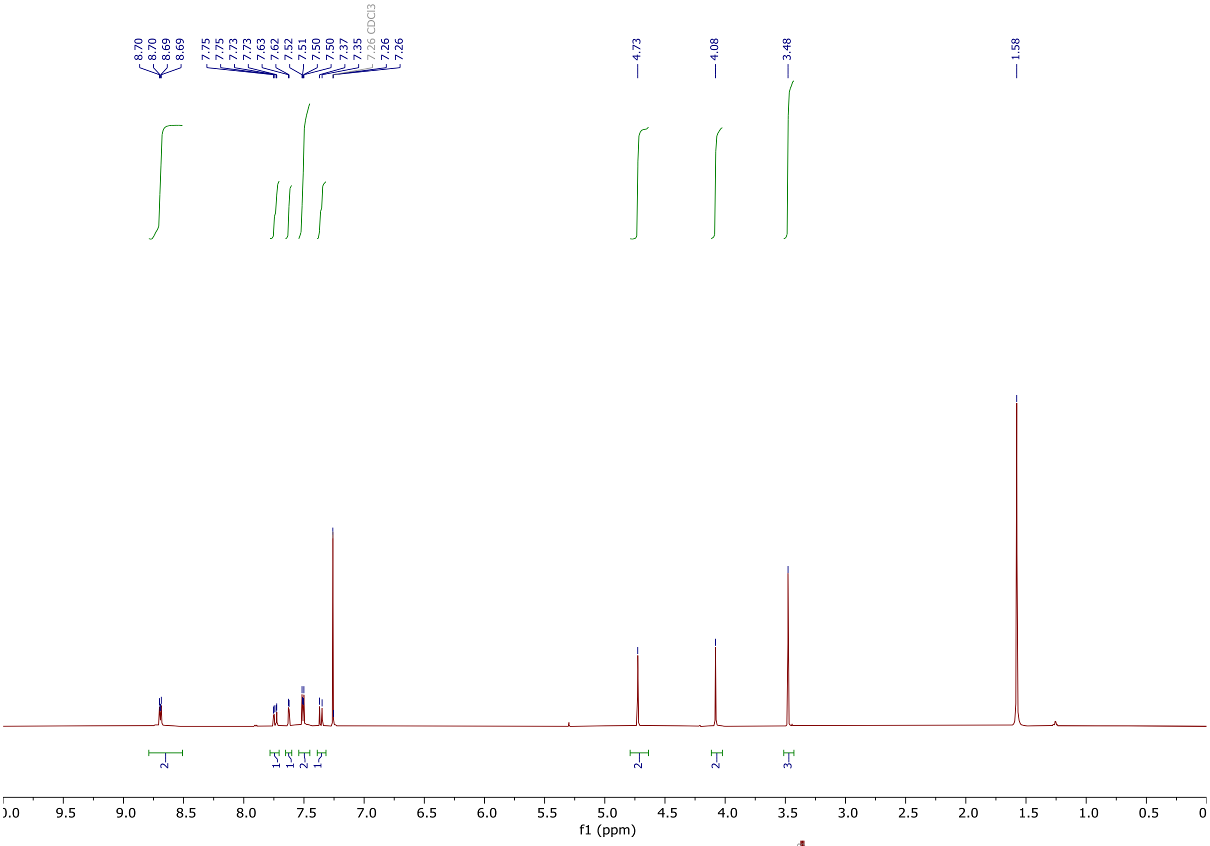

8.70
8.70
8.69
8.69
7.75
7.75
7.73
7.73
7.63
7.62
7.52
7.51
7.50
7.50
7.37
7.35
7.26 CDCl3
7.26
7.26
4.73
4.08
3.48
1.58
f1 (ppm)

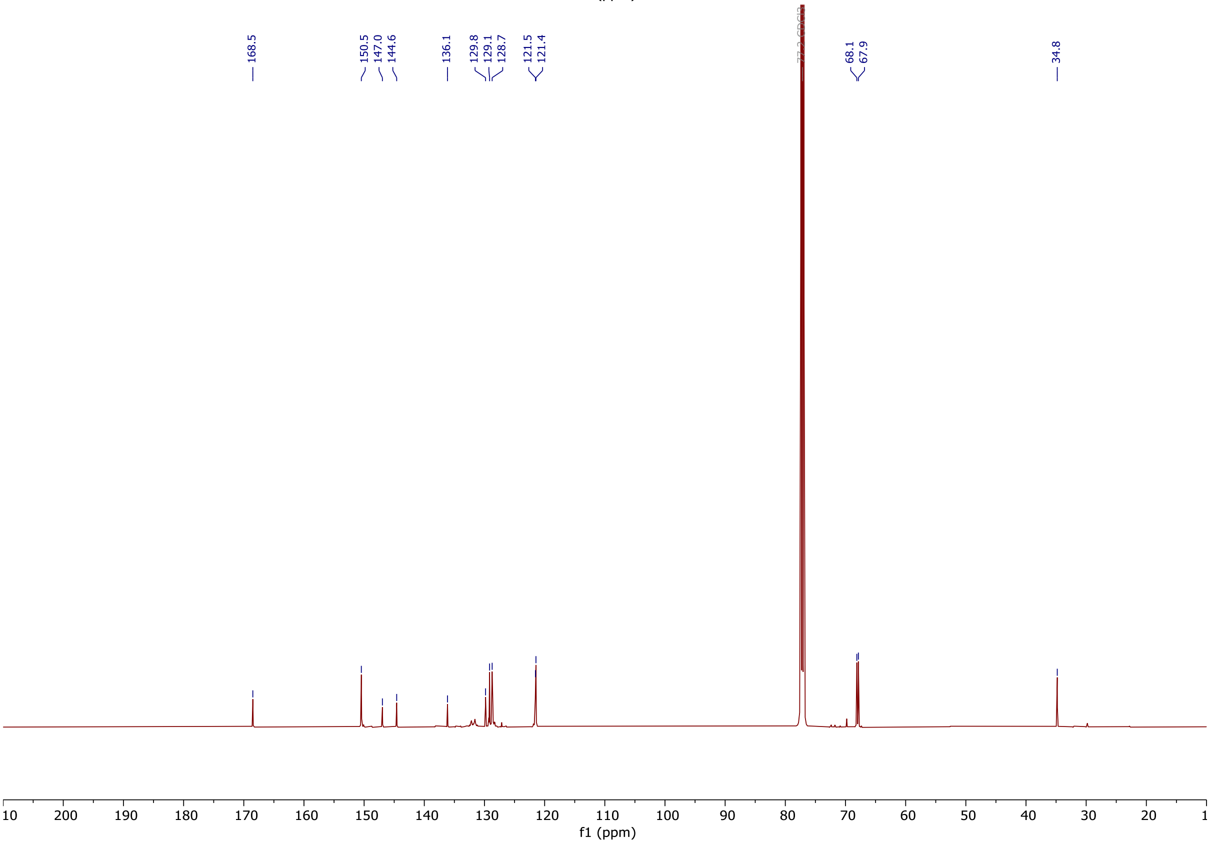

168.5
150.5
147.0
144.6
136.1
129.8
129.1
128.7
121.5
121.4
68.1
67.9
34.8
f1 (ppm)

4120

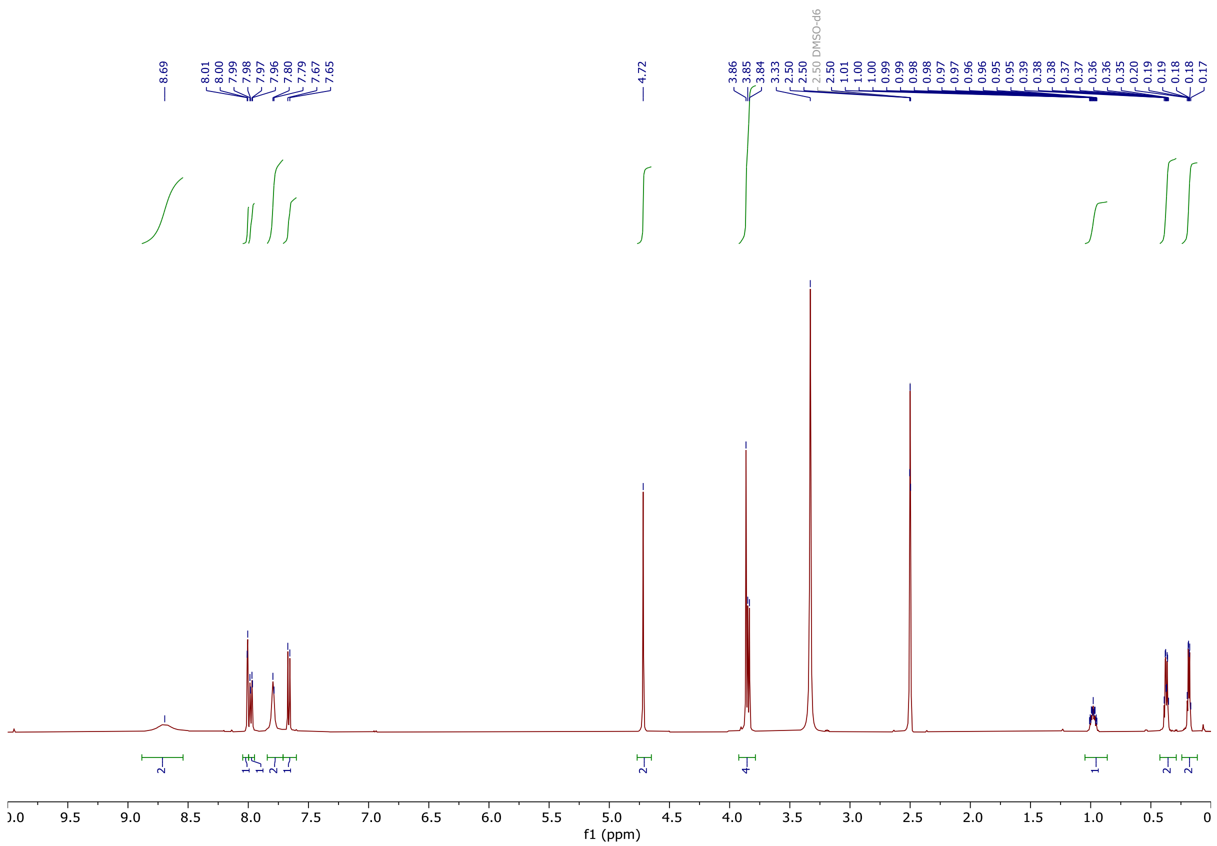

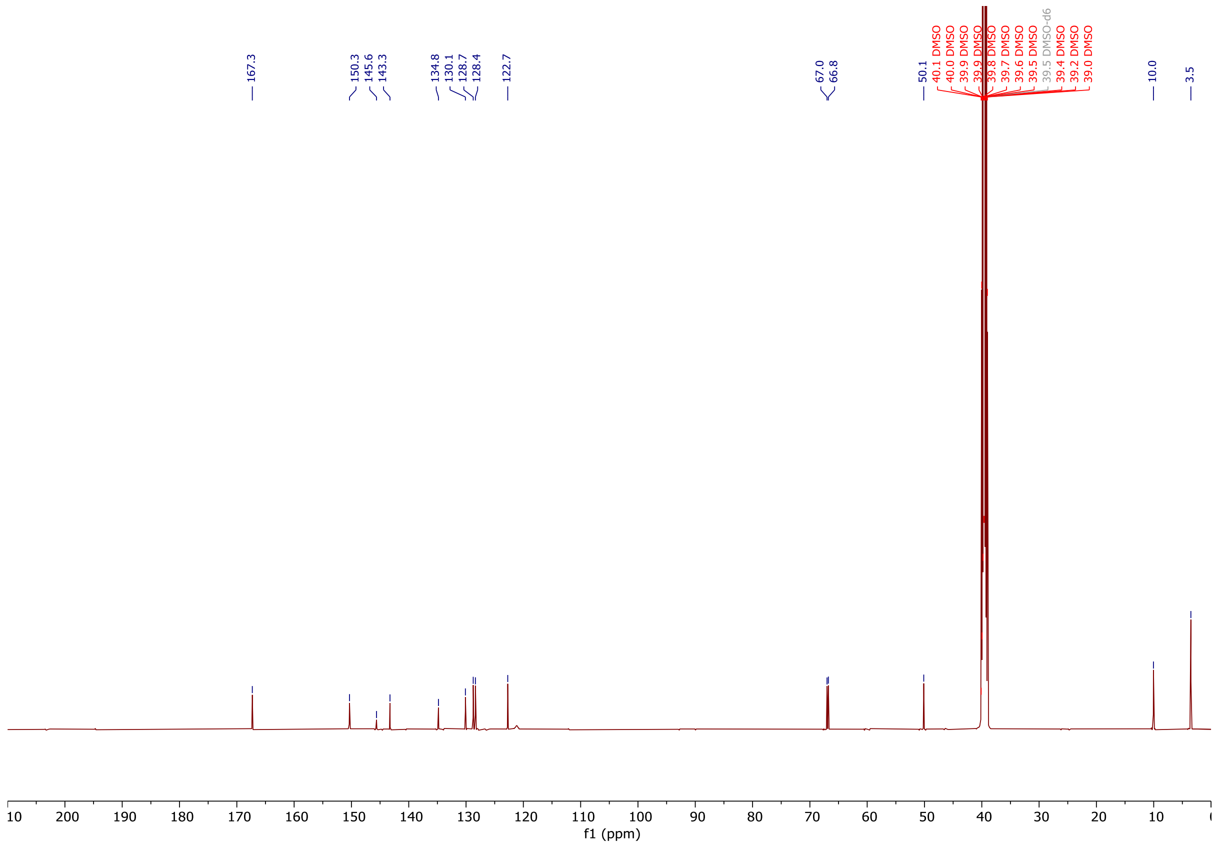

4122

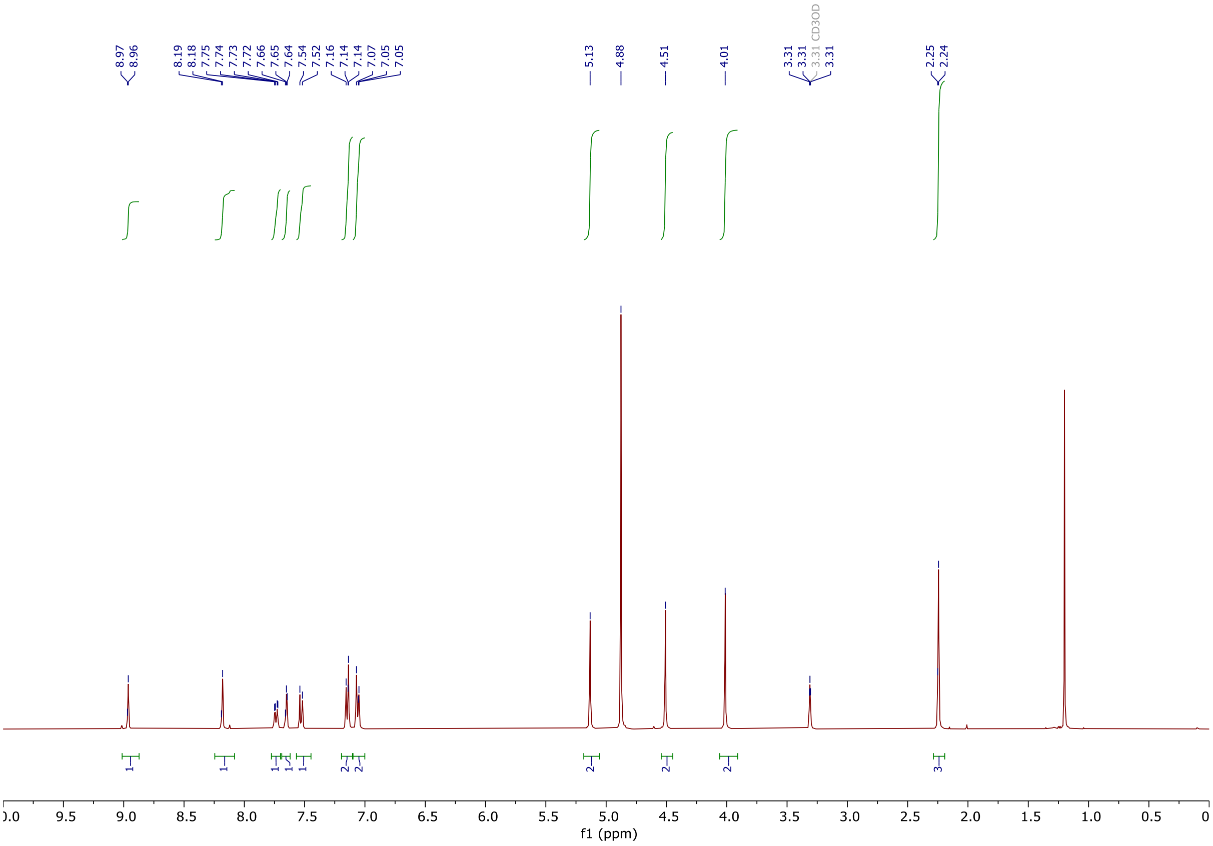

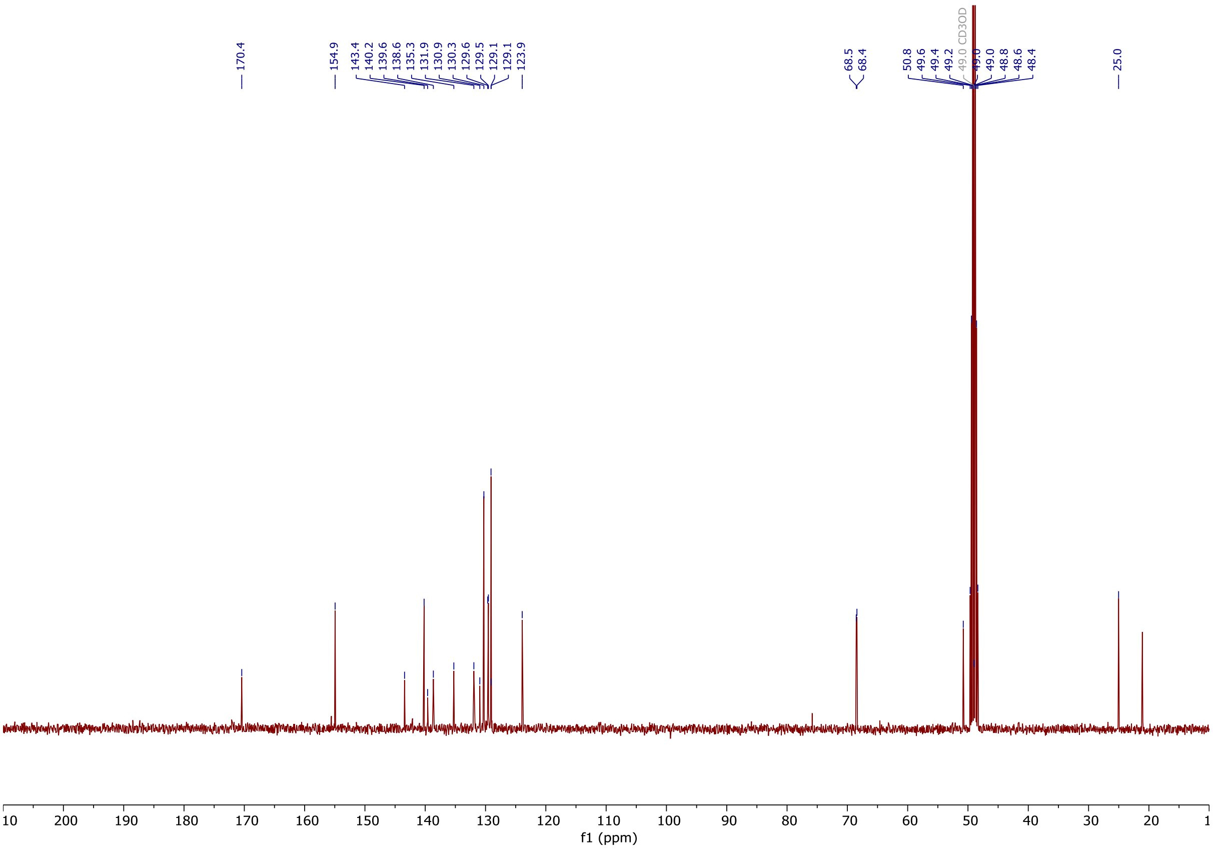

4123

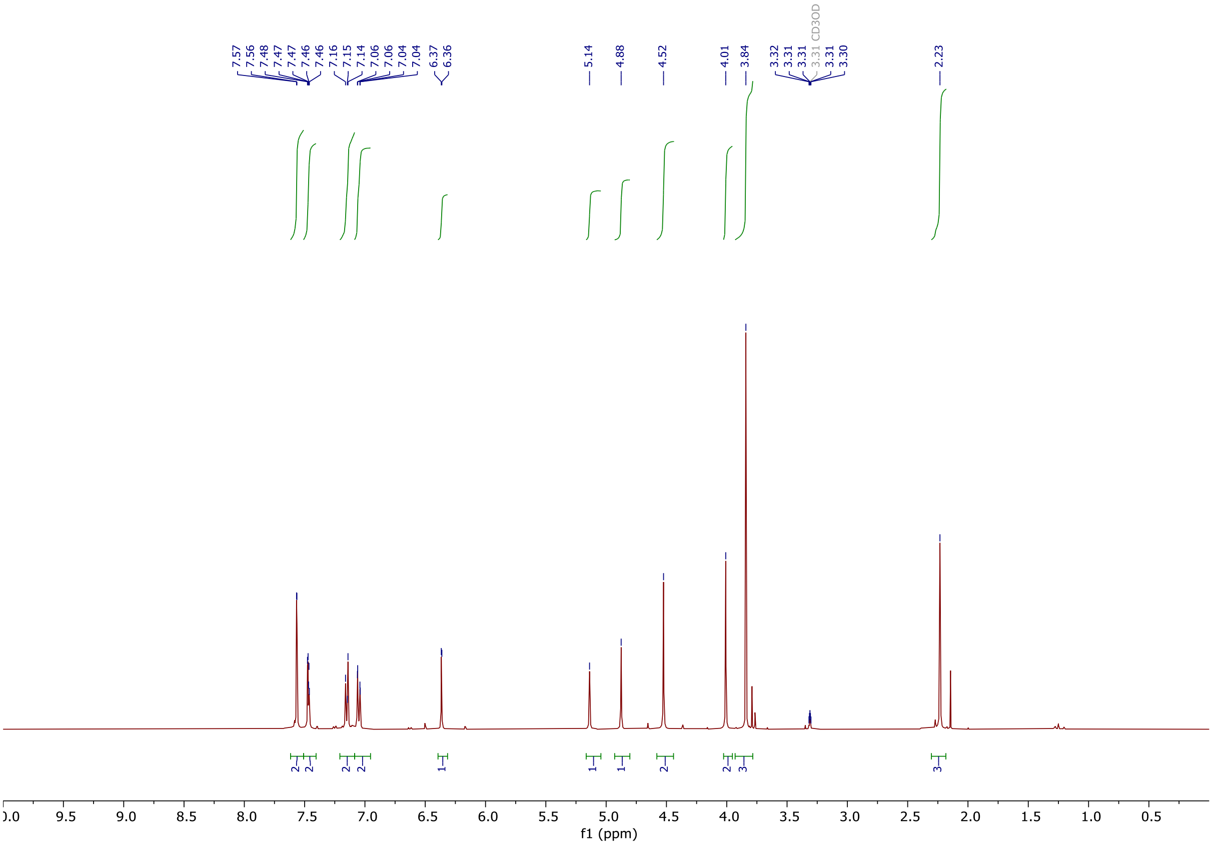
7.57 7.56 7.48 7.47 7.47 7.46 7.46 7.16 7.15 7.14 7.06 7.06 7.04 7.04 6.37 6.36
5.14
4.88
4.52
4.01
3.84
3.32 3.31 3.31 3.31 CD3OD 3.31 3.30
2.23
f1 (ppm)

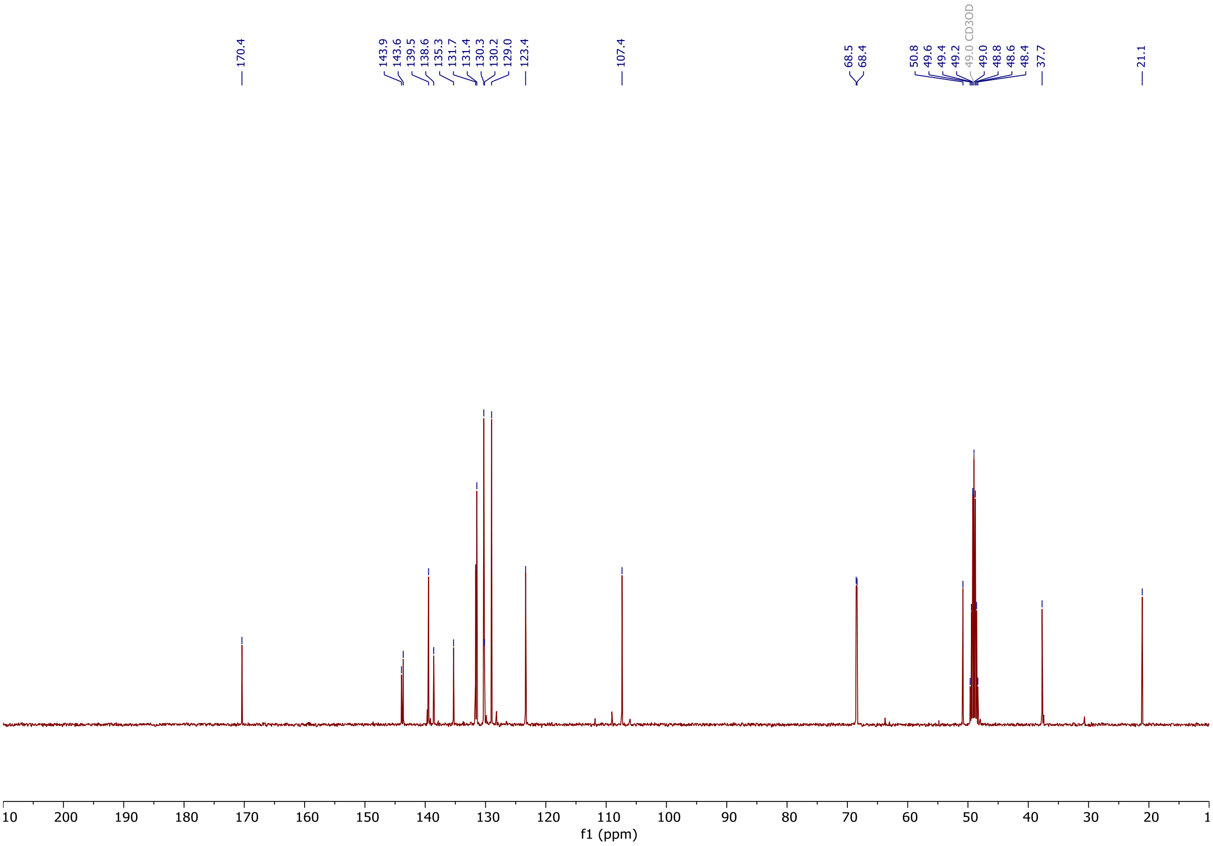
170.4
143.9 143.6 139.5 138.6 135.3 131.7 131.4 130.3 130.2 129.0 123.4
107.4
68.5 68.4
50.8 49.6 49.4 49.2 49.0 CD3OD 49.0 48.8 48.6 48.4 37.7
21.1
f1 (ppm)

3699

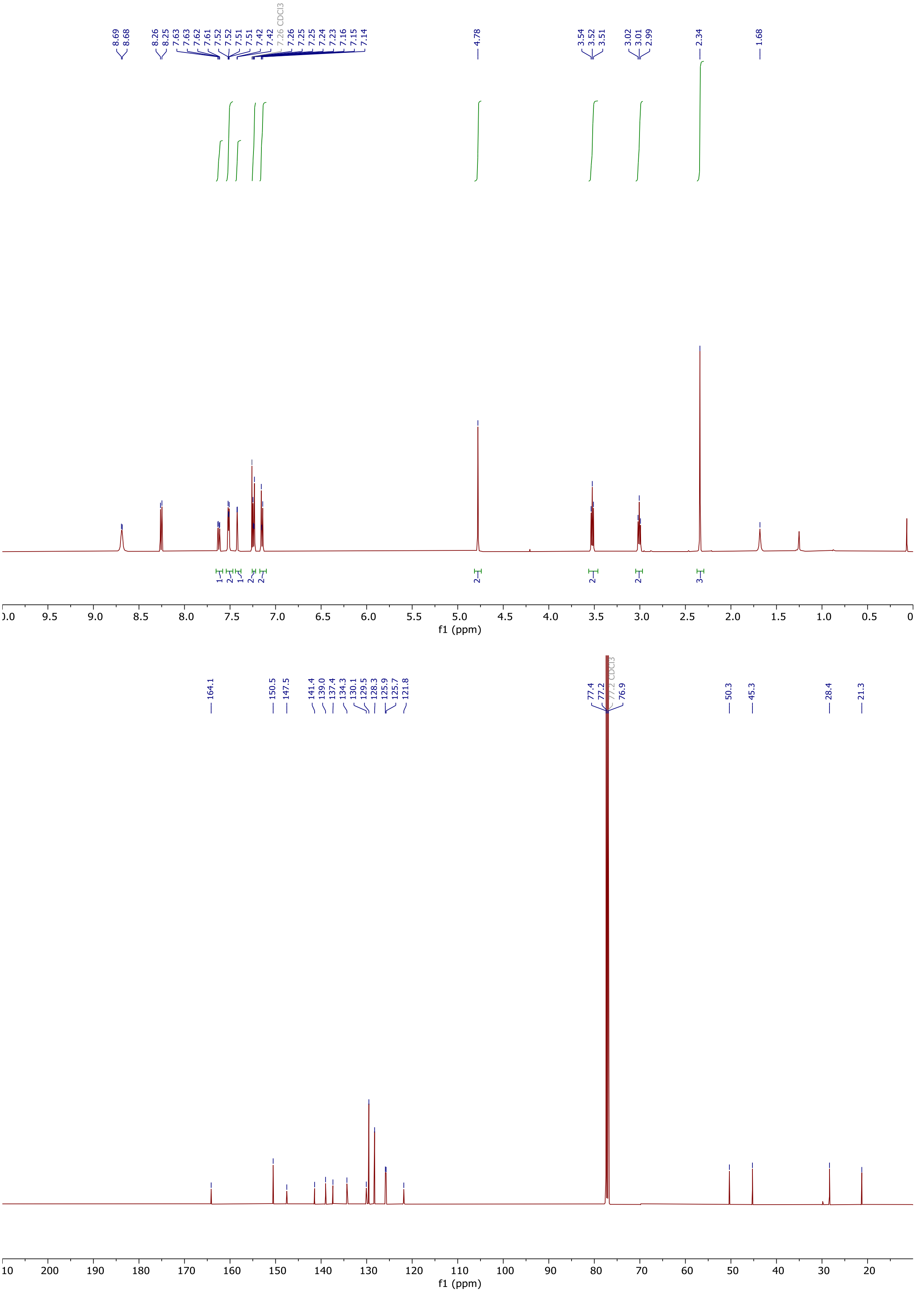

8.69
8.68
8.26
8.25
7.63
7.63
7.62
7.61
7.52
7.52
7.51
7.51
7.42
7.42
7.26 CDCl3
7.26
7.25
7.25
7.24
7.23
7.16
7.15
7.14
4.78
3.54
3.52
3.51
3.02
3.01
2.99
2.34
1.68
1
2
1
2
2
2
2
2
3
f1 (ppm)
164.1
150.5
147.5
141.4
139.0
137.4
134.3
130.1
129.5
128.3
125.9
125.7
121.8
77.4
77.2
77.2 CDCl3
76.9
50.3
45.3
28.4
21.3
f1 (ppm)

4236

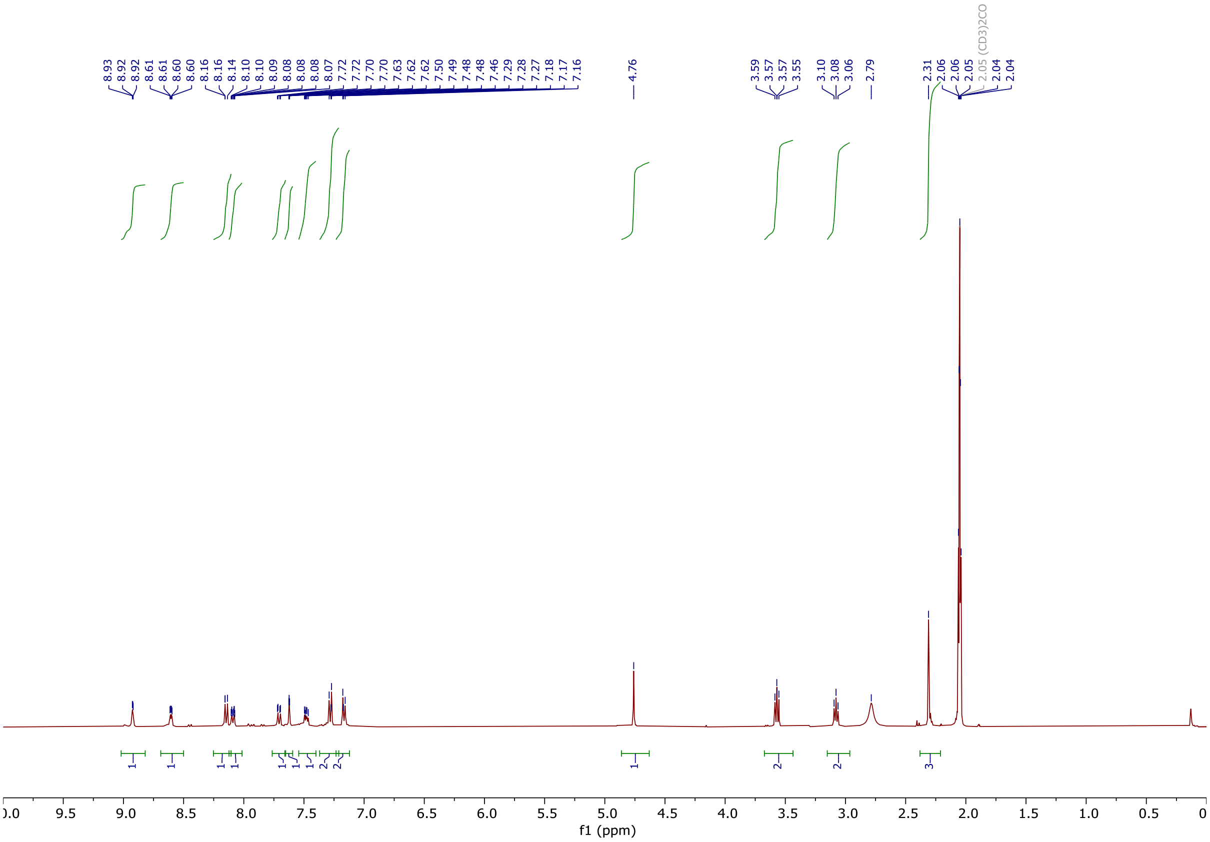
8.93
8.92
8.92
8.61
8.60
8.60
8.16
8.14
8.10
8.10
8.09
8.08
8.08
8.08
8.07
7.72
7.72
7.70
7.63
7.62
7.50
7.49
7.48
7.48
7.46
7.29
7.28
7.27
7.18
7.17
7.16
4.76
3.59
3.57
3.57
3.55
3.10
3.08
3.06
2.79
2.31
2.06
2.06
2.05 (CD3)2CO
2.05
2.04
2.04
1
1
1
1
1
1
1
2
2
1
2
2
3
f1 (ppm)

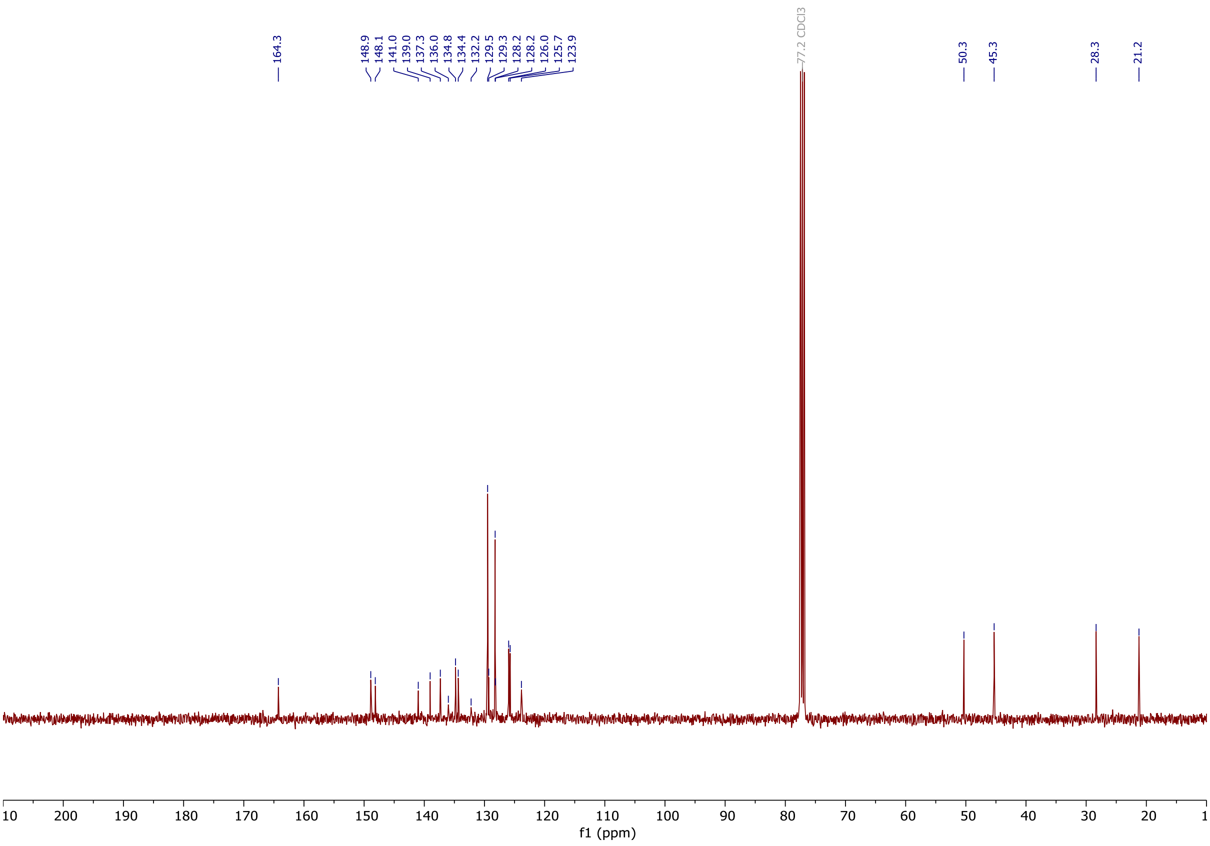
164.3
148.9
148.1
141.0
139.0
137.3
136.0
134.8
134.4
132.2
129.5
129.3
128.2
128.2
126.0
125.7
123.9
77.2 CDCl3
50.3
45.3
28.3
21.2
f1 (ppm)

4237

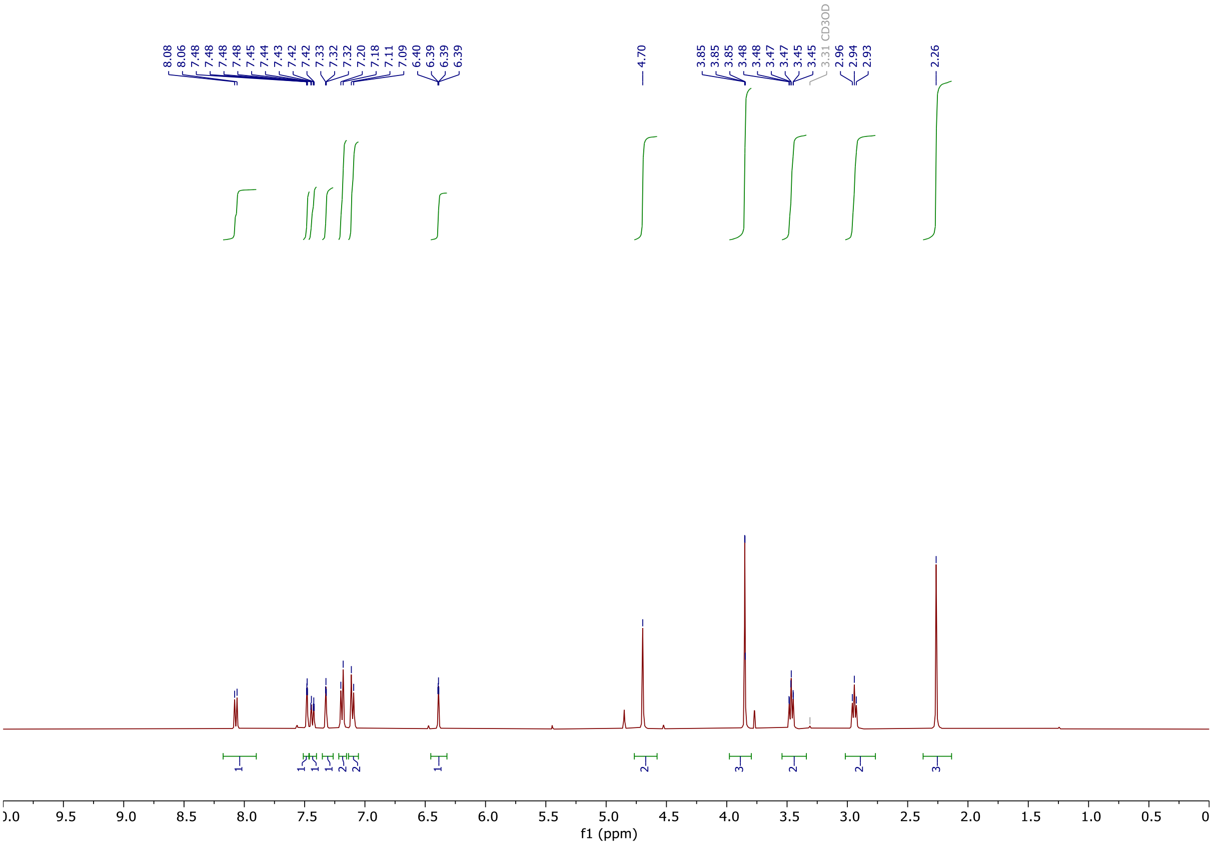

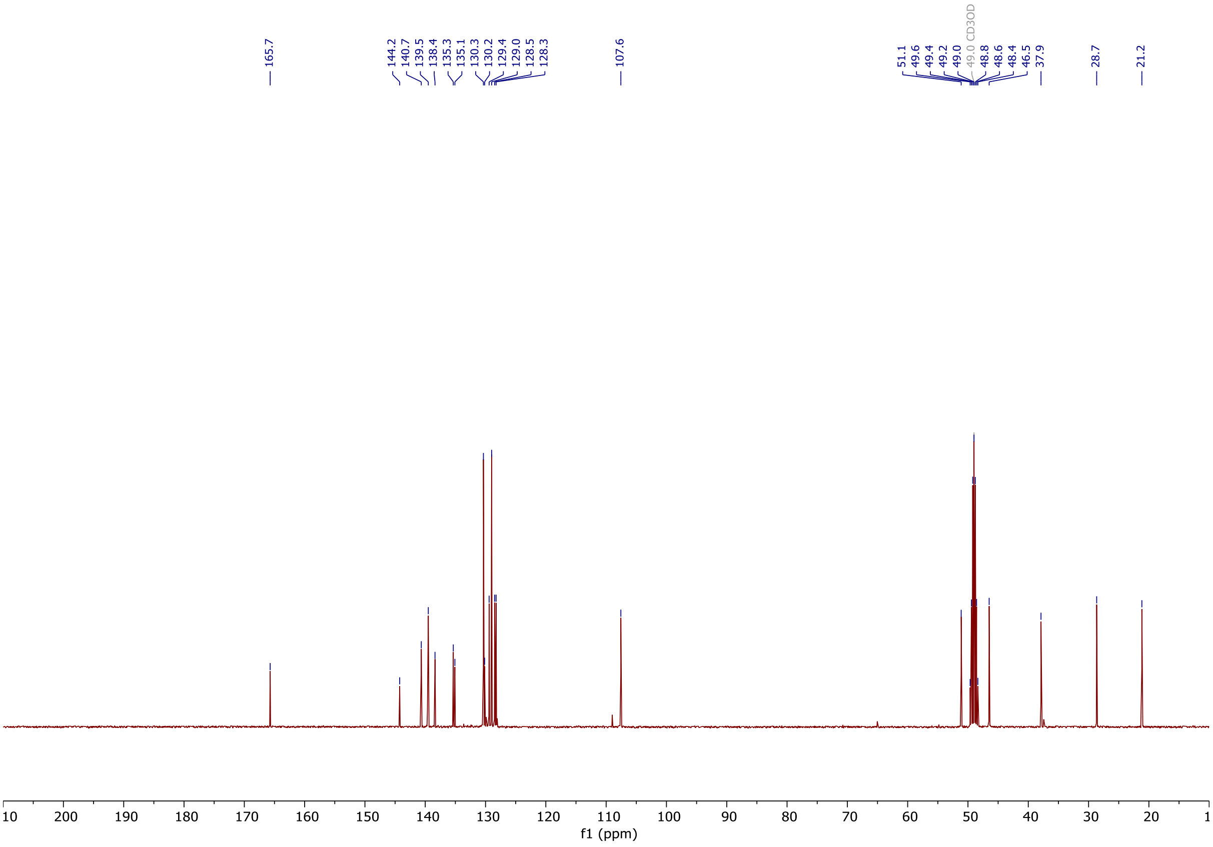

4238

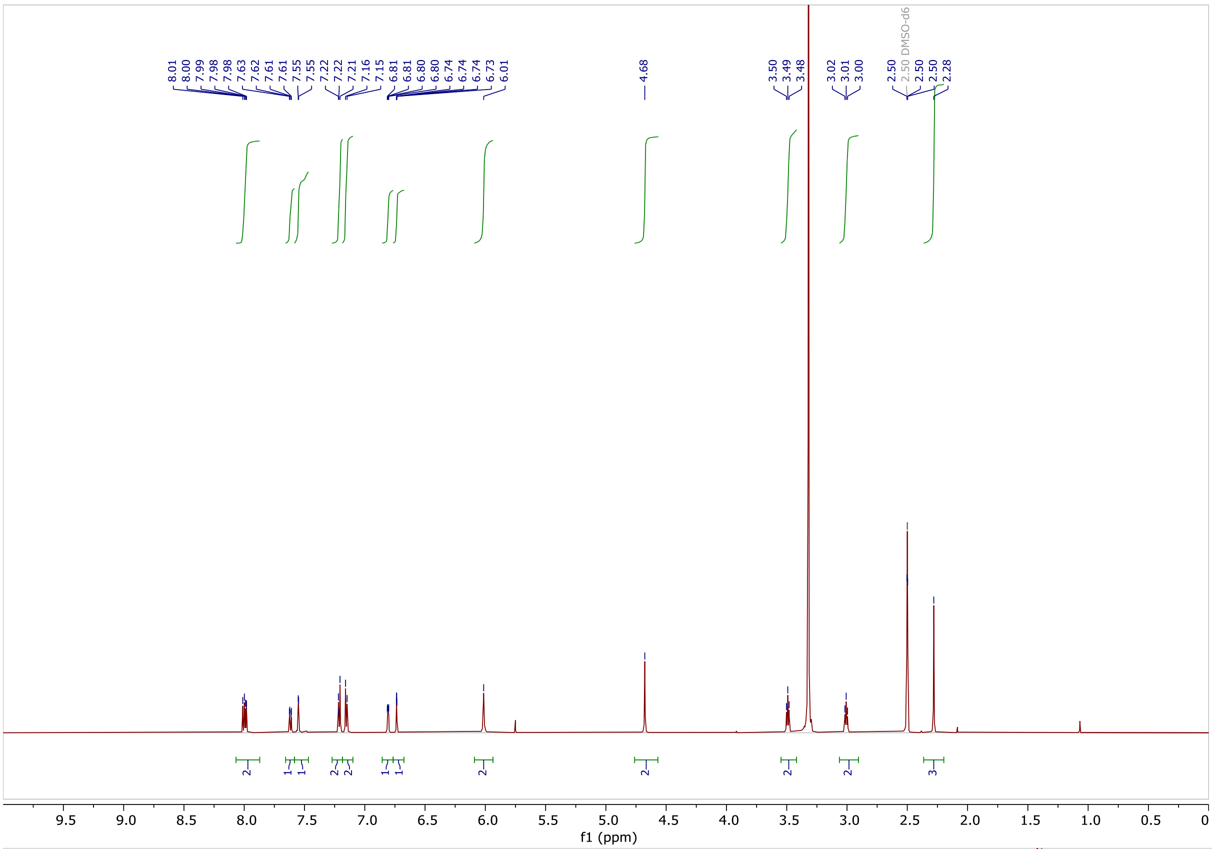
8.01
8.00
7.99
7.98
7.98
7.63
7.62
7.61
7.61
7.55
7.55
7.22
7.22
7.21
7.16
7.15
6.81
6.81
6.80
6.80
6.74
6.74
6.74
6.73
6.01
4.68
3.50
3.49
3.48
3.02
3.01
3.00
2.50
2.50 DMSO-d6
2.50
2.50
2.28
2
1
1
2
2
1
1
2
2
2
2
2
3
9.5
9.0
8.5
8.0
7.5
7.0
6.5
6.0
5.5
5.0
4.5
4.0
3.5
3.0
2.5
2.0
1.5
1.0
0.5
0
f1 (ppm)

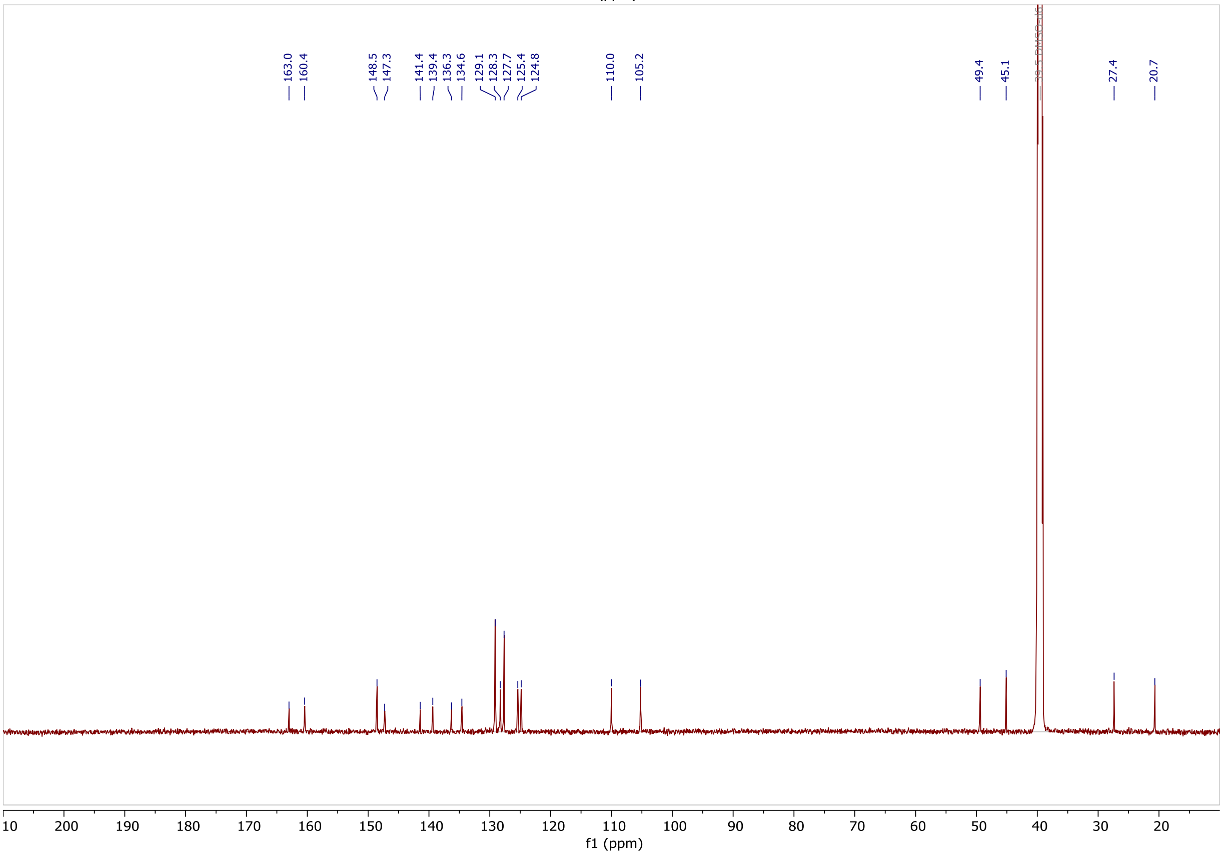
163.0
160.4
148.5
147.3
141.4
139.4
136.3
134.6
129.1
128.3
127.7
125.4
124.8
110.0
105.2
49.4
45.1
27.4
20.7
10
200
190
180
170
160
150
140
130
120
110
100
90
80
70
60
50
40
30
20
f1 (ppm)

4239

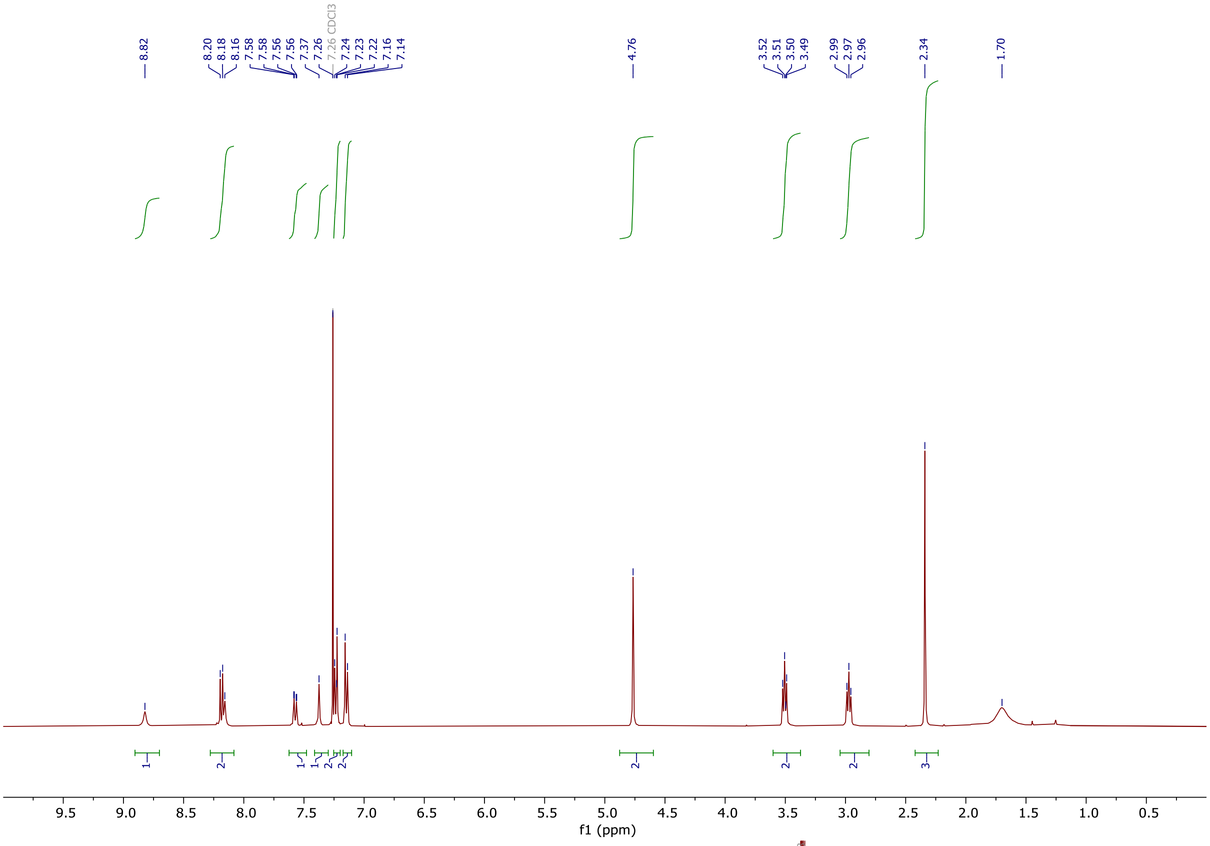
8.82
8.20
8.18
8.16
7.58
7.58
7.56
7.56
7.37
7.26
7.26 CDCl3
7.24
7.23
7.22
7.16
7.14
4.76
3.52
3.51
3.50
3.49
2.99
2.97
2.96
2.34
1.70
1
2
1
1
2
2
2
2
2
3
9.5
9.0
8.5
8.0
7.5
7.0
6.5
6.0
5.5
5.0
4.5
4.0
3.5
3.0
2.5
2.0
1.5
1.0
0.5
f1 (ppm)

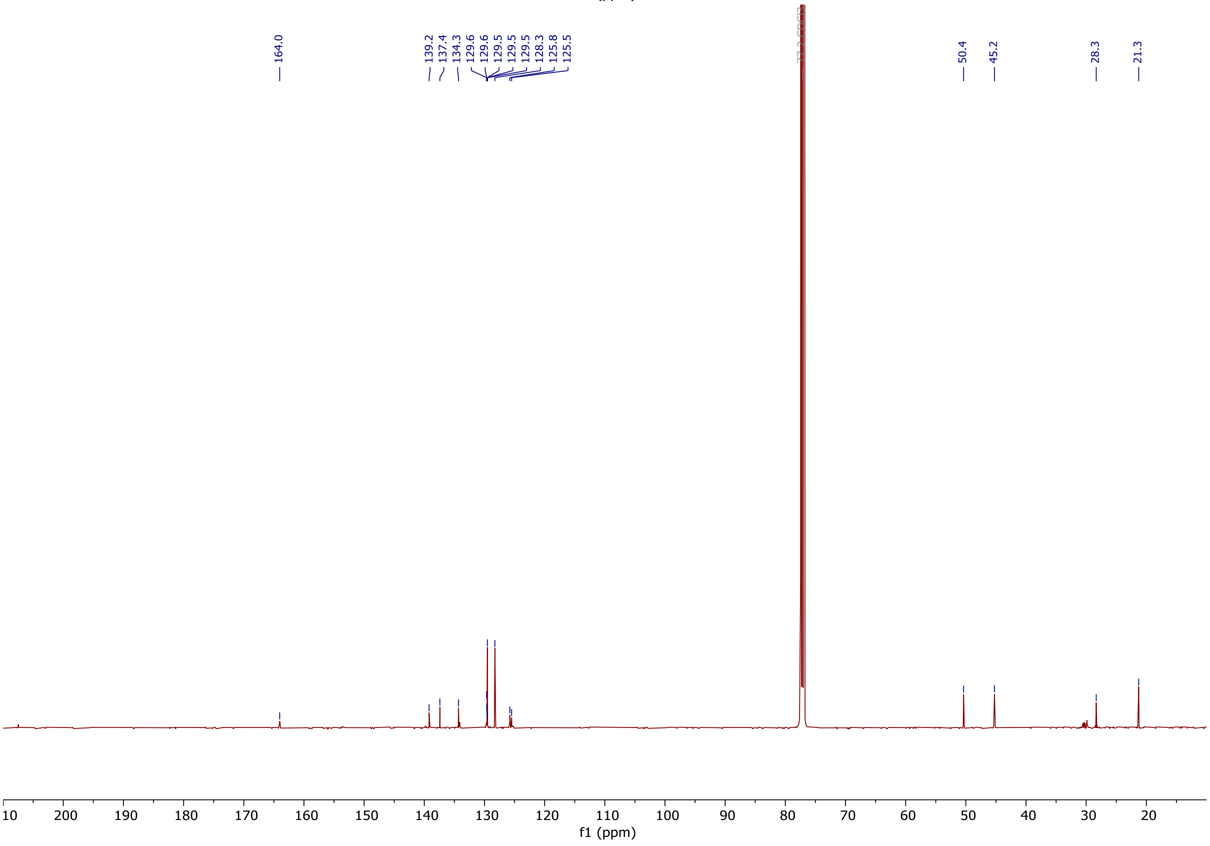
164.0
139.2
137.4
134.3
129.6
129.6
129.5
129.5
129.5
128.3
125.8
125.5
50.4
45.2
28.3
21.3
10
200
190
180
170
160
150
140
130
120
110
100
90
80
70
60
50
40
30
20
f1 (ppm)

References

Coppola, G.M., Damon, R.E., Kukkola, P.J., Stanton, J.L., 2004. Amide derivatives and their use as inhibitors of 11-beta-hydroxysteroid dehydrogenase type 1. WO2004065351A1.

Partridge, F.A., Murphy, E.A., Willis, N.J., Bataille, C.J.R., Forman, R., Heyer-Chauhan, N., Marinič, B., Sowood, D.J.C., Wynne, G.M., Else, K.J., Russell, A.J., Sattelle, D.B., 2017. Dihydrobenz[*e*][1,4]oxazepin-2(3*H*)-ones, a new anthelmintic chemotype immobilising whipworm and reducing infectivity *in vivo*. PLoS Negl Trop Dis 11, e0005359. https://doi.org/10.1371/journal.pntd.0005359

Zablocki, J.A., Elzein, E., Li, X., Koltun, D.O., Parkhill, E.Q., Kobayashi, T., Martinez, R., Corkey, B., Jiang, H., Perry, T., Kalla, R., Notte, G.T., Saunders, O., Graupe, M., Lu, Y., Venkataramani, C., Guerrero, J., Perry, J., Osier, M., Strickley, R., Liu, G., Wang, W.-Q., Hu, L., Li, X.-J., El-Bizri, N., Hirakawa, R., Kahlig, K., Xie, C., Li, C.H., Dhalla, A.K., Rajamani, S., Mollova, N., Soohoo, D., Lepist, E.-I., Murray, B., Rhodes, G., Belardinelli, L., Desai, M.C., 2016. Discovery of Dihydrobenzoxazepinone (GS-6615) Late Sodium Current Inhibitor (Late Na i), a Phase II Agent with Demonstrated Preclinical Anti-Ischemic and Antiarrhythmic Properties. Journal of Medicinal Chemistry acs.jmedchem.6b00939. https://doi.org/10.1021/acs.jmedchem.6b00939

Supplementary Figure 3

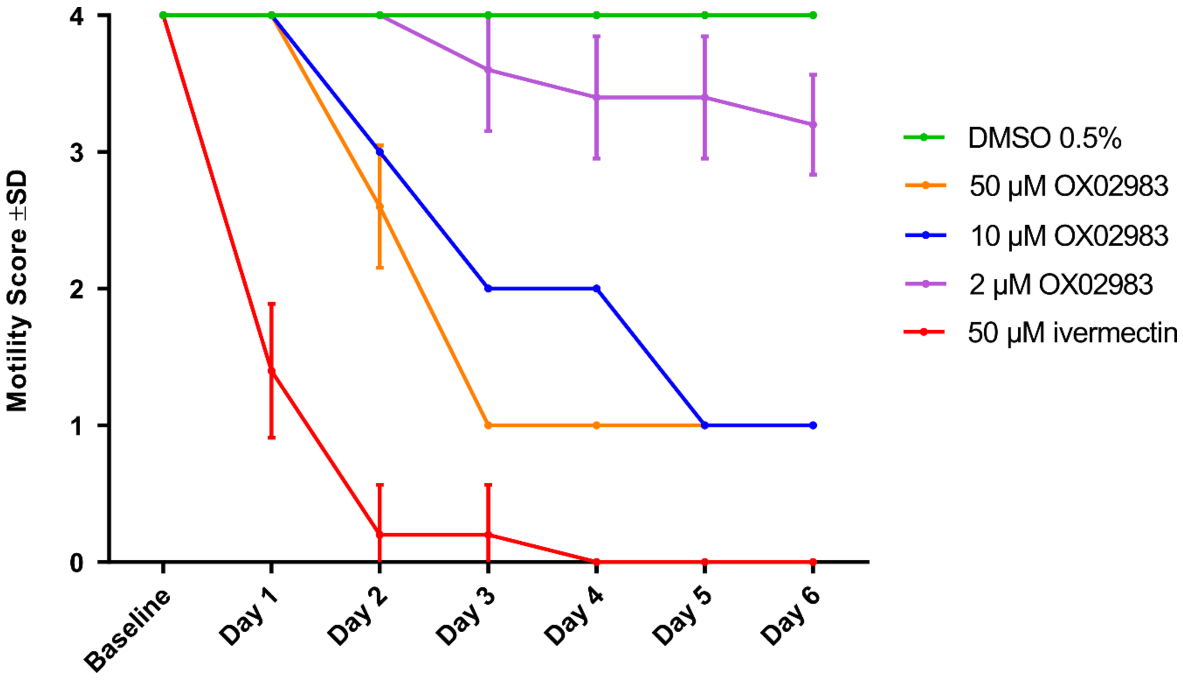

Supplementary Figure 3. Time course of *Brugia malayi* microfilariae motility scores taken every 24 hours for 6 consecutive days after exposure to compound OX02983. Error bars indicate mean plus or minus standard deviation.
